# The Knockout of PEX11a Results in Mild Peroxisomal Dysfunction and Lowered Cardiac Recovery Following Langendorff-Mediated Ischemia–Reperfusion in Mice

**DOI:** 10.3390/cells15010012

**Published:** 2025-12-20

**Authors:** Claudia Colasante, Jiangping Chen, Vannuruswamy Garikapati, Bernhard Spengler, Klaus-Dieter Schlüter, Eveline Baumgart-Vogt

**Affiliations:** 1 Institute for Anatomy and Cell Biology, Justus Liebig University, Aulweg 123, 35392 Giessen, Germany; jiangping.chen@anatomie.med.uni-giessen.de (J.C.); garikapa@mpi-cbg.de (V.G.); 2 Medical Clinic and Polyclinic II, UKGM Giessen, Klinikstraße 33, 35392 Giessen, Germany; 3 Max Planck Institute of Molecular Cell Biology and Genetics, Pfotenhauerstrasse 108, 01307 Dresden, Germany; 4 Institute of Inorganic and Analytical Chemistry, Justus Liebig University, Heinrich-Buff-Ring 17, 35392 Giessen, Germany; bernhard.spengler@anorg.chemie.uni-giessen.de; 5 Institute for Physiology, Justus Liebig University, Aulweg 129, 35392 Giessen, Germany; klaus-dieter.schlueter@physiologie.med.uni-giessen.de

**Keywords:** cardiomyocyte, peroxisome, ischemia, oxidative stress, lipid metabolism, MALDI imaging

## Abstract

**Highlights:**

**What are the main findings?**

- Cardiomyocytes from Pex11a^−/−^ mice display altered morphology and lipid composition.
- After in vitro ischemia/reperfusion, the recovery of Pex11a^−/−^ hearts was lowered.

**What are the implications of the main findings?**

- The precise function of peroxisomes in cardiomyocytes requires further elucidation.
- Peroxisomes might preserve cardiomyocyte functionality during and after ischemia/reperfusion injury.

**Abstract:**

Peroxisomal biogenesis defects frequently trigger processes of remodeling, increased oxidative stress and metabolic dysregulations that cause cellular dysfunction. Despite extensive research into cardiomyocyte ultrastructure and metabolism, knowledge on peroxisomal function in these cells is scarce. The objective of this study was therefore to investigate the impact of the purportedly asymptomatic (mild) deficiency of the peroxisomal biogenesis protein PEX11a on cardiomyocyte structure and cardiac function in mice. Langendorff-reperfusion experiments revealed diminished post-ischemic recovery following *Pex11a* knockout suggesting compromised cardiac response to ischemic stress. The suboptimal recovery might be attributable to increased ischemia-induced tissue deterioration consequent to morphological and metabolic abnormalities of the cardiomyocytes. Indeed, several alterations were observed in these cells in *Pex11a* knockout mice: (i) augmented size and number of peroxisomes and lipid droplets; (ii) increased sarcomere length; (iii) altered gene expression of peroxisome proliferator-activated receptors, organellar fission machinery proteins and cardiac markers; and (iv) a lipid composition shift. We hypothesized that peroxisomes contribute to the preservation of cardiomyocyte structure and functionality under conditions of ischemia–reperfusion. We further proposed that even “mild”, undiagnosed peroxisomal defects can significantly impact cardiac performance following ischemia. This poses novel challenges for the risk assessment of cardiac pathologies.

## 1. Introduction

Myocardial degeneration, which ultimately results in heart failure, is the consequence of cellular and molecular processes that modify cardiomyocyte functionality and metabolism [1,2]. In particular, the remodeling of mitochondrial energy, fatty acid and reactive oxygen species (ROS) metabolism has been associated with the onset of cardiomyocyte dysfunction [2,3,4,5,6,7]. Peroxisomes are single membrane-bound, intracellular organelles that contain enzymes catalyzing a variety of metabolic reactions [8], including, like mitochondria, the degradation of ROS and fatty acids [3,8,9,10]. However, because they do not directly contribute to energy production, peroxisomal functions in cardiomyocyte metabolism have been poorly studied. Nevertheless, the involvement of the peroxisomes in the development of cardiac pathologies necessitates further investigation, particularly in light of the following observations: (i) Patients with the inherited ‘milder’ peroxisomal disorder, adult Refsum’s disease (caused by the inability of peroxisomes to metabolize phytanic acid) develop arrhythmia, myocardial hypertrophy, and heart failure as they age [11]; (ii) secondary mitochondrial dysfunction has been demonstrated in humans and mice lacking functional peroxisomes [12,13,14]; (iii) experimental results suggest that in cardiomyocytes peroxisomes might control mitochondrial fatty acid oxidation through the metabolism of malonyl CoA [9]; (iv) peroxisome proliferator-activated receptors (PPARs) modulate both peroxisomal and mitochondrial metabolism [14]; (v) the use of PPAR-agonists, which also regulate peroxisomal metabolism, in the treatment of cardiovascular diseases has been well-documented [15,16,17]; (vi) peroxisomal β-oxidation controls the availability of ligands for PPARs [3] and (vii) fatty acid homeostasis is perturbed when peroxisomes are dysfunctional [18].

Peroxisomes are a class of single-membrane-bound organelles that are characterized by their versatility and adaptability [8,19,20,21]. Their abundance and protein composition vary between different cell types and tissues [3,22,23,24,25,26]. In heart tissue, peroxisomes have been initially identified during early electron microscopic studies as 0.2–0.5 μm large, oval organelles that are located at the junction between A- and I-bands of the sarcomere [27,28]. The number of peroxisomes is not only cell-type specific, but also dependent on a multitude of diverse stimuli [20,29]. These comprise fluctuations in nutrient availability, oxidative stress, and the administration of drugs [19,20,25,30,31,32,33]. In cardiomyocytes, the abundance of peroxisomes and the activity of catalase were increased when rats were fed on an ethanol-rich diet [34]. In addition, the myocardium of rats that were fed diets consisting of fish or rapeseed oil exhibited an increased number of peroxisomes, which were characterized by augmented size and elevated catalase activity [29,35]. Moreover, the treatment of rats with fenofibrate, a PPARa agonist, induced the activation of the cardiac peroxisomal β-oxidation [36]. Central to the process of peroxisome-adaptation is their proliferation through fission, which is coordinated by peroxins (PEX). In particular, PEX11a, b and g mediate the elongation and constriction of peroxisomes during fission [25,37,38,39].

We recently investigated the distribution of the three PEX11 proteins in the mouse myocardium and found that the left ventricle (LV) contained the highest expression of *Pex11a* and the largest number of peroxisomes [3]. We hypothesized that PEX11a may have an impact on peroxisome proliferation in the LV, which is the part of the heart that generates the highest force output. Therefore, we assumed that a peroxisomal defect caused by PEX11a depletion may have an effect on cardiac function [3].

In this study, the *Pex11a* general knockout mouse model was employed to study the impact of a purportedly asymptomatic (“mild”) peroxisomal biogenesis defect on cardiomyocyte structure and on cardiac function. The *Pex11a* knockout mice exhibit a much less pronounced symptomatic spectrum than the one observed in mouse models that mimic the Zellweger syndrome. The fact that *Pex11a* knockout mice reach adulthood renders them an optimal model for the study of long-term cardiac health risks associated with mild peroxisomal dysfunctions. To this purpose, we first performed experiments to investigate how the knockout of *Pex11a* influenced the morphology and protein and lipid composition of cardiomyocytes. Then, Langendorff reperfusion was utilized to assess the recovery capacity of *Pex11a* knockout hearts following ischemia/reperfusion (I/R) injury. The results indicate that the peroxisomal defect induced by the knockout of *Pex11a* caused remodeling of cardiomyocytes at the protein, mRNA, lipid and morphological level and reduced heart recovery following I/R injury.

## 2. Materials and Methods

### 2.1. Animals and Ethical Statement

All mouse experiments were approved by the German Government Commission of Animal Care (University internal classification: JLU-Nr.: 616_M, ProjectID: 1016 Peroxisomen). For this study, C57Bl/6J wild-type and B6.129-Pex11a^tm1Sjg^ (*Pex11a* knockout) mice were kindly provided by Dr. Xiaoling Li [40]. Mice were genotyped as previously described [40] using DNA from ear tissue and PCR with the primers P10, P11 and PNeo (Supplemental Table S1) as depicted in Supplementary Figure S1. Animals were housed under standard conditions (12 h light/dark cycle) with free access to food and water. Animals were anesthetized using 4% isoflurane for 2 min in an anesthesia box. When they displayed sufficient anesthetic depth, they were euthanized by cervical dislocation. For details, see Supplemental Materials.

### 2.2. RNA Isolation and RT-qPCR

Sacrificed animals were perfused for 30 s anterogradely with PBS through the left ventricle (LV). Following perfusion, the heart was dissected and shock-frozen in RNAzol^®^ (Sigma Aldrich Chemie GmbH, Steinheim, Germany). The total RNA was extracted from 50 mg of tissue. First-strand cDNA was synthesized from 1 µg total RNA using MultiScribe™ reverse-transcriptase (Thermo Fisher Scientific, Life Technologies GmbH, Darmstadt, Germany). For RT-qPCR, 1 µL 1:10-diluted cDNA and SsoAdvanced™ Universal SYBR^®^Green Supermix (Bio-Rad Laboratories GmbH, Feldkirchen, Germany) and the primer pairs listed in Supplemental Table S2 were used. Samples from 3 animals were run in duplicates, and the relative gene expression was calculated using b-actin as standard as previously described [41]. For details, see Supplemental Materials.

### 2.3. Western Blot Analyses

Sacrificed animals were perfused for 30 s anterogradely with PBS through the LV. The hearts were dissected and shock-frozen in 1 mL homogenisation buffer. For protein lysates, 100 mg of tissue were shredded and homogenized, followed by centrifugation to remove cell debris and nuclei. Proteins (10 µg) were separated on a 12% SDS-PAGE, transferred to PVDF-membranes by tank blotting and detected using the primary and secondary antibodies listed in Supplemental Tables S3 and S4. For details, see Supplemental Materials.

### 2.4. Paraffin Embedding for Histological Studies

Sacrificed mice were perfused for 1 min anterogradely with PBS through the LV, followed by 3 min perfusion using 4% paraformaldehyde (PFA), 2% sucrose/PBS. The hearts were dissected, immersion-fixed overnight in 4% PFA, 2% sucrose/PBS and embedded in paraffin using a Leica TP 1020 automated vacuum infiltration tissue processor (Leica, Wetzlar, Germany) as previously described [23]. Paraffin blocks were cut with a Leica RM2135 rotation microtome (Leica, Wetzlar, Germany).

### 2.5. Immunofluorescence Staining and Image Acquisition

For immunofluorescence analysis (IFA), 2 µm formaldehyde-fixed paraffin-embedded (FFPE) sections were deparaffinised, rehydrated and processed for antigen retrieval as previously described [23]. Sections were incubated using the primary and secondary antibodies listed in Supplemental Tables S3 and S5, respectively. Images were taken with a fluorescence microscope and processed using Photoshop_CS5. For details, see Supplemental Materials.

### 2.6. Histological Stainings

Several 5 µm FFPE sections were deparaffinised, rehydrated and stained according to standard protocols and processed for haematoxylin/eosin, azan and Picro-sirius-red stainings. For details, see Supplemental Materials.

### 2.7. Electron Microscopy (EM)

Sacrificed mice were perfused for 1 min anterogradely with PBS through the LV, followed by fixation in 4% PFA and 0.05% glutardialdehyde and processing either for standard-EM or immuno-EM using the catalase antibody (Supplemental Table S3) as previously described [12,24]. For details, see Supplemental Materials.

### 2.8. Langendorff Reperfusion

Langendorff reperfusion was performed as previously described [42], using 8 wild-type and 8 *Pex11a* knockout mice. For details, see Supplemental Materials.

### 2.9. Sample Preparation for MALDI MS Imaging

Hearts from wild-type and *Pex11a* knockout mice were dissected, embedded in gelatin, snap-frozen in liquid nitrogen and stored at −80 °C until further use. Frozen tissue sections were cut with an equal thickness of 15 μm at −20 °C with a cryostat, thaw-mounted on glass slides and dehydrated in a desiccator. Afterwards, the tissue surface was homogeneously sprayed with the matrix as previously described [43]. For details, see Supplemental Materials.

### 2.10. MALDI MS Imaging, Data Acquisition and Processing

After sample preparation, mass spectrometry imaging was performed using a high-resolution atmospheric-pressure scanning microprobe matrix-assisted laser desorption/ionization ion source coupled to a Fourier transform orbital trapping mass spectrometer as previously described [43]. For details, see Supplemental Materials.

### 2.11. Statistical Analysis

Morphometry of peroxisomes and lipid droplets from EM micrograph and immunofluorescence analysis images, measurements of sarcomere length, determination of staining intensity and stained areas in immunofluorescence analysis images, measurements on HE-stained tissue and densitometry analysis of Western blots were manually performed using the software ImageJ 1.53o.

Parameters related to the mice’s body and heart measurements and to morphometry on HE-stained heart cross sections and CSA were analyzed by ordinary two-way ANOVA Tukey, as indicated in the figure legends. Organelle and sarcomere morphometry, staining intensity and stained areas, Western blot densitometry and qRT-PCR results were evaluated using unpaired two-tailed Student’s *t*-test as indicated in the figure legend. Langendorff-reperfusion data were analyzed by ordinary two-way ANOVA, Tukey and unpaired two-tailed Student’s *t*-test. The data are presented as mean and standard error of the mean (SEM) and were analyzed using GraphPad Prism 9.5.0 (GraphPad Software, Boston, MA, USA). Corresponding *p*-values are indicated above the graphs and *p* ≤ 0.05 was considered statistically significant. Numbers of used animals and experimental replicates are indicated in the figures and figure legends.

## 3. Results

### 3.1. Cardiac Phenotype of the Pex11a Knockout Mouse

**Figure 1:**
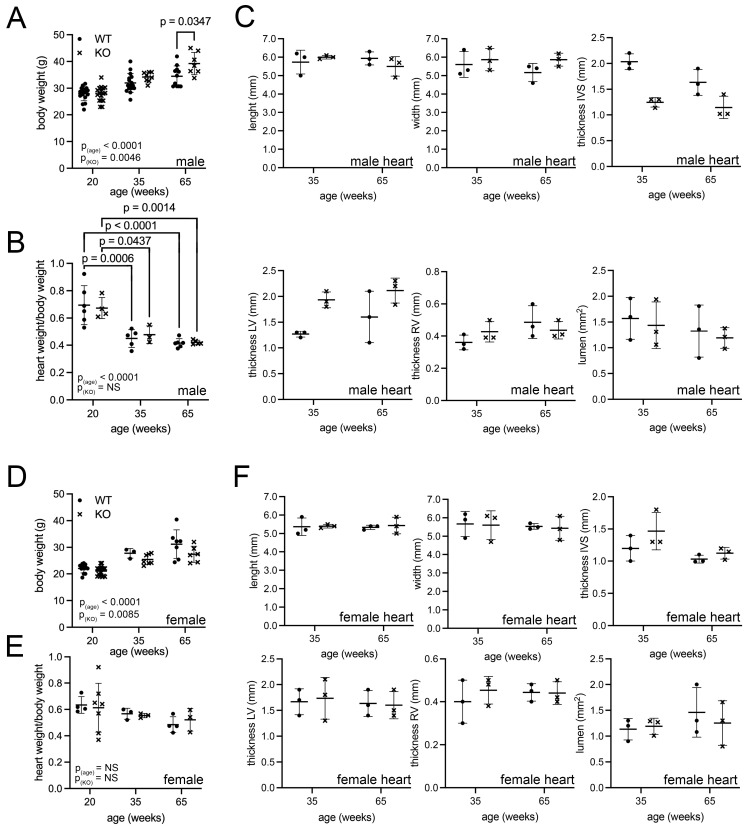
Cardiac phenotype of wild-type and *Pex11a* knockout mice. (**A**–**C**): Measurements in males. Bodyweight (n = indicated in the graph) (**A**), heart/bodyweight ratio (n = indicated in the graph) (**B**) and heart measurements (3 animals/genotype) (**C**). (**D**–**F**): Measurements in female mice. Body weight (n = indicated in the graph) (**D**), heart/body weight ratio (n = indicated in the graph) (**E**) and heart measurements (3 animals/genotype) (**F**). Heart measurements for male (**C**) and female (**F**) mice were performed on whole hearts (length and width) and heart sections (thickness of interventricular septum “IVS”; lumen area and thickness of right ventricle “RV” and left ventricle “LV”). The symbols **•** (wild type) and **x** (knockout) represent the value for one animal. Statistical analysis was performed using GraphPad and ordinary two-way ANOVA. The graphs represent mean and standard deviations. Significant (*p* < 0.05) *p*-values of multiple comparisons are indicated above the individual comparison. Main effects of age or knockout are indicated in the graphs. Abbreviations.: WT: wild-type *Pex11a* mice; KO: *Pex11a* knockout mice.

In comparison to other general *Pex* knockout mouse models, the *Pex11a* knockout mice display no obvious macroscopic alterations to the body, with the exception of a slightly lighter fur color [40]. We investigated the age-related changes in body and heart weight using 20, 35 and 65 weeks old mice (corresponding to 15, 30 and 50 years in human age according to Dutta et al. [44]). For both sexes and genotypes, a correlation between aging and increased bodyweight was found (Figure 1A,D). In the comparison between wild type and knockout, the *Pex11a*-knockout induced a minor yet significant increase in body weight in male mice aged 65 weeks (Figure 1A). In contrast, female *Pex11a*-knockout animals exhibited no genotype-related weight difference (Figure 1D). A significant age-related decrease in the heart to bodyweight ratio was equally observed in wild-type and knockout male, but not in female animals (Figure 1B,E).

**Figure 2:**
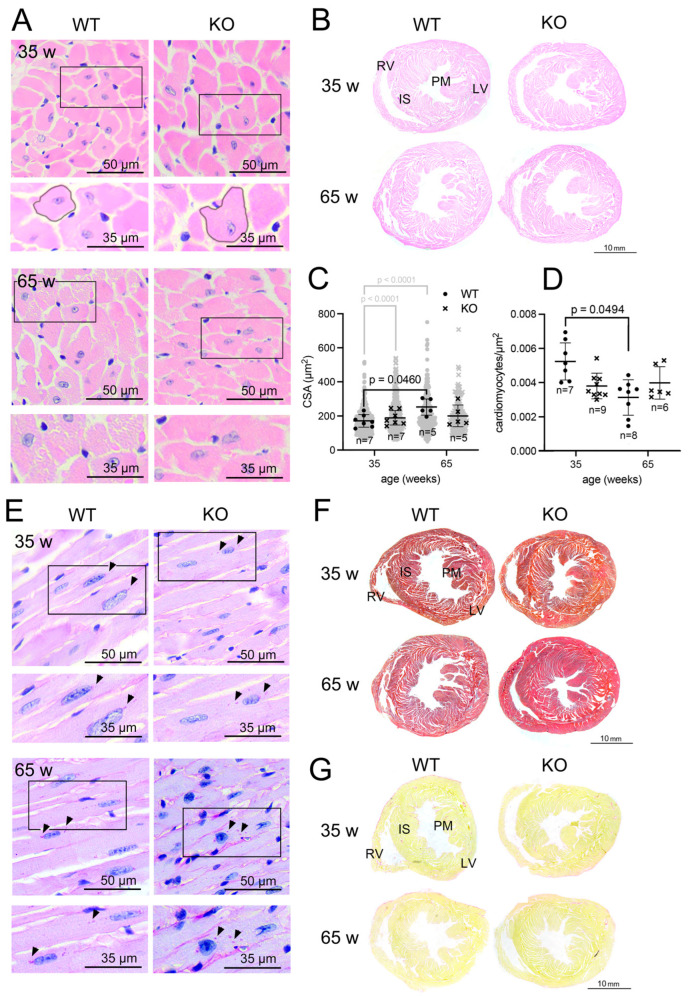
Histological analysis of heart tissue derived from wild-type and *Pex11a* knockout mice. (**A**) HE-stained cross sections of 35 and 65 weeks-old wild-type and knockout hearts. Lower rectangular images are enlargements of the square images shown above. Perimeter of cardiomyocytes of wild-type and knockout mice used to calculate the average cross sectional area (CSA) is outlined in black. (**B**): Midventricular plane cross sections of 35- and 65-week-old wild-type and knockout hearts stained with hematoxylin and eosin. (**C**,**D**): Cardiomyocyte average cross sectional area (CSA) (diagonally cut cardiomyocytes were omitted) (**C**) and number of cardiomyocytes/area (µm^2^) (**D**) calculated from images/genotype from different mice aged 35 and 65 weeks (n = indicated in the graph). The gray cloud in the background in (**C**) represents the CSA of each individual cardiomyocyte (350 individual cardiomyocytes for 35-week-old animals, 200 individual cardiomyocytes for 65-week-old animals, corresponding *p*-value is indicated in gray). Statistical analysis was performed using GraphPad and ordinary two-way ANOVA. The graphs represent the mean and standard deviations. Significant *p*-values (*p* < 0.05) of multiple comparisons are indicated above the individual comparison. (**E**): PAS-stained cross sections of 35- and 65-week-old wild-type and knockout hearts. Arrows indicate accumulations of glycogen. (**F**,**G**): Azan (**F**) and Picro-sirius-red (**G**) -stained cross sections of 35- and 65-week-old wild-type and knockout hearts. Scale bars are indicated in the images. Results are derived from male and female mice. Abbreviations: WT: *Pex11a* wild-type mice; KO: *Pex11a* knockout mice; w: weeks; RV: right ventricle; LV: left ventricle; IS: interventricular septum; PM: papillary muscle.

For a comparison between adult and aged adults, we next continued our analyses on the macroscopic and microscopic phenotype of the heart in 35- and 65-week-old mice. Measurements of whole heart length or width at the midventricular plane showed no significant differences between the two genotypes (Figure 1C,F). As exemplified in the HE-staining (Figure 2B), heart cross sections at the mid-ventricular plane also revealed no significant disparities in the myocardial thickness (left ventricular and right ventricular outer walls and interventricular septum) or in the dimensions of the lumen of the the left ventricle (LV) (Figure 1C,F). These results indicated that macroscopically, the hearts of wild-type and *Pex11a* knockout mice were indistinguishable.

Morphometric measurement of HE-stained cross sections of mouse LVs (Figure 2A) revealed a minor increase (1.2-fold) in the average cross sectional area (CSA) of the cardiomyocytes in 65-week-old wild-type mice, which correlated to a decrease (1.6-fold) of the cardiomyocyte/area (Figure 2C,D). In the 35-week-old knock-out hearts, a significant size increase was shown by the individual measurements for CSAs. Nevertheless, the discrepancy became non-significant when the mean CSA was compared for each individual mouse (Figure 2C). Moreover, in contrast to the wild-type mice, no age-related increase in CSA or augmentation of the number of cardiomyocytes/area was observed in the knockout animals.

Accumulation of glycogen in cardiomyocytes is an indicator for structural remodeling [45,46]. PAS-staining of wild-type and knockout hearts indicated no difference in the abundance of glycogen (Figure 2E). Furthermore, azan or picro-sirius-red stainings showed no collagen accumulations in the knockout hearts, indicating the absence of fibrotic remodeling (Figure 2F,G), irrespective of age and genotype.

### 3.2. The Pex11a Knockout Upregulates the Expression of Pex11b and Pex11g and Reduces the Transcript Abundance of Ppara

To clarify whether the knockout of *Pex11a* affected the expression of the other two members of the *Pex11* family (*Pex11b* and *Pex11g*), their mRNA transcripts were analyzed by RT-qPCR. As expected, the *Pex11a* knockout heart samples did not contain mRNA for *Pex11a* (Supplemental Figure S2A). Transcript abundance of *Pex11b* and *Pex11g* instead was significantly upregulated by the *Pex11a* knockout (Supplemental Figure S2A).

Subsequently, nuclear receptors regulating peroxisome proliferation, the peroxisome proliferator-activated receptors PPARa, PPARb and PPARg were investigated. These transcription factors bind to fatty acids and their derivates and respond to changes in nutritional status and oxidative stress to modulate glucose and lipid metabolism [47]. In contrast to the significantly lowered expression of *Ppara*, the one for *Pparb* was unchanged, and the one for *Pparg* was slightly, but not significantly, increased (*p* = 0.2) (Supplemental Figure S2B).

### 3.3. The Knockout of Pex11a Results in Enlarged Peroxisomes

Immunofluorescence analysis (IFA) of the LV was performed to investigate the abundance of peroxisomal marker proteins. For this purpose, we used antibodies that target catalase, a well-established peroxisomal marker, PEX14p and PEX19p. We have previously demonstrated that the integral membrane peroxin PEX14p reliably identifies and determines the number of peroxisomes in different types of cells [24]. For this reason, we routinely use it for morphometric analysis. The peroxin PEX19p instead was used as a marker to determine whether changes in peroxisome early assembly were occurring in the Pex11a knockout.

**Figure 3:**
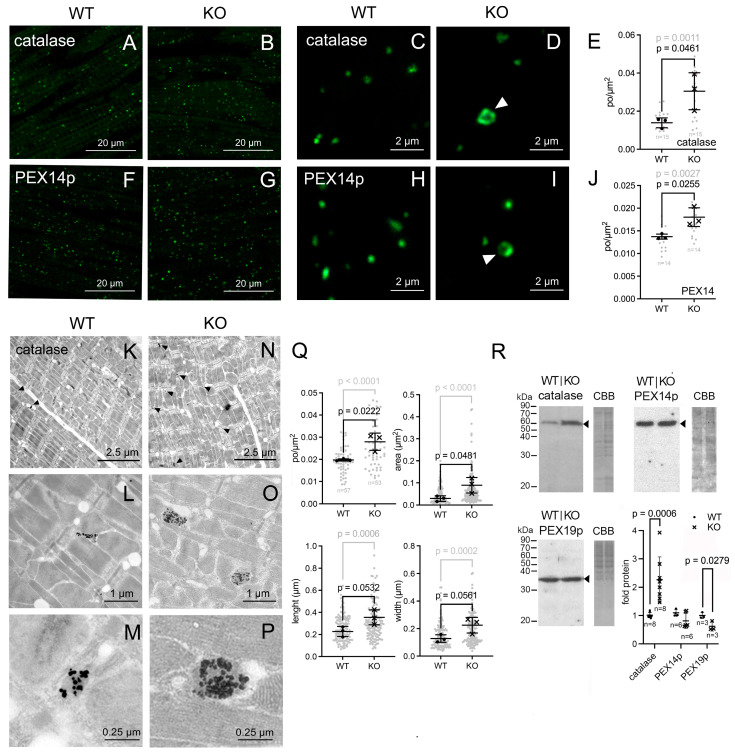
Peroxisomal protein content and morphological appearance in *Pex11a* knockout cardiomyocytes. (**A**–**J**): IFA of LVs of 35-week-old wild-type and knockout mice and peroxisomes/µm^2^ after labeling for catalase (**A**–**E**) and PEX14p (**F**–**J**). Images (**C**,**D**) and (**H**,**I**) show images of peroxisomes from wild-type and knockout mouse hearts at high magnification. White arrowheads indicate enlarged peroxisomes in the knockout. Peroxisome counting and tissue area definition in (**E**,**J**) were performed with ImageJ using images (n = indicated in the graph) from 3 mice/genotype. (**K**–**P**): Electron micrographs of immunogold-labeling for catalase to visualize peroxisomes of wild-type and knockout animals. (**Q**): Morphometry of catalase-labeled electron micrographs. Peroxisomes/µm^2^ were calculated from individual electron micrographs (n = indicated in the graph) from 3 different animals. Peroxisomal area, length and width were calculated from a total of 135 peroxisomes using electron micrographs from 3 mice/genotype. In (**E**,**J**,**Q**) symbols, “**•**” (wild-type) and “**x**” (knockout) represent average values for the 3 animals. The background gray clouds represent either values per individual image ((**E**,**J**) and “po/µm^2^” in (**Q**)) or per individually measured peroxisome (“area”, “length” and “width” in (**Q**)) with corresponding *p*-value (gray). (**R**): Western blot (10 µg protein) and densitometric analysis of wild-type and *Pex11a* knockout LVs performed using antibodies against catalase, PEX14p, and PEX19p. CBB staining for the entire lane is shown as a loading control. Densitometric analysis of Western blots of catalase, PEX14p, and PEX19p derived from the Western blots presented in this figure and additional Western blots (see original Western blot file). The number of investigated animals “n” is indicated in the graph. All morphometric and densitometric analyses were performed using ImageJ. Only values derived from samples loaded on the same blot were directly compared to each other and the WT value was set to 1. Statistical analyses were conducted with GraphPad Prism 9 using the unpaired *t*-test. The graphs represent mean and standard deviations. Significant *p*-values (*p* < 0.05) are indicated above the individual comparison. Scale bars are indicated in the images. Results derived from male and female mice. Abbreviations.: WT: wild-type *Pex11a* mice; KO: *Pex11a* knockout mice. WT: wild-type *Pex11a* mice; KO: *Pex11a* knockout mice.

In both genotypes, the immunofluorescence staining of the LV obtained using antibodies against catalase, PEX14p and PEX19p produced the typical dotted pattern observed for peroxisomes in cardiomyocytes (Figure 3A,B,F,G and Figure S3A,B). Morphometry of the peroxisome number revealed that in the *Pex11a* knockout mice, the number of catalase- (Figure 3A,B,E) and PEX14p-stained (Figure 3F,G,J) peroxisomes was significantly elevated. A nearly significant increase in the number of peroxisomes was also observed in the *Pex11a* knockout using an antibody against PEX19p (Supplemental Figure S3A,B,E). In the *Pex11a* knockout, independently of the used marker, morphologically aberrant, enlarged peroxisomes displaying a dark central region were identified (Figure 3C,D,H,I and Figure S3C,D) [3].

To investigate peroxisome morphology and intracellular distribution in the cardiomyocytes of the LV, we performed electron microscopy analyses using catalase immunolabelling. The electron micrographs revealed gold-marked oval structures, which were identified as peroxisomes (Figure 3K–P). The peroxisomes were interspersed amongst the mitochondria between the myofibrils in proximity to the Z-line (Figure 3K–O). In the *Pex11a* knockout, very large peroxisomes with a central area lacking catalase-labeled gold particles (Figure 3M,P), which resembled the ring-shaped peroxisomes identified using IFA, were also observed. Morphometry revealed that the cardiomyocytes of the *Pex11a* knockout mice harbored larger peroxisomes than their wild-type counterparts (Figure 3Q). The *Pex11a* knockout peroxisomes displayed wider size variation (0.2–0.4 µm^2^) with a subpopulation of significantly enlarged organelles (20% > 0.2 µm^2^) while in wild-type, none was >0.2 µm^2^ (Figure 3Q). Despite this size increment, Western blot analysis of peroxisomal marker proteins showed that only the abundance of catalase was significantly increased in the *Pex11a* knockout LV (Figure 3R).

Based on these observations, we suspected a defect of peroxisome homeostasis. Proteins involved in mitochondrial and peroxisomal fission have been proposed to interact with PEX11 proteins [37]. To investigate whether the removal of *Pex11a* deregulates the expression of pexophagy and peroxisomal fission-related genes, their mRNA abundance was analyzed using RT-qPCR. The results showed that the expression of *Mff1*, *Atg7* and *Fis1* was significantly downregulated, while that of *p62* was significantly elevated after the knockout of *Pex11a* (Supplemental Figure S4A,B).

### 3.4. Indications for Altered Cardiomyocyte Structure in the Pex11a Knockout Hearts

**Figure 4:**
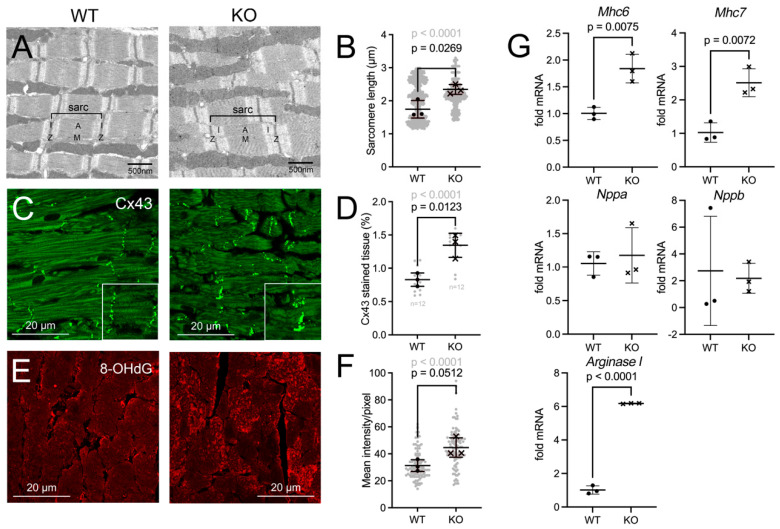
Altered cardiomyocytes in the *Pex11a* knockout hearts. (**A**,**B**): Electron micrograph (**A**) and morphometry (**B**) of sarcomere length in wild-type and knockout LVs. 660 sarcomeres from 3 animals/genotype were measured. (**C**,**D**): IFA of LVs from wild-type and knockout hearts using a Cx43 antibody (**C**) and corresponding morphometry (Cx43-covered tissue area from a total of 12 images from 3 mice/genotype) (**D**). (**E**,**F**): IFA of LVs from wild-type and knockout hearts using an 8OHdG antibody (**E**) and corresponding staining intensity calculated from a total of 100 cells from 3 mice/genotype (**F**). For (**B**,**D**,**F**) symbols, “**•**” (wild-type) and “**x**” (knockout) represent average values for the 3 animals. The background gray cloud represents individual values per analyzed sarcomere (**B**), image (**D**) or cardiomyocyte (**F**) with corresponding *p*-value (gray). (**G**): mRNA expression of cardiac marker genes. cDNA derived from LV mRNA from 3 mice/genotype was used for RT-qPCR. Wild-type CT value was set to one. Morphometry was performed using ImageJ. Statistical analyses were conducted with GraphPad Prism 9 using the unpaired *t*-test. The graphs represent mean and standard deviations. Significant *p*-values (*p* < 0.05) are indicated above the individual comparison. Scale bars are indicated in the images. Results are derived from male and female mice. Abbreviations: wild-type: WT *Pex11a* mice; KO: *Pex11a* knockout mice; sarc: sarcomere; A and I: A-band and I-band; Z: M: M-line; Z-line.

Morphometry of electron microscopy micrographs of LVs revealed that in wild-type cardiomyocytes, the length of the individual sarcomeres ranged between 1.1 and 2.9 µm (Figure 4A,B). This corresponds well to the size (1.8–2.2 µm) reported in the literature [48]. With a size range of 1.4–3.3 µm, the sarcomeres of the *Pex11a* knockout hearts appeared longer (Figure 4B). For both genotypes, three main sarcomere subpopulations were detected: short (1.1–1.7 µm), medium (1.8–2.5 µm) and long (2.6–2.9 µm) (Figure 4B). After quantification, 35%, 32% and 33% of wild-type and 14%, 61% and 18% of knockout cardiomyocytes, respectively, corresponded to the categories short, medium, and long. In the knockout, a fourth subpopulation of very long sarcomeres was identified with a size range of 3–3.3 µm, representing 7% of the whole population (Figure 4B).

Despite the absence of obvious ultrastructural remodeling of the intercalated disks as evidenced by electron microscopy, the gap junctions were investigated by IFA using an antibody against connexin 43 (Cx43) (Figure 4C). As expected, Cx43 localized at the short end of the cardiomyocytes at the intercalated disks (Figure 4C and 2× magnified insets therein). In the *Pex11a*-deficient heart, the fluorescent signal for Cx43 was slightly more intense in a subset of cardiomyocytes (Figure 4C and insets therein). Morphometry of the percentage of tissue area covered by Cx43 revealed that the protein was more widely distributed in the *Pex11a*-knockout heart (Figure 4D).

Peroxisomal defects usually increase the intracellular concentration of ROS. 8-hydroxydesoxyguanosin (8OHdG) is a nucleotide-modification that is common in mitochondria when they are subjected to elevated oxidative stress [49]. Staining LVs of wild-type mice revealed only a little 8OHdG staining. However, staining the myocardium of the knockout mice demonstrated the presence of 8OHdG in mitochondria (Figure 4E). Quantitative analysis confirmed that in the *Pex11a* knockout LVs 8OHdG was elevated (Figure 4F). To test whether changes to the abundance of mitochondrial electron transport chain (ETC) complexes and of SOD2 occurred in cardiomyocytes of the knockout mice, IFA was used. The results show that while the fluorescent signal for SOD2 (Supplemental Figure S5A–C), cytochrome c (CYC1) (Supplemental Figure S5J–L) and succinate dehydrogenase (Complex II, SDH) (Supplemental Figure S5G–I) was increased, the one for Complex IV (Supplemental Figure S5M–O) and I (Supplemental Figure S5D,F) was decreased in the knockout.

It was further investigated whether the observed tissue alterations were associated with changed expression of marker genes for hypertrophy, hypoxic stress and contractile dysfunction. The most prominent difference was a 4.5-fold increment in the expression of *Arginase I* in the knockout heart. Also, the expression of *Mhc6* and *Mhc7* was significantly increased (Figure 4G).

### 3.5. The Lipid Composition of the Myocardium Is Altered in the Pex11a Knockout Mice

**Figure 5:**
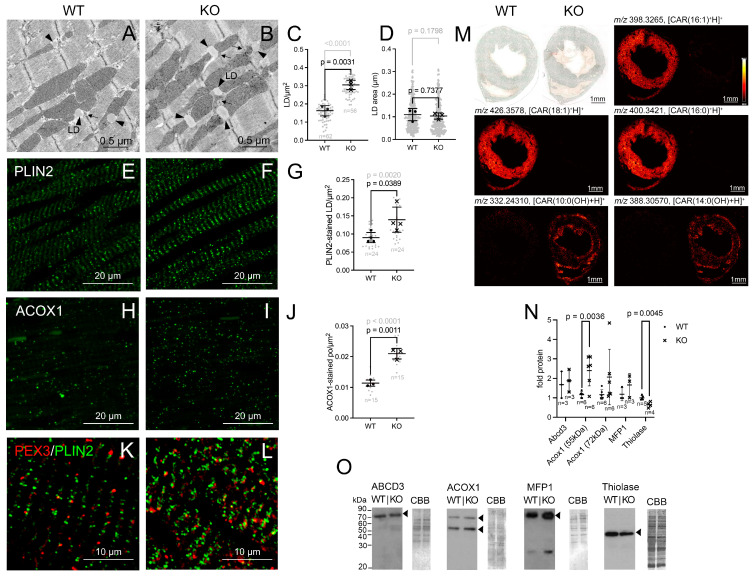
Lipid storage and metabolism are compromised in the hearts of the *Pex11a* knockout mice. (**A**,**B**): Electron micrographs of LDs (black arrowheads) and POs (black arrows) in LV cardiomyocytes of wild-type and knockout mice. (**C**,**D**): LD abundance (**C**) (calculated from “n” images as indicated in the graph) and size (**D**) (calculated from 233 LDs) derived from 3 mice/genotype. (**E**,**F**): IFA of PLIN2 in LVs. (**G**): Abundance of PLIN2-stained LDs in wild-type and knockout LVs calculated from 4 mice/genotype (“n” images as indicated in the graph). (**H**,**I**): IFA of ACOX1 in wild-type and knockout LVs. (**J**): Morphometry of ACOX1-stained peroxisomes in wild-type and knockout LVs from 3 mice/genotype (“n” images as indicated in the graph). For (**C**,**D**,**G**,**J**) symbols, “**•**” (wild-type) and “**x**” (knockout) represent average values for 3 animals. The background gray cloud represents the values per analyzed image (**C**,**G**,**J**) or measured LD (**D**) (corresponding *p*-value in gray). (**K**,**L**): IFA of PEX3p and PLIN2 in the LVs of wild-type (**K**) and knockout (**L**) mice. (**M**): High-resolution MALDI MS-imaging of selected endogenous metabolites (tentative assignments) in wild-type and knockout hearts. (**N**): Densitometric analysis of Western blots of ABCD3, ACOX1, MFP1 and Thiolase (n = number of investigated animals) from the WB shown in U and additional Western blots (see original Western blot file). Only values derived from samples loaded on the same blot were directly compared to each other and the WT value was set to 1. (**O**): Western blot (10 µg protein) of wild-type and *Pex11a* knockout mice LVs performed using ABCD3, ACOX1, MFP1 and thiolase antibodies. CBB staining for the entire lane is shown as loading control. All morphometric and densitometric analyses were performed using ImageJ. Statistical analyses were conducted with GraphPad Prism 9 using the unpaired *t*-test. The graphs represent mean and standard deviations. Significant *p*-values (*p* < 0.05) are indicated above the individual comparison. Scale bars are indicated in the images. Results are derived from male and female mice. Abbreviations.: WT: wild-type *Pex11a* mice; KO: *Pex11a* knockout mice; POs: peroxisome; LD: lipid droplets; CAR: Acylcarnitines; *m*/*z*: mass-to-charge-number ratio.

Electron micrographs of LVs revealed an increase in the number of lipid droplets (LDs) in the cardiomyocytes of the *Pex11a* knockout mice (Figure 5A–C). The LDs appeared near mitochondria as oval structures with low electron density, no surrounding membrane and an average size of 0.1 µm^2^ (Figure 5A,B,D). Morphometry revealed a significantly elevated number of LDs with increased size variability in the heart of *Pex11a* knockout mice (Figure 5A–D). These observations were further confirmed by IFA of perilipin 2 (PLIN2), a protein located on the LD surface (Figure 5E,F). Quantification of the number of PLIN2-positive LDs revealed a significant increase in the *Pex11a* knockout heart (Figure 5G). PLIN2-stained LDs were located near peroxisomes marked with PEX3p, indicating the proximity of the two structures (Figure 5K,L). This can also be observed in the EM images (Figure 5A,B).

IFA and Western blot analysis further indicated that peroxisomal proteins involved in fatty acid degradation were dysregulated: ACOX1 was significantly increased in the *Pex11a* knockout (Figure 5H–J,N,O) while 3-ketoacyl-CoA-thiolase (thiolase/ACAA1) was significantly downregulated (Figure 5N,O). Accordingly, the number of ACOX1-stained peroxisomes was increased in the *Pex11a* knockout (Figure 5H–J).

These results led to the supposition that the *Pex11a* knockout might disturb the cardiomyocyte’s lipid metabolism. Therefore, the localization of differentially abundant lipids in heart cross sections of wild-type and *Pex11a* knockout mice was investigated using high-resolution MALDI MS imaging (Figure 5M and Figure S6, Supplemental Table S6). MALDI MS ion images (hot color map, linear interpolation of zero order, TIC normalization) and their relative signal intensities quantification highlighted the specific differences in the lipid profile between the investigated genotypes (Figure 5M and Figure S6, Supplemental Table S6). The following lipids were altered by the *Pex11a* knockout: (i) acylcarnitines (fatty acylesters of carnitine, CARs); (ii) glycerophospholipids, including phosphatidylcholine and lysophosphatidylcholine (PC, LPC); phosphatidylserine (PS); phosphatidylethanolamine and lysophosphatidylethanolamine (PE, LPE); and (iii) glycerolipids including di- and triglycerides (DG and TG, respectively).

The most prominent differentially abundant lipids were the CARs. In total, MALDI-MS imaging detected 15 differentially abundant CARs, of which 10 exhibited relatively lower signal intensities in the *Pex11a* knockout heart sections (Figure 5M and Figure S6, Supplemental Table S6). Of the identified 15 CARs, 5 were hydroxyacylcarnitines with chain length between 8 and 14 carbons. Interestingly, all hydroxyacylcarnitines exhibited higher signal intensities in the *Pex11a* knockout heart (Figure 5M and Figure S6, Supplemental Table S6). Surprisingly, triglycerides were less abundant in the *Pex11a* knockout mouse heart in comparison to the wild-type controls.

Noteworthy, the MALDI-MS ion images revealed a differential distribution of lipids within the heart sections. CARs were homogeneously distributed in the myocardium except for the carnitine ester of myristic acid (*m*/*z* 372.3108), which appeared concentrated on the LV lateral wall (Supplemental Figure S6). Instead, the hydroxyacylcarnitines accumulated preferentially at the borders of the heart sections corresponding to endocardium and epicardium (Figure 5M and Figure S6).

### 3.6. Hearts of Pex11a Knockout Mice Displayed Decreased Performance Recovery Following Ischemia–Reperfusion (I/R) Injury

**Figure 6:**
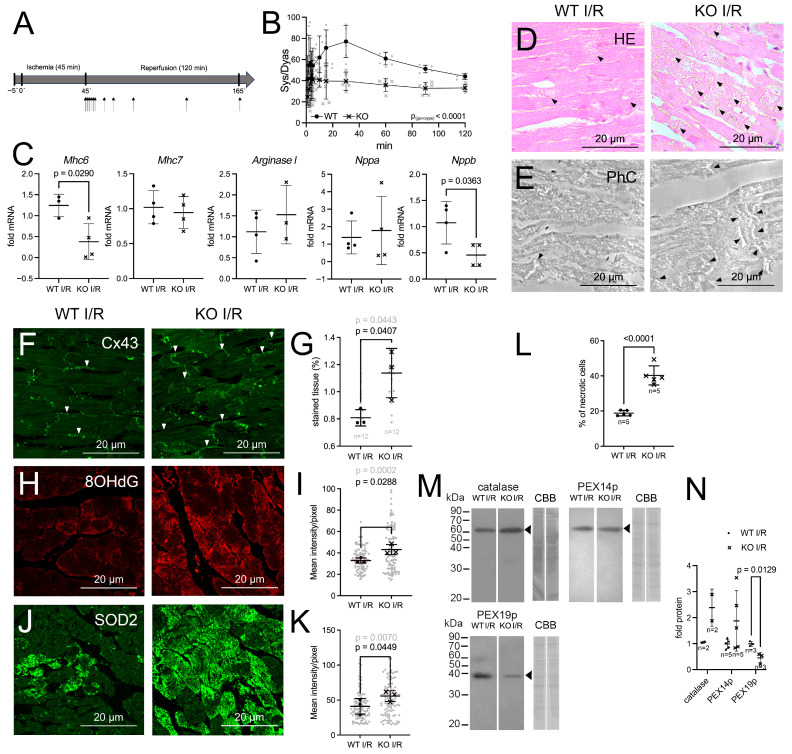
Hearts of *Pex11a* knockout mice display reduced recovery and increased damage following I/R injury. (**A**,**B**): Time course of Sys/Dyas following I/R injury in hearts of wild-type and knockout hearts. Perfused hearts were left to stabilize for 5 min. Ischemia was induced for 45 min by stopping flow and pacing. After 120 min the reperfusion was stopped (**A**). Arrows in (**A**) indicate time points of measurements. Values were calculated from 8 hearts/genotype. (**C**): mRNA expression of cardiac marker genes. cDNA was derived from LV mRNA from 3 or 4 mice/genotype subjected to I/R injury. The wild-type CT value was set to one. (**D**,**E**): Damage of wild-type and knockout heart tissue following I/R injury in HE-stained (**D**) and phase contrast (**E**) images of LV sections. Black arrows indicate contraction band necrosis. (**F**,**H**,**J**): IFA of the LVs from I/R-injured wild-type and knockout hearts using Cx43 (**F**), 8OHdG (**H**) and SOD2 (**J**) antibodies. (**G**): Percentage of Cx43-stained area (white arrows in (**F**)) in wild-type and knockout LVs from 3 mice/genotype (4 images/mice). (**I**,**K**): Mean intensity/pixel of 8OHdG (**I**) and SOD2 (**K**) calculated using the image acquisition program Zen_2.3_sp1 from 100 cells from 3 mice/genotype. (**L**): Percentage of structurally damaged cardiomyocytes determined from HE-stained images from I/R-injured wild-type and knockout hearts (700 cells from 5 different sections from 3 mice/genotype). (**M**): Western blot (10 µg protein) from I/R-injured wild-type and knockout LVs performed using catalase, PEX14p, and PEX19p antibodies. CBB staining for the entire lane is shown as loading control. (**N**): Densitometric analysis of Western blots of catalase (n = 2), PEX14p (n = 5), and PEX19p (n = 3) (n = number of mice) from the WB shown in U and additional Western blots. Only values derived from samples loaded on the same blot were directly compared to each other, and the WT value was set to 1. The lanes in this figure were taken from the same gel, but non-adjacent lanes were cropped together as indicated by the white lines separating the lanes (please refer to the supplemental Western blots for original images). Significant *p*-values (*p* < 0.05) are indicated above the individual comparisons. Main effect of the genotype is indicated in the graph in (**A**). Scale bars are indicated in the images. Results are derived from male and female mice. Abbreviations.: WT: wild-type *Pex11a* mice; KO: *Pex11a* knockout mice; wild-type I/R: wild-type *Pex11a* heart subjected to I/R injury; knockout I/R: *Pex11a* knockout heart subjected to I/R injury; sys: systolic pressure; dyas: diastolic pressure.

To investigate whether the observed developmental abnormalities caused by the *Pex11a* knockout impacted cardiac performance, the isolated hearts were subjected to ischemic stress using a Langendorff reperfusion system. The cardiac performance was determined by calculating the pressure difference between systole and diastole (ΔP_sys/dia_). To this purpose, hearts were isolated and attached to a Langendorff apparatus. Perfusion and pressure recording were initiated for a period of 5 min. Thereafter perfusion was terminated for a duration of 45 min to simulate ischemia. Subsequently, reperfusion and pressure recording were initiated and continued for 120 min (Figure 6A). Pre-ischemic values for the wild-type and the knockout hearts showed an average value of 91.7 ± 5.7 mmHg and 86.3 ± 6.7 mmHg, respectively. Pressure-monitoring during 120 min reperfusion showed that wild-type hearts recovered 83.9% and knockout hearts 45.7% of the pre-ischemic performance after 30 min of reperfusion (Figure 6B). At their maximum (4 min after reperfusion), the knockout hearts recovered 52% of the initial cardiac performance (Figure 6B).

Following I/R histopathological features in the LVs were investigated using HE-staining (Figure 6D,E). This revealed that in the myocardium of the *Pex11a* knockout mice, the occurrence of cardiomyocyte damage (shrunken, blebbed or ruptured cardiomyocytes and contraction band necrosis) was elevated (Figure 6D,E). Quantification revealed that in the *Pex11a* knockout heart, 42.2%, while in the wild-type heart only 18.9% of cardiomyocytes were noticeably damaged after I/R (Figure 6L). Additionally, following I/R-stress, the expression of the cardiac markers *Nppb* and *Mhc6* was significantly reduced in the *Pex11a* knockout LVs (Figure 6C).

Following I/R-stress, Cx43 is typically relocated to the longitudinal sides of the cardiomyocytes, a process termed “lateralization” [50,51,52]. After I/R-stress lateralization of Cx43 was detected in the hearts of both genotypes; however, it was slightly more prominent in the cardiomyocytes of the *Pex11a*-deficient mice (Figure 6F,G). An increase in the 8OHdG-staining was also observed in the I/R-subjected *Pex11a* knockout hearts (Figure 6H,I). Also, in the I/R-subjected hearts, 8OHdG displayed a mitochondrial localization as shown using double-labeling with the mitochondrial SOD2 (Supplemental Figure S7).

Western blots further indicated that following I/R, the abundance of catalase was elevated in the *Pex11a* knockout LVs, while the one for PEX19p was reduced, and the one for PEX14p was unchanged (Figure 6M,N).

## 4. Discussion

### 4.1. The Knockout of Pex11a Disturbs the Homeostasis of the Peroxisomal Compartment

The *Pex11a* general knockout mouse model [40] poses several advantages compared to other peroxisomal knockout models: (i) *Pex11a* knockout mice are viable after birth, (ii) have a normal life span (2–3 years), and (iii) display a “mild” phenotype [40]. For these reasons, this model is ideal for the investigation of the long-term health risks posed by “mild” peroxisomal dysfunction in different tissues, including the cardiac muscle. In contrast to other peroxisomal biogenesis disorder (PBD) mouse models (such as the *Pex5*, *Pex2*, *Pex13* knockout mice), which mimic the Zellweger spectrum symptoms [53,54,55], the *Pex11a* knockout mice still contain peroxisomes that harbor the typical peroxisomal marker enzymes [40]. In the *Pex5* mouse knockout model, ultra-structural defects in the heart’s mitochondria have been documented. However, it is impossible to investigate the consequences of these defects in adult mice, as the pups are either non-viable at birth or perish shortly thereafter [12,56]. In the Zellweger spectrum disorder, Refsum’s disease, cardiac defects, including dilated cardiomyopathy and arrhythmias, were occasionally described. Currently, two different mouse models exist that mimic this peroxisomal defect, namely the PEX7 and phytanoyl-CoA hydroxylase knockout mice. Surprisingly, both do not display evident alteration in cardiac structure [57,58].

The present study demonstrated that the knockout of *Pex11a* increased peroxisome volume and abundance in cardiomyocytes. In contrast to cardiac tissue, no discernible change was reported in the number or morphology of peroxisomes in hepatocytes of the same mouse model during electron microscopic detection of catalase activity [40]. Using a different PEX11a knockout mouse model, a slightly reduced number of peroxisomes in the liver and in the proximal tubules of the kidney was observed [59]. Experiments conducted in yeast or in the human parasite *Trypanosoma brucei* demonstrated that the depletion of the corresponding *Pex11*-gene homologues reduced the number but increased the size of peroxisomes [39,60,61,62,63,64,65]. In consideration of the differential transcriptional regulation of the *Pex11* genes [66], their distinct expression in the left and right ventricles [3], and the differences in peroxisomal alterations induced by their depletion in various cell types, it is likely that the PEX11 proteins function in an isoform- as well as cell-type-specific manner.

PEX11 proteins are responsible for peroxisomal biogenesis in cooperation with proteins from the mitochondrial fission and autophagy machinery [25,37,38,67,68,69,70,71]. The observed increase in peroxisomal size may therefore be a consequence of reduced fission events, potentially due to the lowered expression of FIS1 and MFF1. As demonstrated in earlier studies, the suppression of FIS1 expression in COS-7 cells resulted in the enlargement of peroxisomes [69]. In view of the absence of any readily discernible alterations in mitochondrial morphology and given that we only conducted qPCR analyses with low n on this aspect, further investigation of fission- and pexophagy-related proteins and their interaction with PEX11a is required to assess the hypothesis that the increased number of enlarged peroxisomes is caused by a defect in peroxisomal fission and/or degradation.

### 4.2. In the Pex11a Knockout Mice, an Increase in Mitochondrial Oxidative Stress Has Been Indicated

A large amount of ROS originates from mitochondria due to the leak of electrons from the electron transport chain (ETC) (mainly complexes I and III) and through the activity of SODs. Elevated activity of the ETC upon overexpression of complexes I and III has been previously associated with increased abundance of ROS [10].

The altered abundance of ETC complexes we observed in the *Pex11a* knockout might impact ETC function and so increase mitochondrial ROS. Our previous proteomic analysis showed that 12 out of the 29 differentially abundant proteins detected in the myocardium of the *Pex11a* knockout mouse were mitochondrial [72]. These comprised elevated abundance of subunits of ETC complexes II and III and reduced abundance of the ATPase inhibitor Atp5if1 and of glutathione peroxidase 1 (GPX1) [72]. These results were not unexpected, as the occurrence of ETC dysfunction as well as mitochondrial depolarization has been previously associated with peroxisomal deficiencies [12,13,14]. However, the electron micrographs did not reveal an alteration in mitochondrial ultrastructure. To define the existence and the extend of a mitochondrial defect in the cardiomyocytes of the *Pex11a* knockout mouse, functional analyses including the determination of the mitochondrial membrane potential and of the ETC-activity will be required in the future.

Also peroxisomes significantly contribute to the production of ROS by generating H_2_O_2_ and superoxide anions, through the activity of several oxidases [73,74,75,76]. Excess H_2_O_2_ is usually scavenged in various subcellular compartments by antioxidative enzyme-systems such as glutathione peroxidases (GPX) and peroxiredoxins (PRX) [76]. In addition to containing different isoforms of these enzymes, peroxisomes retain an outstanding role in the defense against H_2_O_2_ because they contain catalase, which has the highest turnover number for H_2_O_2_ [77] and has been suggested to be cardio-protective [34,78,79,80,81,82,83]. In the heart, oxidative stress induces degradation and conformational changes to the proteins of the contractile apparatus. Indeed, experiments demonstrated that H_2_O_2_ altered the ultrastructure of actin and tropomyosin, leading to contractile dysfunction [75,84,85,86,87].

Next to H_2_O_2_ also NOS-uncoupling is a possible source of excess ROS. During NOS-uncoupling, increased arginase activity inhibits nitric oxide (NO) synthesis, lowering NO production and increasing other ROS [88,89]. It is therefore notable that a very significant upregulation of the expression of arginase was found in the LV of the *Pex11a* knockout mice. Elevated arginase expression, as observed in the *Pex11a* knockout, and higher circulating serum arginase levels exacerbate cardiovascular diseases and cardiac remodeling. The pathogenetic factors included endothelial cell senescence and inflammation, fibroblast proliferation, and elevated ROS [88,89,90]. In the future, it will be of interest to examine whether the elevation of the arginase mRNA abundance is accompanied by an increase in its protein abundance and its activity. It will also be of interest to establish how the removal of PEX11a regulates the availability of arginase, either by direct or indirect effect.

### 4.3. The Knockout of Pex11a Alters the Lipid Composition of the Myocardium

MALDI-MS imaging demonstrated that medium- and long-chain hydroxylated CARs were increased in the cardiac tissue of the *Pex11a* knockout. Hydroxylated CARs are derivatives of 3-hydroxyacyl-CoAs produced by the enoyl-CoA-hydratase during the β-oxidation of fatty acids. In mitochondria 3-hydroxyacyl-CoAs can be converted to acylcarnitine by CPT-II, resulting in the synthesis of 3-hydroxy acylcarnitines [91].

Previous publications described the accumulation of hydroxylated long-chain acylcarnitines in the diabetic myocardium and in the plasma of patients affected by mitochondrial cardiomyopathy [92,93]. Vissing et al. further reported that in patients, the rise in hydroxylated long-chain acylcarnitines was associated with reduced activity of the respiratory chain [93]. These results suggest that the accumulation of CAR-OHs has the potential to disrupt mitochondrial function, a phenomenon that may also occur in the cardiomyocytes of the *Pex11a* knockout heart.

In contrast to hydroxycarnitines, the abundance of CARs was consistently reduced in the *Pex11a* knockout heart. This was surprising since the accumulation of CARs (i) is known to induce lipotoxicity in cardiomyocytes, (ii) is a hallmark of myocardial ischemia and (iii) induces the loss of myocardial regenerative capability [1,94,95]. In future studies, the measurement of the activity of the mitochondrial and peroxisomal fatty acid oxidation will provide valuable insights into the underlying mechanisms that underpin the observed decrease in acylcarnitine levels in the *Pex11a* knockout model.

Transcription factors of the PPAR family are directly involved in the regulation of the peroxisomal metabolism (e.g., expression of β-oxidation-related genes) and biogenesis [14]. They are also critical for cardiac energy metabolism because they regulate the abundance of proteins and enzymes of the metabolism of cardiomyocytes. For this reason, agonists that modulate the activity of PPARa and PPARg have been considered for the treatment of cardiac diseases [15,36]. There have been speculations that physiological PPAR regulators are lipid derivatives, some of which are intermediates of the peroxisomal metabolism [3].

Son et al. describe that the MHC-promotor-driven overexpression of PPARg in mice resulted in the accumulation of lipids in the heart, which could be reversed by the knockout of PPARa [96]. Furthermore, they observed a reduction in CAR levels, an increase in LD and TG content, and elevated expression of lipid metabolism-related enzymes [96]. Despite requiring further validation through Western blot analyses and increased n-value, it is interesting to note that the qPCR-results presented here indicate a significant reduction in PPARa- and a slight, not significant, increase in PPARg transcripts.

In accordance with the findings of Son et al. [96], the cardiomyocytes of the *Pex11a* knockout mouse exhibited a lower abundance of CARs and a higher number of LDs. However, instead of increased TG abundance, their abundance was reduced in *Pex11a* knockout hearts. Lipidomic analysis of the same PEX11a knockout hearts also indicated lowered CARs and TG content [72]. This discrepancy might be explained by assuming that LDs do not only contain TGs but also other lipids, and that their abundance is altered in the *Pex11a* knockout cardiomyocytes. Indeed, studies reported the presence of other lipids in the core and the monolayer of LDs, including acylceramides, hexosylceramides, diglycerides, fatty acid esters of hydroxy fatty acids, lysophosphatidylcholine, phosphatidylcholine and phosphatidylethanolamine [97,98]. These lipids correspond well to those elevated in the PEX11a knockout heart and might explain the increased size of the LDs [72]. Interestingly a study on hepatocytes infected with hepatitis C reported larger LDs containing higher amounts of ceramide and hexosylceramide but not of triglycerides [99].

### 4.4. Pex11a Knockout Mice Exhibit Increased Sarcomere Length and Display Reduced Recovery and Increased Cellular Damage Following Ischemia–Reperfusion

Although the hearts of the adult *Pex11a* knockout mice show no obvious macroscopic changes, the unphysiological elongation of the sarcomeres that we observed might render the cardiomyocytes more fragile and more prone to damage, leading to stronger I/R injury. Furthermore, the abundance of very long sarcomeres may increase with age and the accumulation of ROS.

Despite not being entirely physiological equivalent to an in vivo model, the use of Langendorff reperfusion on isolated *Pex11a* knockout mice hearts proved to be a valuable instrument for deriving a functional perspective on the potential consequences of a peroxisomal defect on cardiac performance. The evidence provided in this manuscript suggested that the knockout of *Pex11a* reduced the heart’s pressure output following I/R, indicating diminished cardiac recovery (ex vivo). Upon examination of the heart tissue of the *Pex11a* knockout mice following I/R, a greater degree of cellular damage and contraction band necrosis was observed when compared to wild-type tissue. The necrosis of the contraction band is a typical feature of myocardial infarction and sudden death occurring in atherosclerotic coronary artery disease [100,101]. Another noteworthy observation was the lateralization of the gap junction component Cx43 in the cardiomyocytes of the *Pex11a* knockout following I/R. Several cardiac pathologies, including myocardial infarction, have been associated with the sequestration of Cx43 to the lateral side of the cardiomyocytes, accompanied by a concomitant alteration of the electrical conduction at the gap junctions and the development of arrhythmias [51,52,102,103,104,105].

A previous publication demonstrated a connection between the overexpression of PLIN2 in cardiomyocytes, steatosis and the remodeling of Cx43 [106]. As one possible cause for the rearrangement of Cx43 at the intercalated disk, the authors mention that the LDs might interfere with microtubule-mediated trafficking of Cx43 [106]. Other studies instead showed that hearts perfused with H_2_O_2_, and those affected by postoperative atrial fibrillation, exhibited increased oxidative stress and lateralization of Cx43 [107,108].

We therefore postulate that the increased abundance of PLIN2, the accumulation of LDs and the elevated ROS-formation of the *Pex11a* cardiomyocytes might trigger Cx43 remodeling and lateralization following I/R.

### 4.5. Conclusions

Despite the extensive research conducted into cardiomyocyte metabolism and its regulation, there is a scarcity of knowledge regarding peroxisomal function in these cells. This lack of knowledge is especially problematic in the context of implementing pharmacological interventions involving PPAR agonists to compensate for cardiac defects.

The results presented in this study indicate that the suboptimal recovery of the *Pex11a* knockout heart might be attributed to the heightened level of post-ischemic cardiomyocyte damage caused by morphological and metabolic aberrations that weakened the cardiac muscle and consequently heightened its susceptibility to ischemia. This suggests that the peroxisomal compartment has a potential cardioprotective effect during conditions of myocardial stress, thereby playing a role in mitigating ischemia–reperfusion injury.

## Data Availability

Data is contained within the article or Supplementary Materials. Additional datasets are available on request from the authors.

## Supplementary Materials

The following supporting information can be downloaded at: https://www.mdpi.com/article/10.3390/cells15010012/s1.
