# Supplementary material for: The Knockout of PEX11a Results in Mild Peroxisomal Dysfunction and Lowered Cardiac Recovery Following Langendorff-Mediated Ischemia–Reperfusion in Mice
Source: Cells. 2025 Dec 20;15(1):12. doi: 10.3390/cells15010012 (PMC12786121; doi:10.3390/cells15010012)

Supplemental Figure originals

The knockout of PEX11a results in mild peroxisomal dysfunction and impaired cardiac recovery following Langendorff-mediated ischemia-reperfusion in mice

Claudia Colasante[1*], Jiangping Chen[2], Vannuruswamy Garikapati[3], Bernhard Spengler[4], Klaus-Dieter Schlüter[5], Eveline Baumgart-Vogt[6*]

[1] Institute for Anatomy and Cell Biology, Justus Liebig University, Aulweg 123, 35392 Giessen, Germany; claudia.colasante@anatomie.med.uni-giessen.de

[2] Institute for Anatomy and Cell Biology, Justus Liebig University, Aulweg 123, 35392 Giessen, Germany; UKGM Giessen, Medical Clinic and Polyclinic II, Klinikstraße 33, 35392 Gießen, Germany; jiangping.chen@anatomie.med.uni-giessen.de

[3] Institute for Anatomy and Cell Biology, Justus Liebig University, Aulweg 123, 35392 Giessen, Germany; Max Planck Institute of Molecular Cell Biology and Genetics, Pfotenhauerstrasse 108, 01307 Dresden, Germany; garikapa@mpi-cbg.de

[4] Institute of Inorganic and Analytical Chemistry, Justus Liebig University, Heinrich-Buff-Ring 17, 35392 Giessen, Germany; bernhard.spengler@anorg.chemie.uni-giessen.de

[5] Institute for Physiology, Justus Liebig University, Aulweg 129, 35392 Giessen, Germany. klaus-dieter.schlueter@physiologie.med.uni-giessen.de

[6] Institute for Anatomy and Cell Biology, Justus Liebig University, Aulweg 123, 35392 Giessen, Germany; eveline.baumgart-vogt@anatomie.med.uni-giessen.de

* Correspondence: C.C.: claudia.colasante@anatomie.med.uni-giessen.de; E.B.V.: eveline.baumgart-vogt@anatomie.med.uni-giessen.de

FIGURE 2

A

WT 35 w

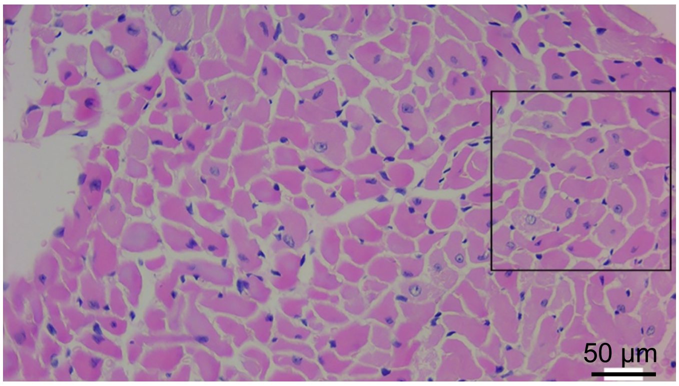

KO 35 w

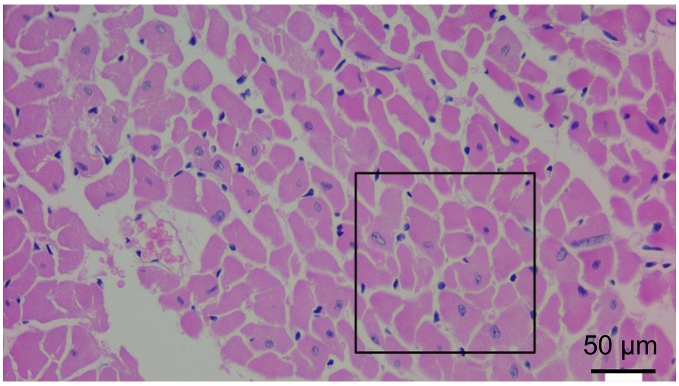

WT 65 w

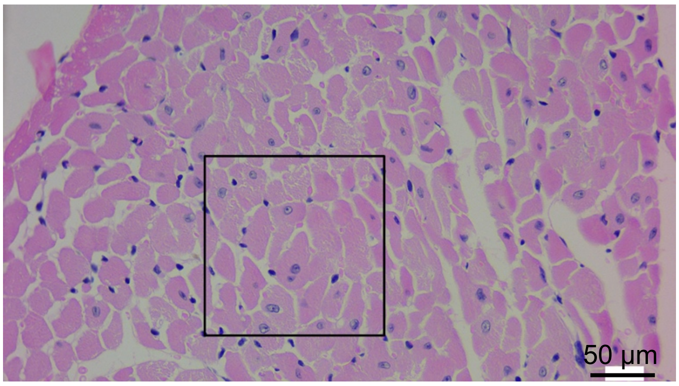

KO 65 w

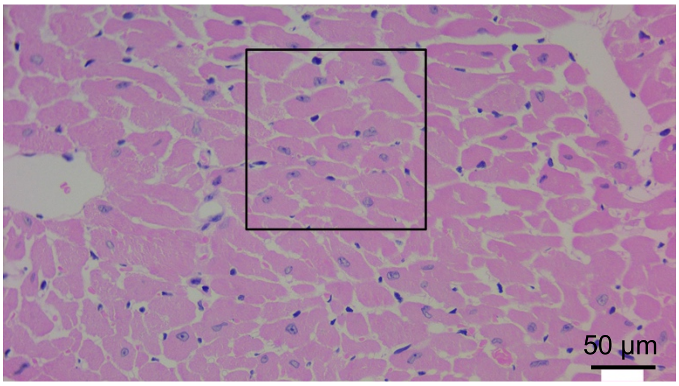

FIGURE 2

B

WT 35 w

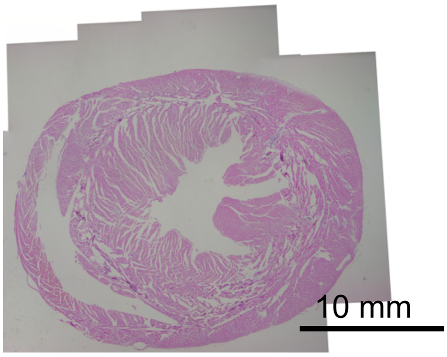

KO 35 w

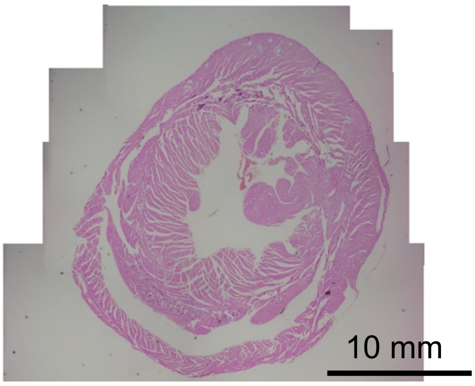

WT 65 w

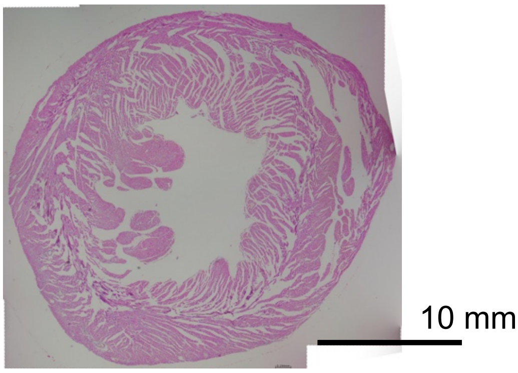

KO 65 w

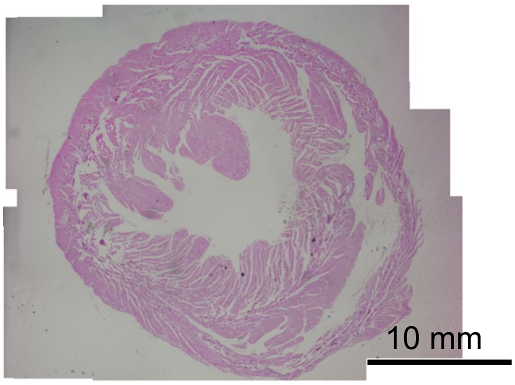

FIGURE 2

E

WT 35 w

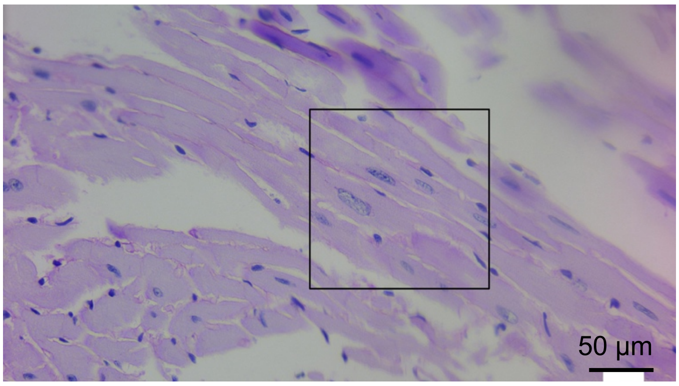

KO 35 w

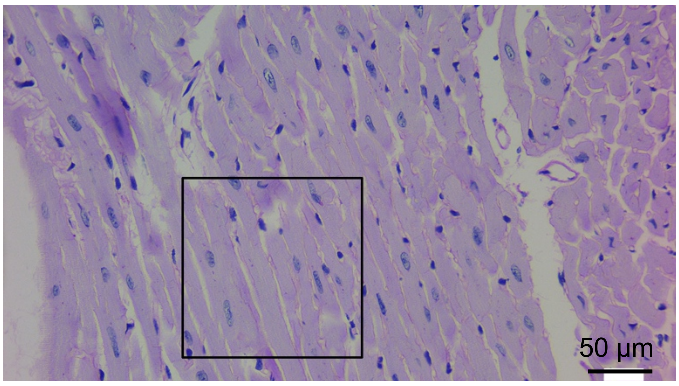

WT 65 w

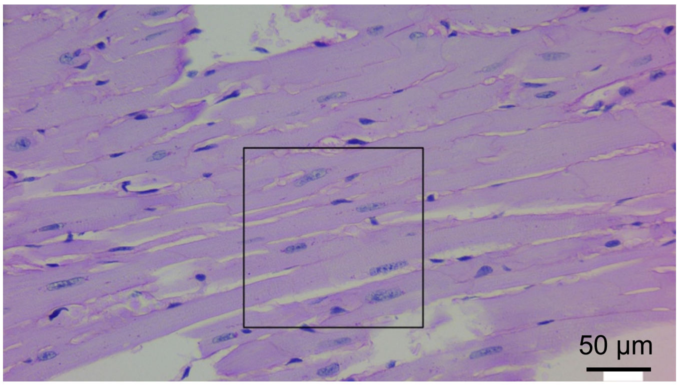

KO 65 w

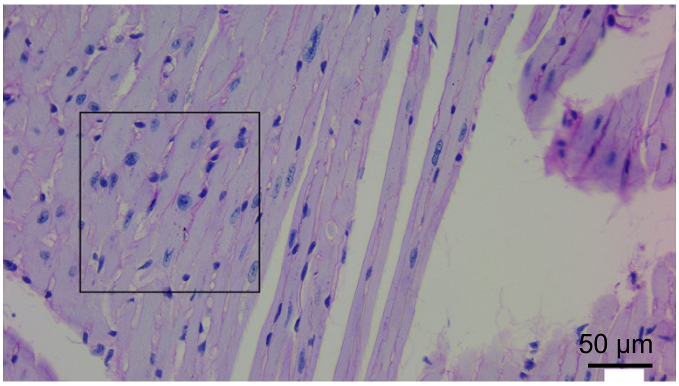

FIGURE 2

F

WT 35 w

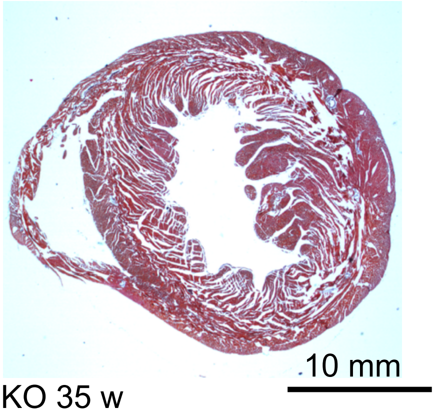

KO 35 w

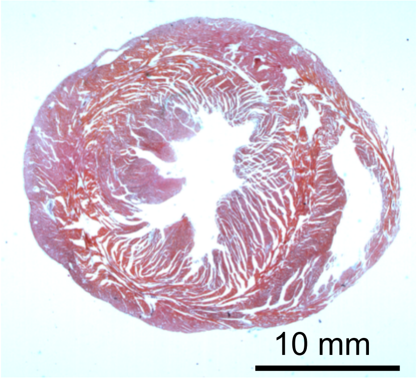

WT 65 w

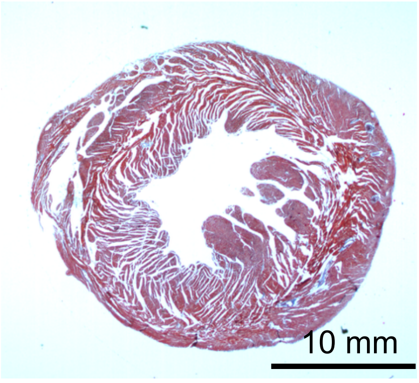

KO 65 w

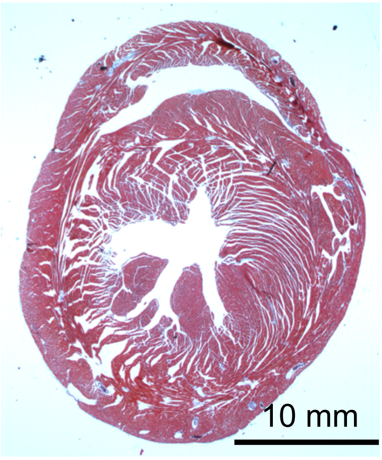

FIGURE 2

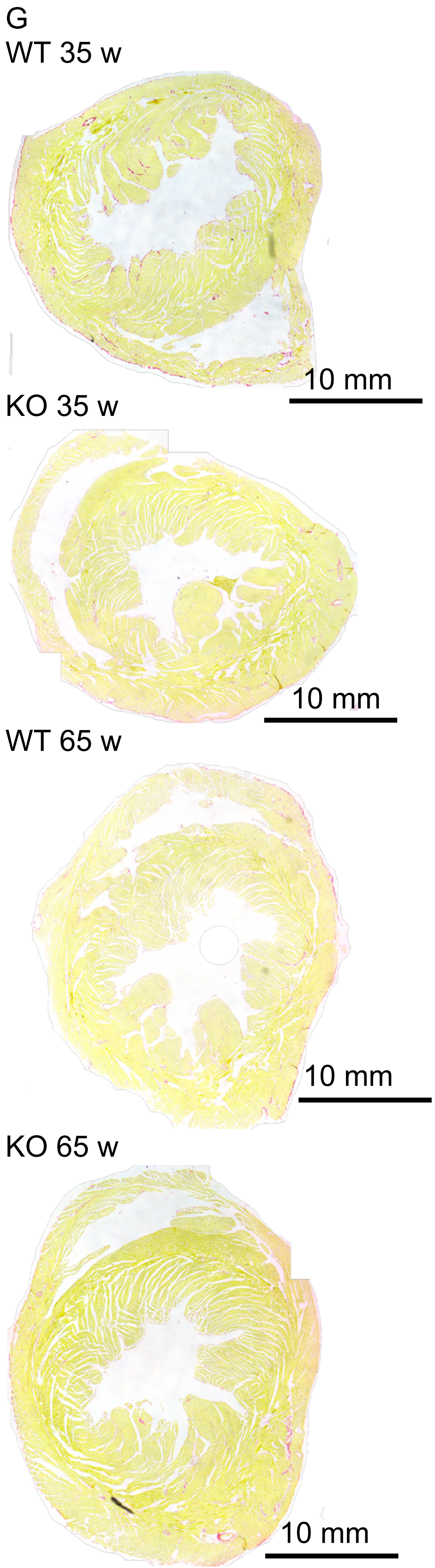

FIGURE 3

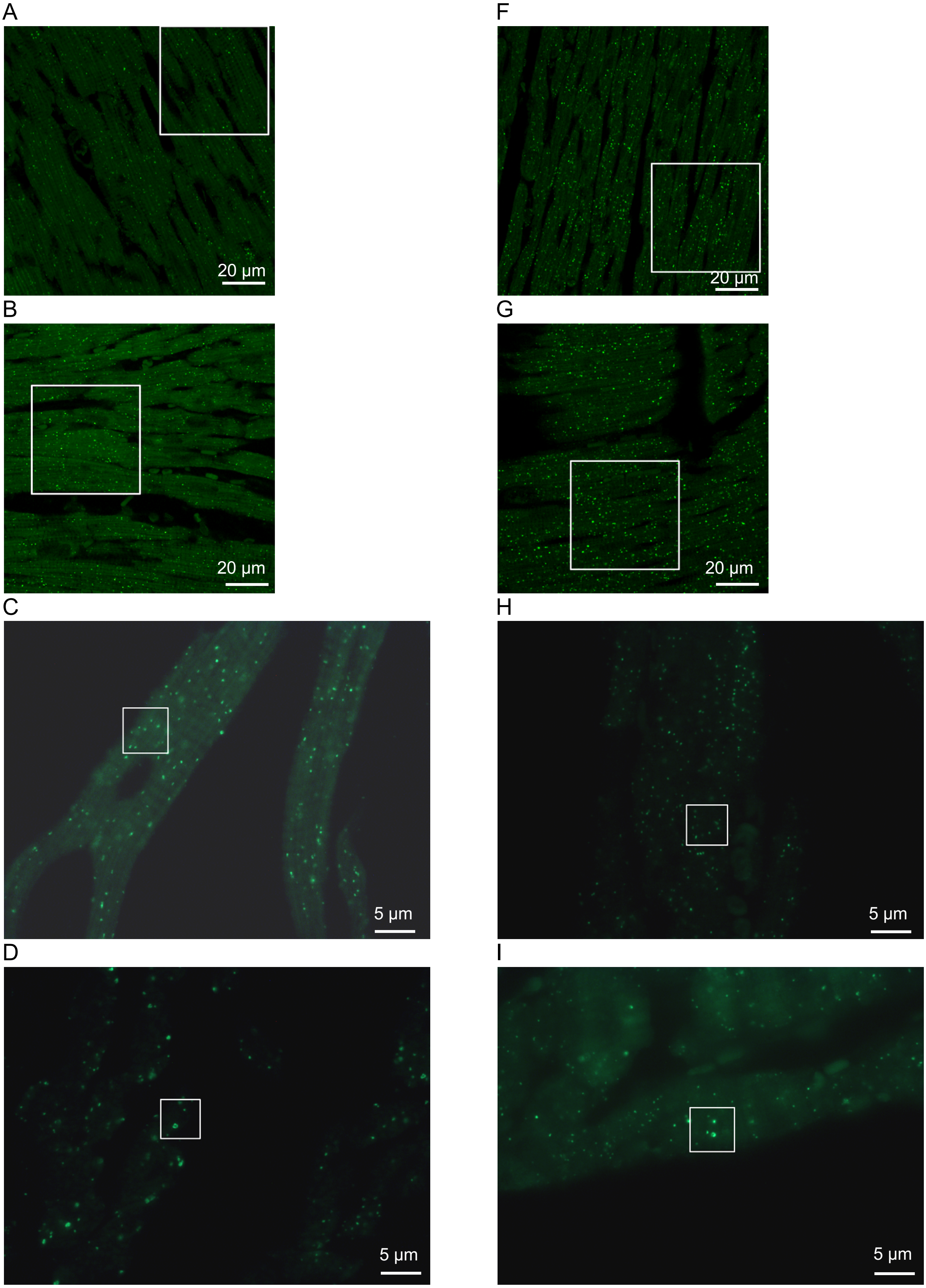

FIGURE 3

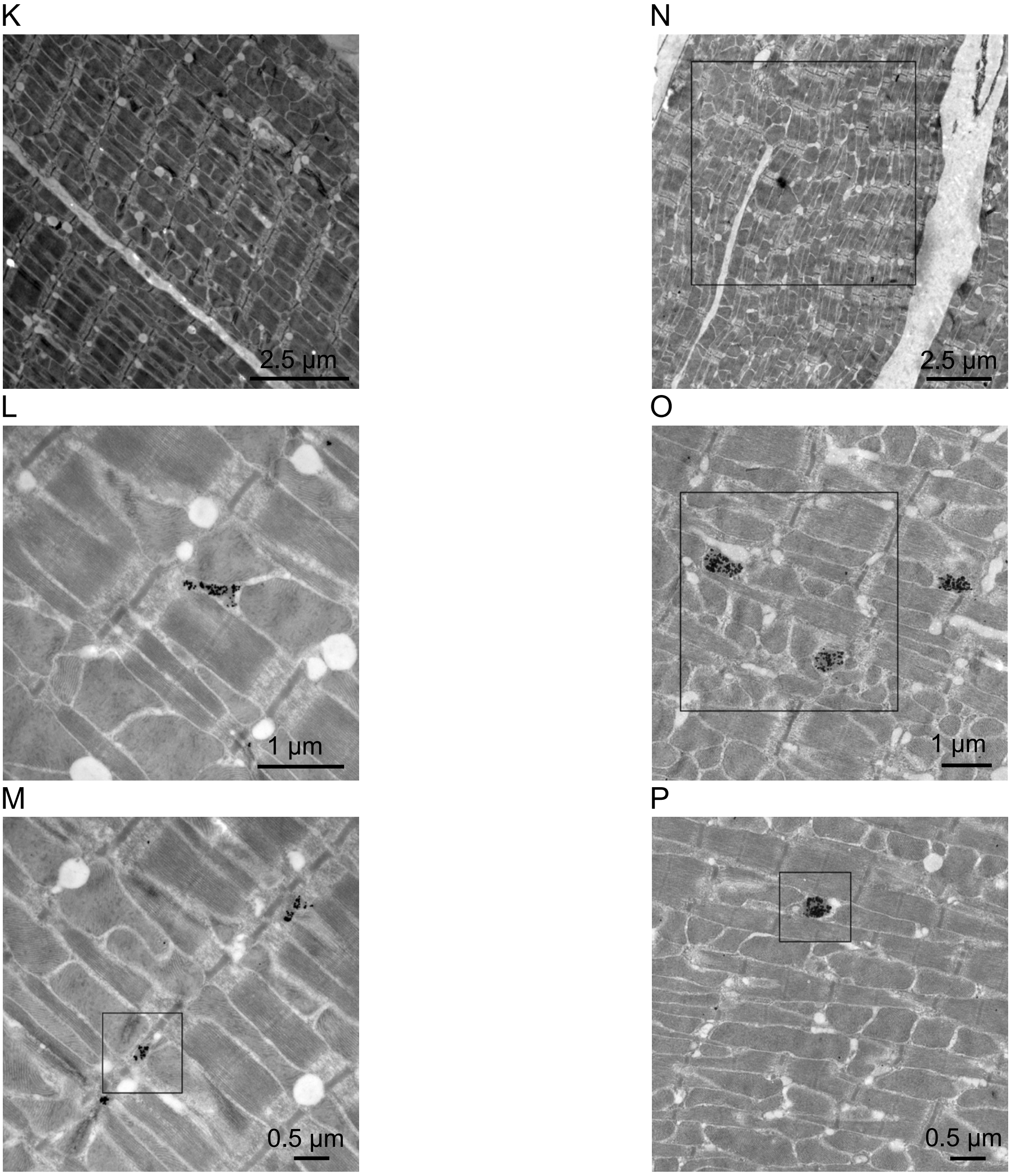

FIGURE 4

A

WT

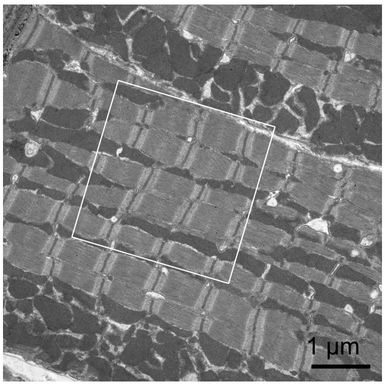

KO

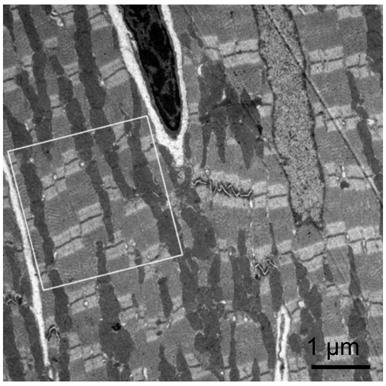

C

WT

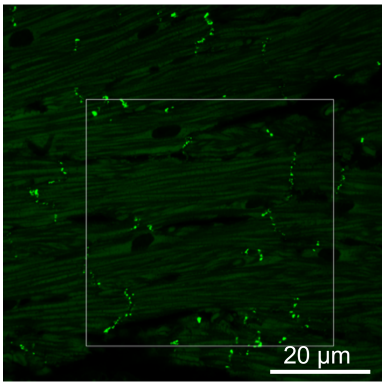

KO

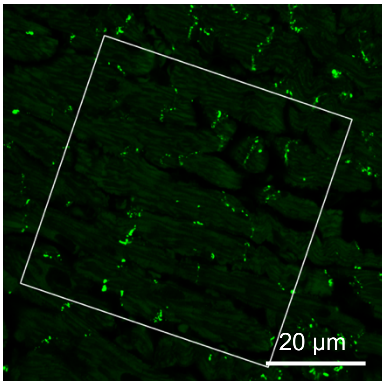

E

WT

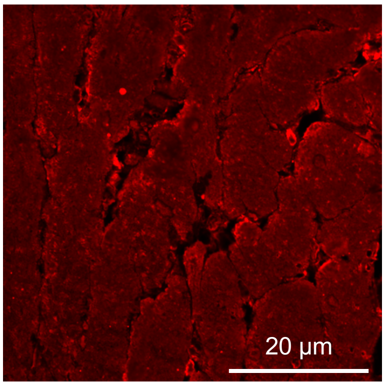

KO

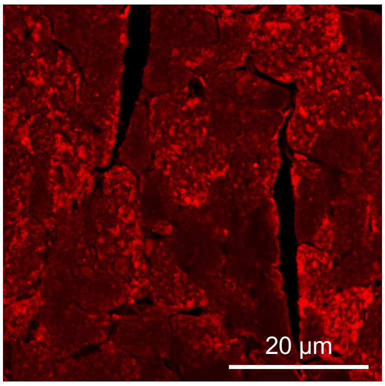

FIGURE 5

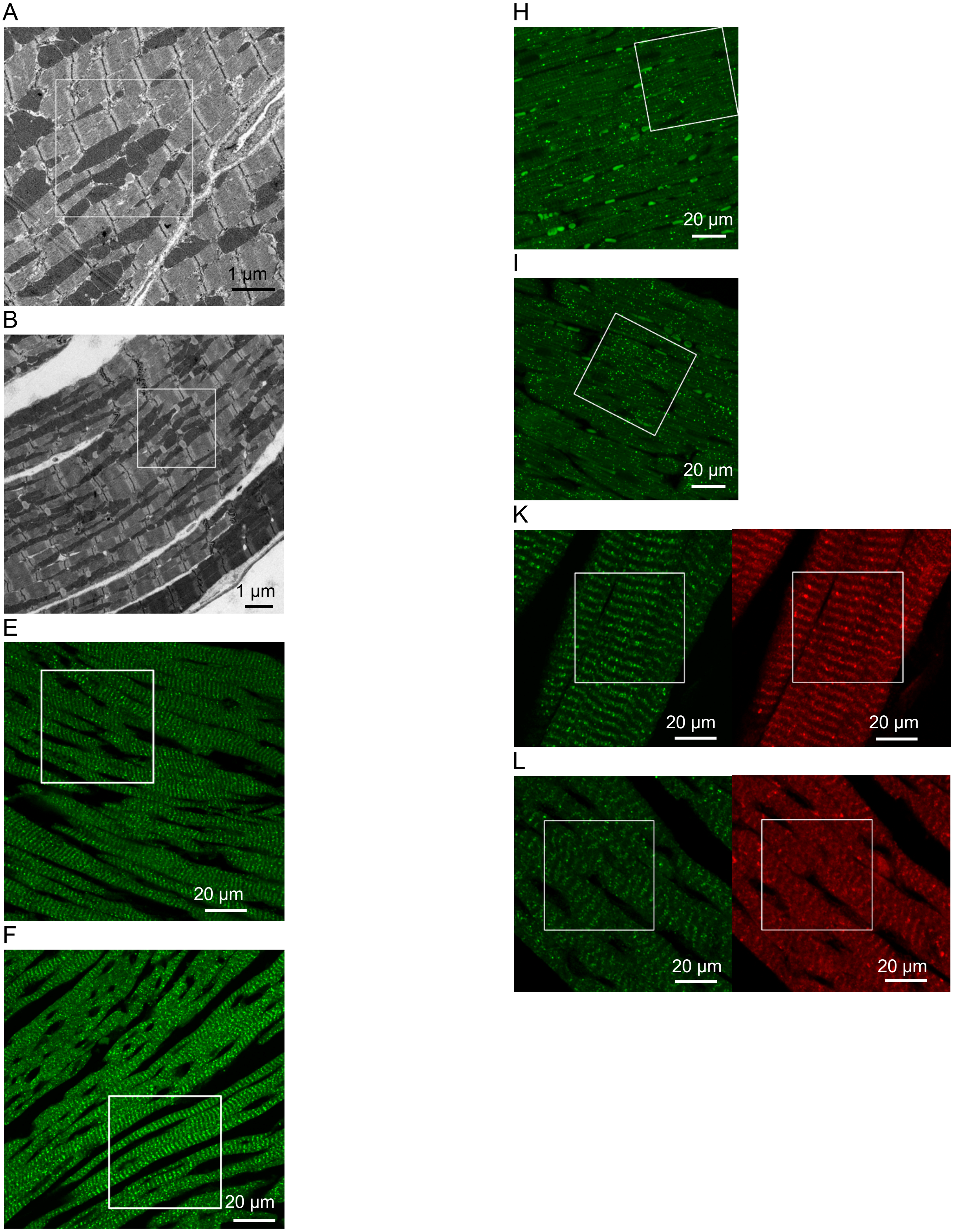

FIGURE 6

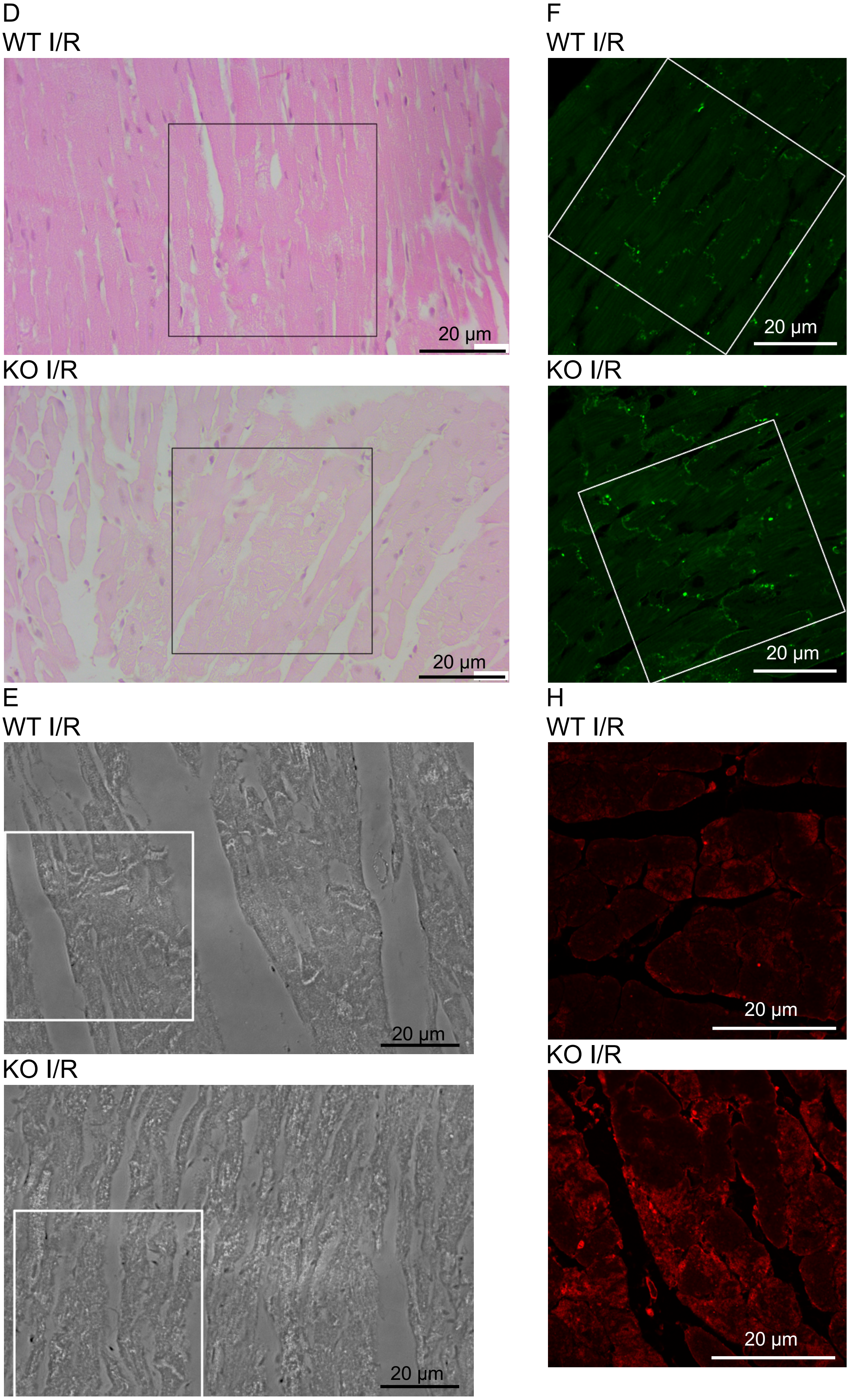
D
WT I/R
20 µm
KO I/R
20 µm
F
WT I/R
20 µm
KO I/R
20 µm
E
WT I/R
20 µm
KO I/R
20 µm
H
WT I/R
20 µm
KO I/R
20 µm

FIGURE 6

J

WT I/R

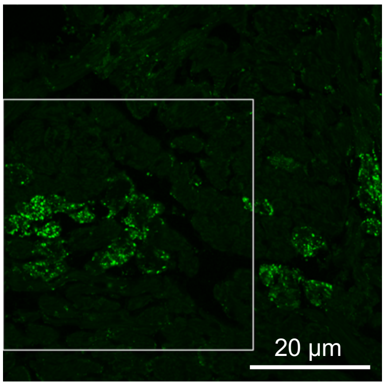

KO I/R

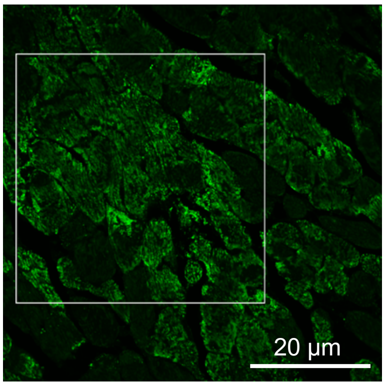

FIGURE S3

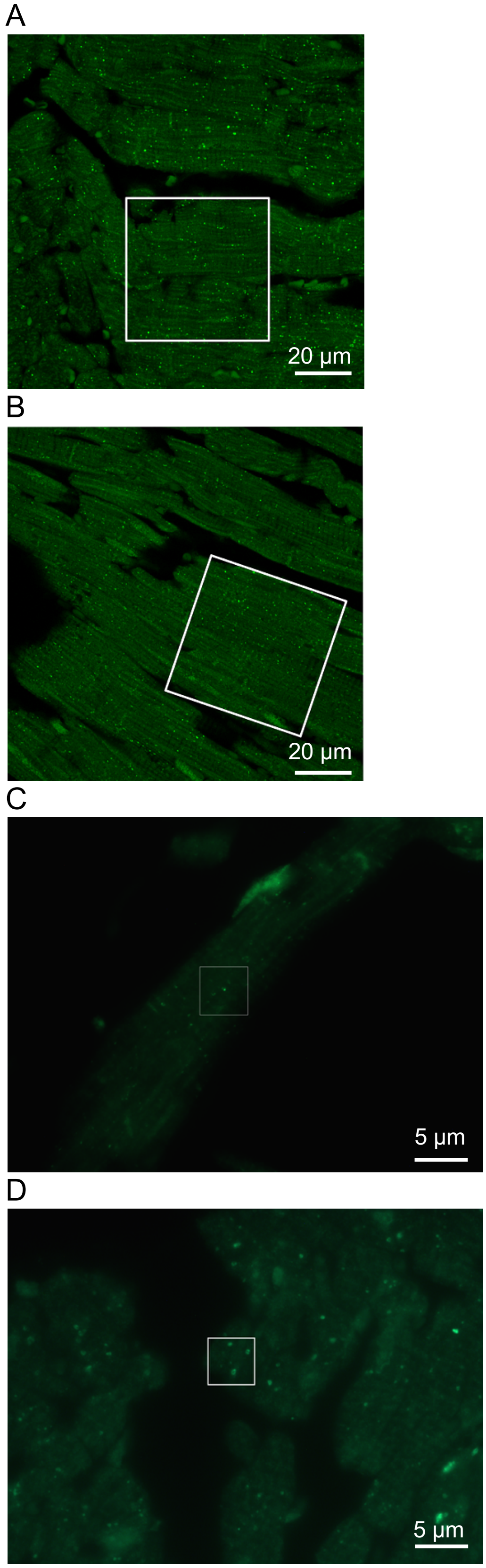

FIGURE S4

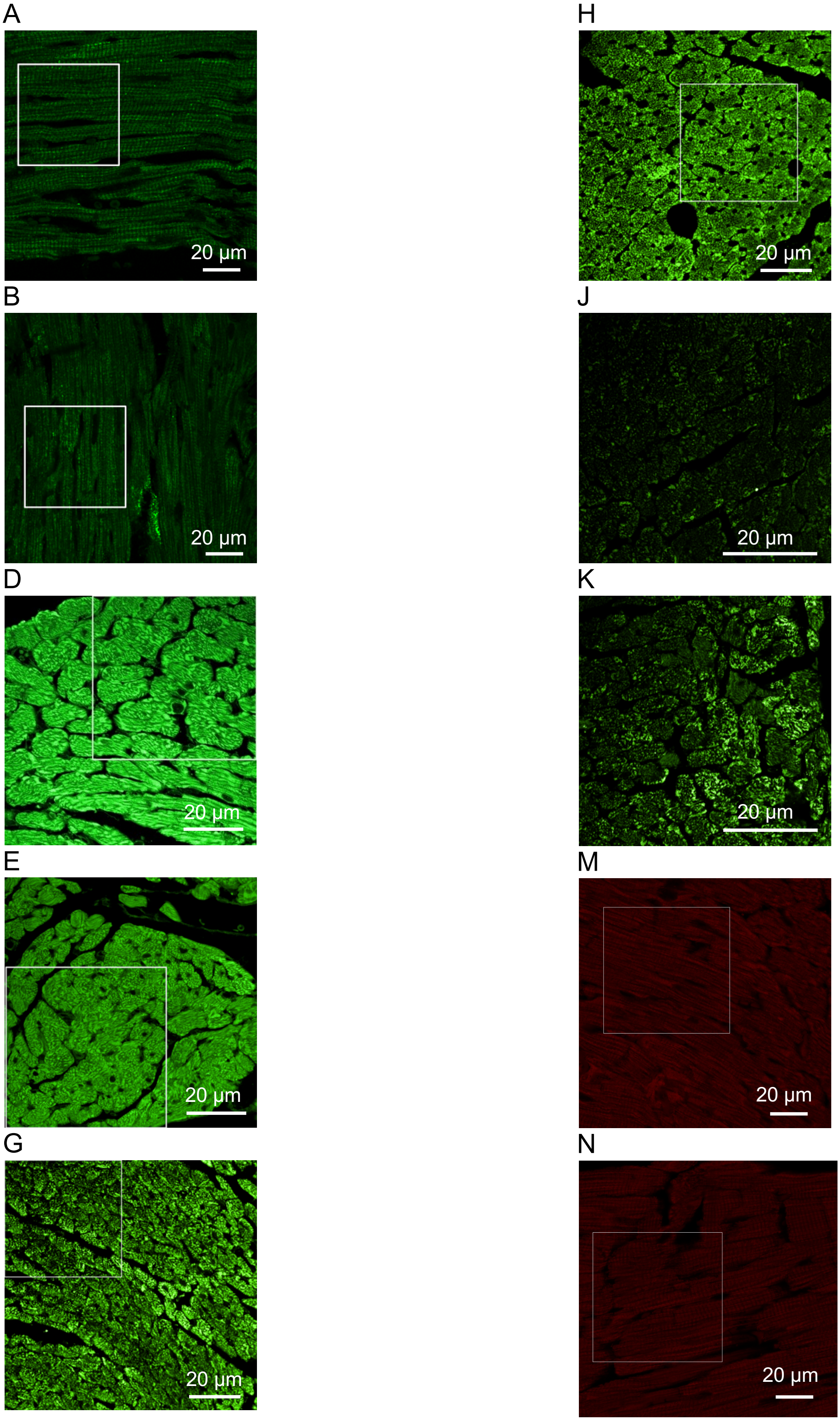
A
20 µm
H
20 µm
B
20 µm
J
20 µm
D
20 µm
K
20 µm
E
20 µm
M
20 µm
G
20 µm
N
20 µm

FIGURE S7

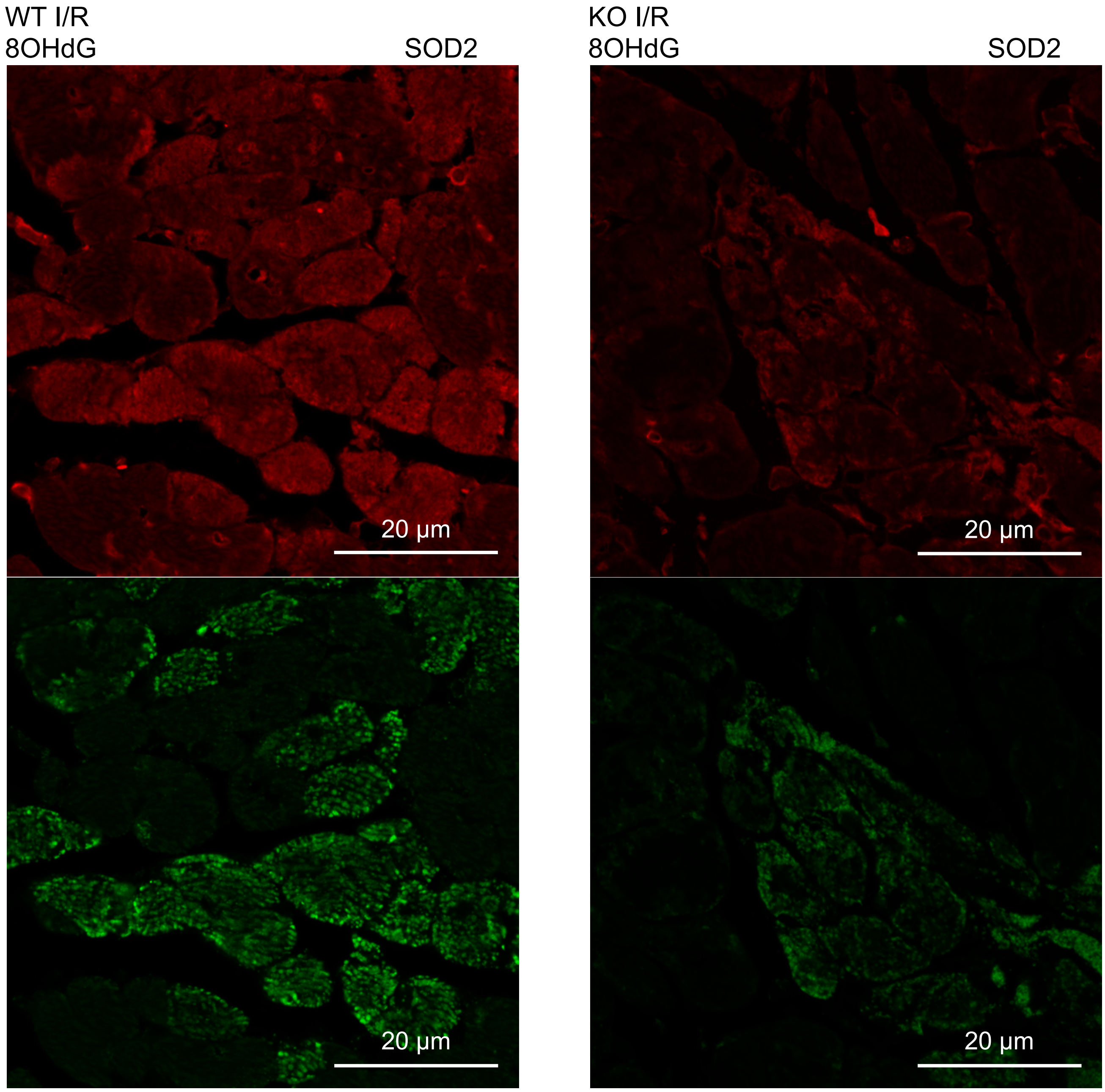

Supplement: Supplementary file 1 [file cells-15-00012-s001.zip › Original Images_/Supplemental Original Images.pdf]
