# Supplementary material for: The Knockout of PEX11a Results in Mild Peroxisomal Dysfunction and Lowered Cardiac Recovery Following Langendorff-Mediated Ischemia–Reperfusion in Mice
Source: Cells. 2025 Dec 20;15(1):12. doi: 10.3390/cells15010012 (PMC12786121; doi:10.3390/cells15010012)

Supplemental western blots

The knockout of PEX11a results in mild peroxisomal dysfunction and impaired cardiac recovery following Langendorff-mediated ischemia-reperfusion in mice

Claudia Colasante[1*], Jiangping Chen[2], Vannuruswamy Garikapati[3], Bernhard Spengler[4], Klaus-Dieter Schlüter[5], Eveline Baumgart-Vogt[6*]

[1] Institute for Anatomy and Cell Biology, Justus Liebig University, Aulweg 123, 35392 Giessen, Germany; claudia.colasante@anatomie.med.uni-giessen.de

[2] Institute for Anatomy and Cell Biology, Justus Liebig University, Aulweg 123, 35392 Giessen, Germany; UKGM Giessen, Medical Clinic and Polyclinic II, Klinikstraße 33, 35392 Gießen, Germany; jiangping.chen@anatomie.med.uni-giessen.de

[3] Institute for Anatomy and Cell Biology, Justus Liebig University, Aulweg 123, 35392 Giessen, Germany; Max Planck Institute of Molecular Cell Biology and Genetics, Pfotenhauerstrasse 108, 01307 Dresden, Germany; garikapa@mpi-cbg.de

[4] Institute of Inorganic and Analytical Chemistry, Justus Liebig University, Heinrich-Buff-Ring 17, 35392 Giessen, Germany; bernhard.spengler@anorg.chemie.uni-giessen.de

[5] Institute for Physiology, Justus Liebig University, Aulweg 129, 35392 Giessen, Germany. klaus-dieter.schlueter@physiologie.med.uni-giessen.de

[6] Institute for Anatomy and Cell Biology, Justus Liebig University, Aulweg 123, 35392 Giessen, Germany; eveline.baumgart-vogt@anatomie.med.uni-giessen.de

* Correspondence: C.C.: claudia.colasante@anatomie.med.uni-giessen.de; E.B.V.: eveline.baumgart-vogt@anatomie.med.uni-giessen.de

Supplemental material "Western blots"

Original western blots and corresponding Coomassie brilliant blue loading control.

The corresponding manuscript Figure and the detected protein are indicated above the western blots. The genotype and animal number are indicated above each lane. The arrows indicate the band of the correct molecular weight for each detected protein. Asterisks (*) denote the selected lanes illustrated in the manuscript´s Figures. Black boxes indicate lanes that were used for densitometry analysis. Densitometry was performed utilising the ImageJ 1.53o software. The values for the individual bands were normalized against the Coomassie brilliant blue staining, after which they were subjected to statistical analysis using the unpaired two-tailed Student's t-test using the software GraphPad Prism 9.5.0 (GraphPad Software, Boston, MA). Only bands results from one western blot were directly compared to each other. The number of animals used and the number of experimental replicates is indicated in the figure legends of the manuscript. Lanes that displayed low quality of loading or unclear bands of the expected size were excluded from the quantification. These lanes are not boxed and are crossed out in the loading control. Abbr.: WT: wild-type Pex11a mice; KO: Pex11a knockout mice.

FIGURE 3

CATALASE

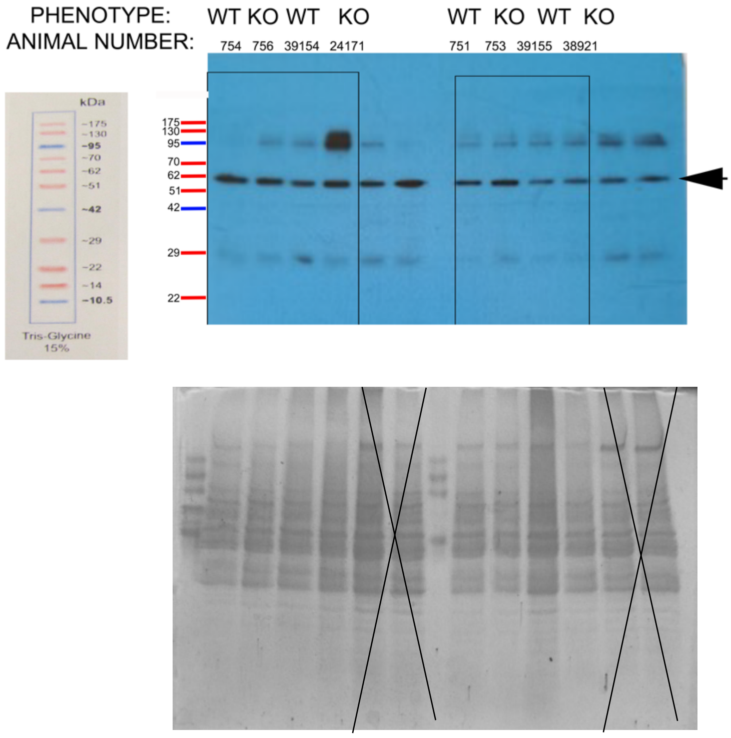

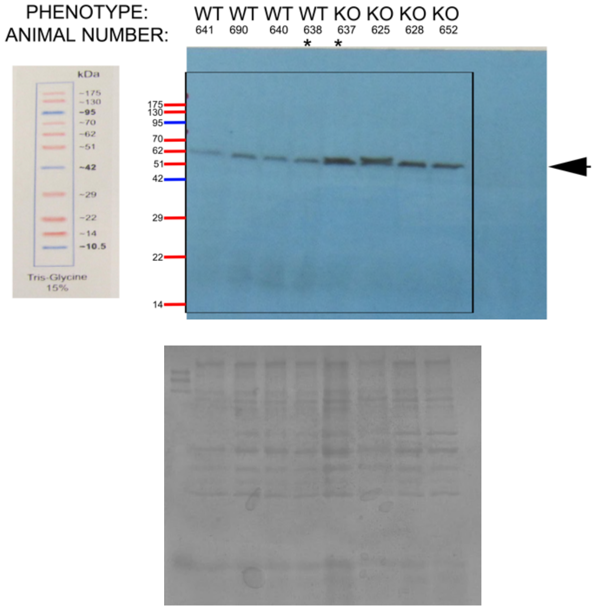

FIGURE 3

PEX14

PHENOTYPE: WT WT WT WT KO KO KO KO
ANIMAL NUMBER: 641 690 640 638 637 625 628 652

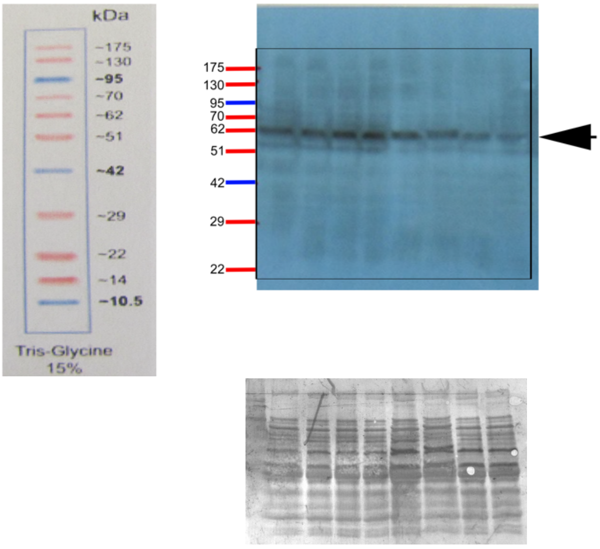

PHENOTYPE: WT KO WT KO
ANIMAL NUMBER: 751 753 39155 38921

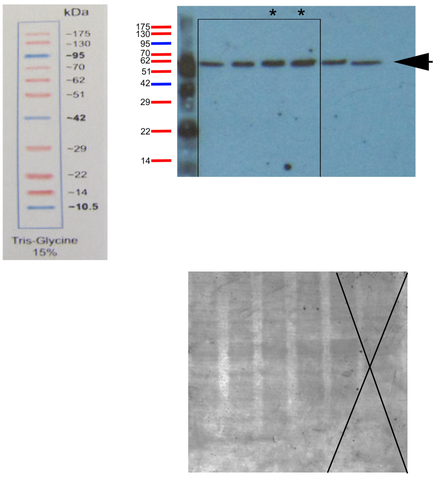

FIGURE 3

PEX19

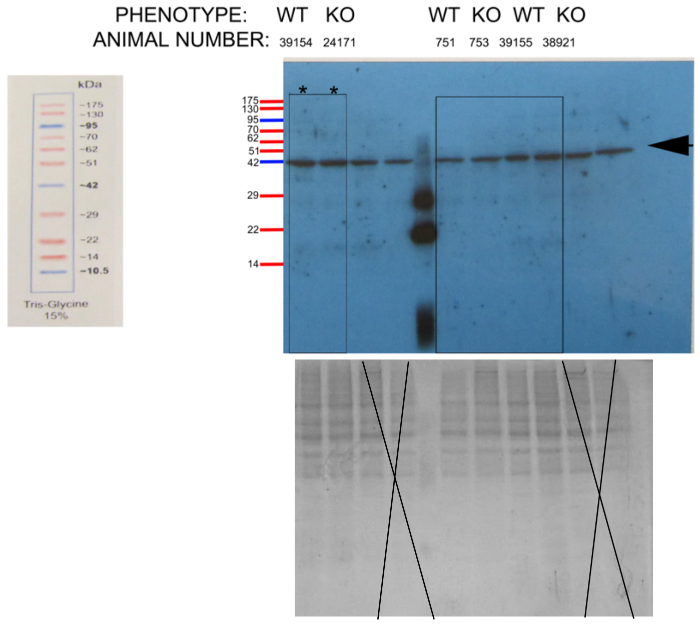

FIGURE 5

ABCD3

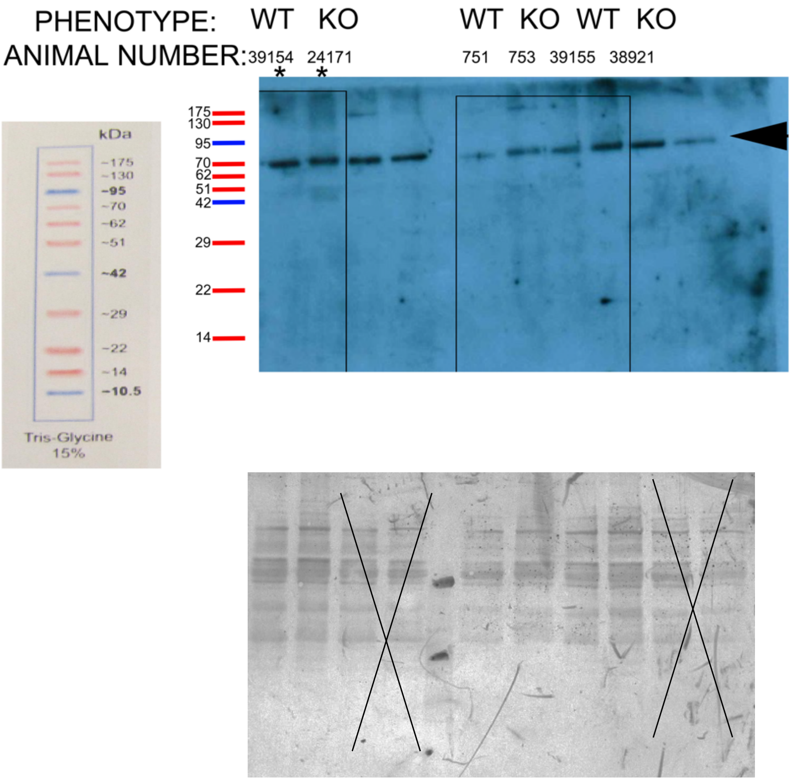

FIGURE 5

ACOX1

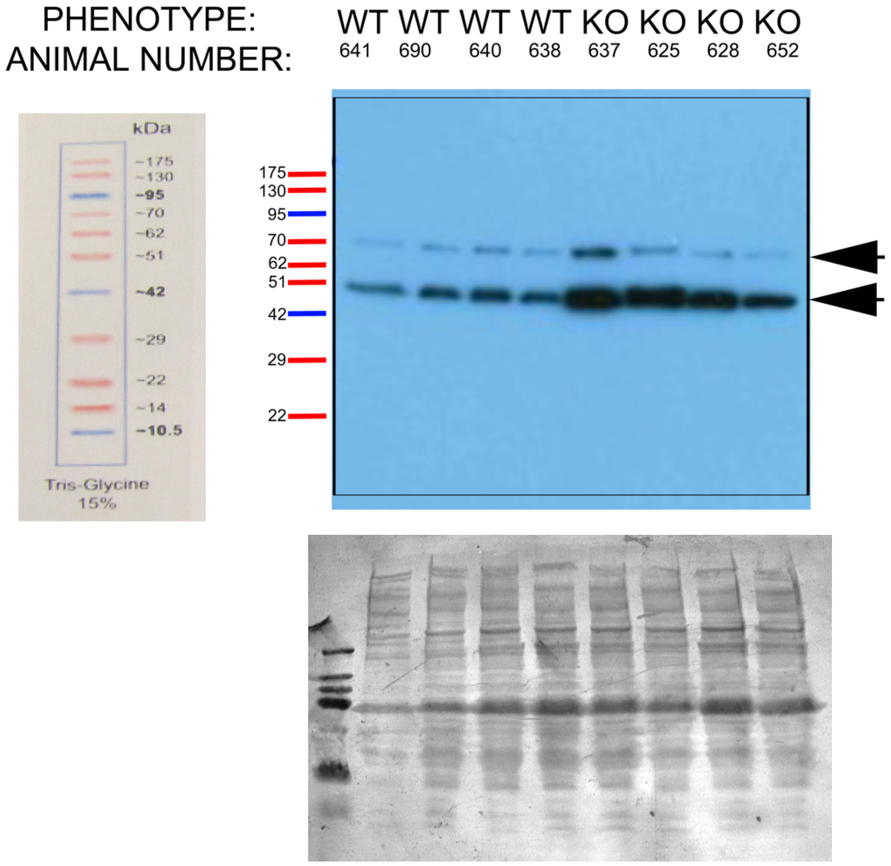

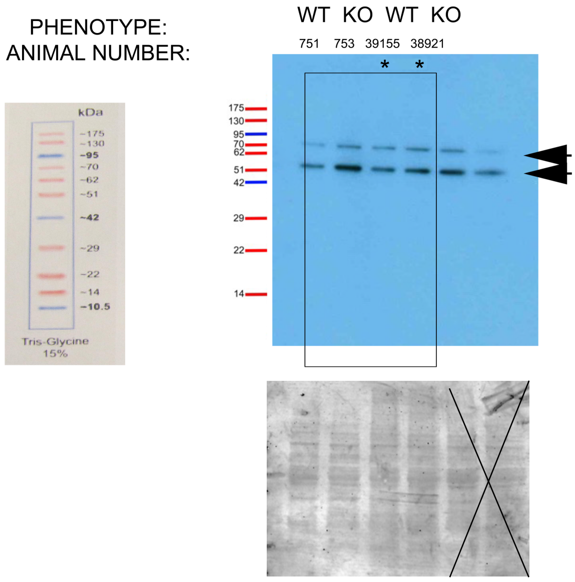

FIGURE 5

MFP1

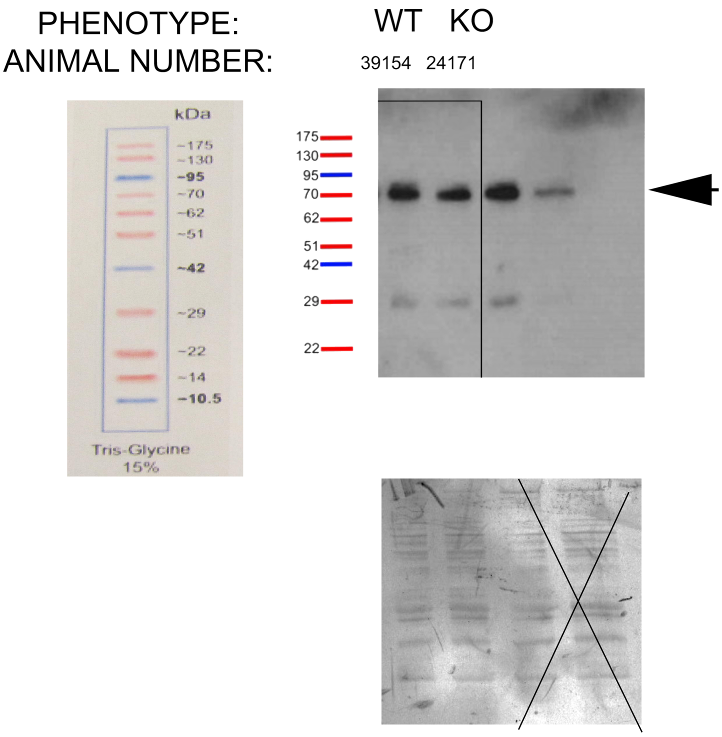

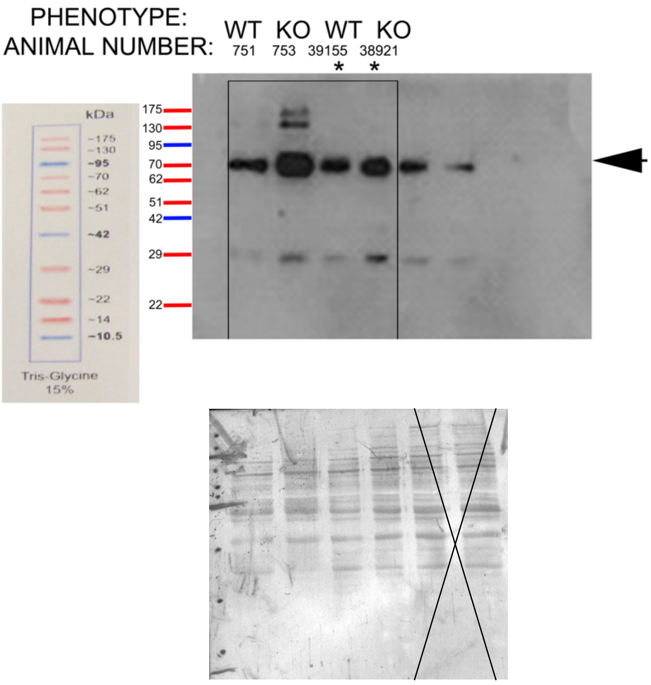

FIGURE 5

THIOLASE

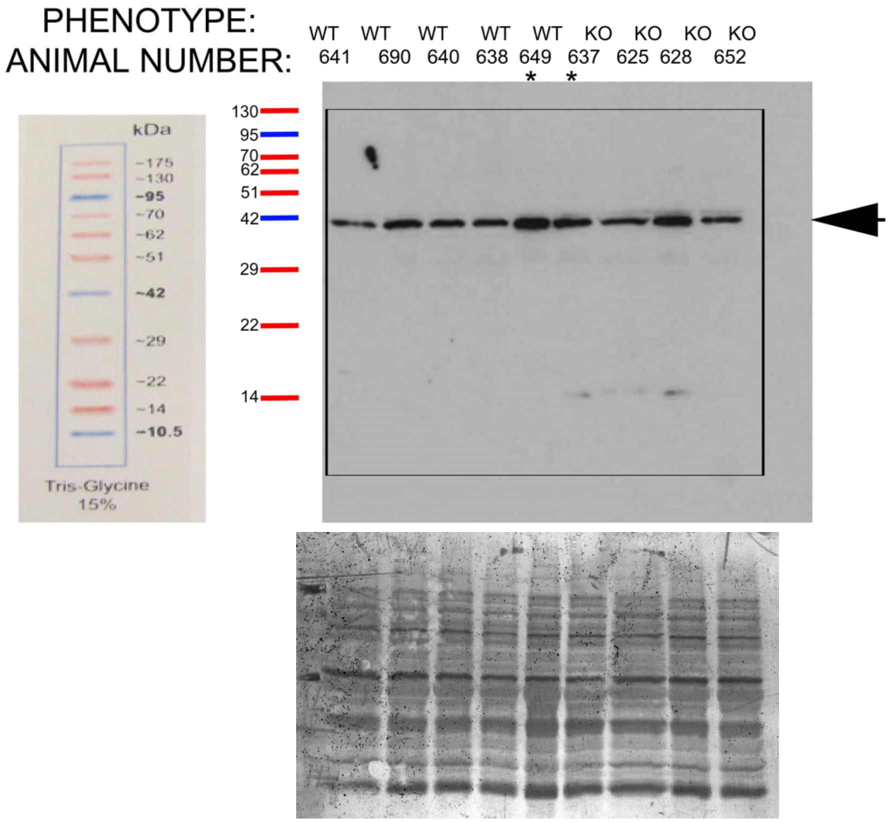

FIGURE 6

CATALASE

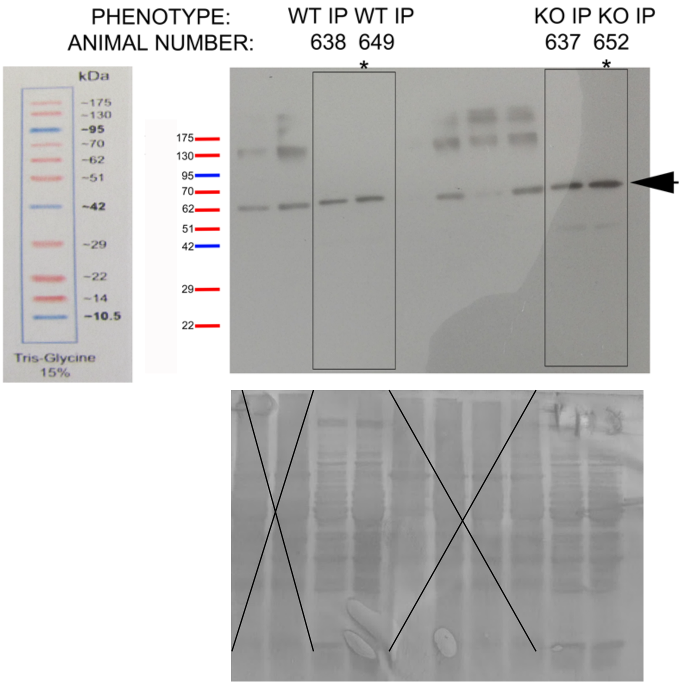

FIGURE 6

PEX14

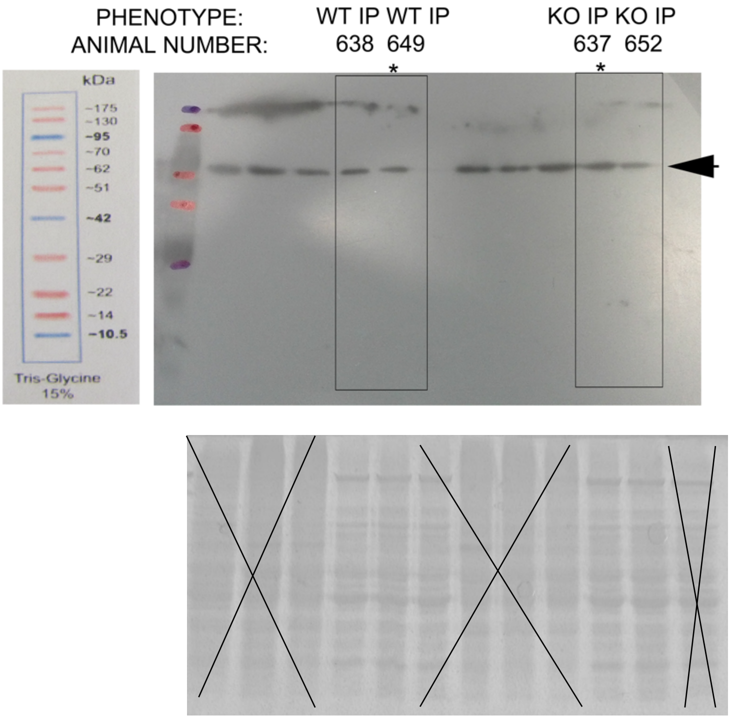

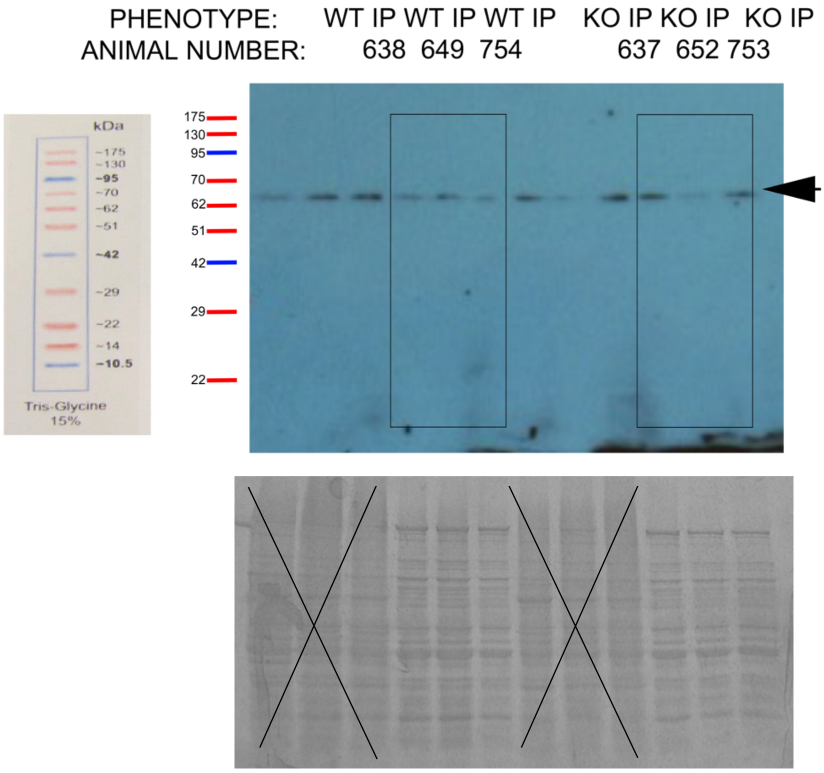

FIGURE 6

PEX19

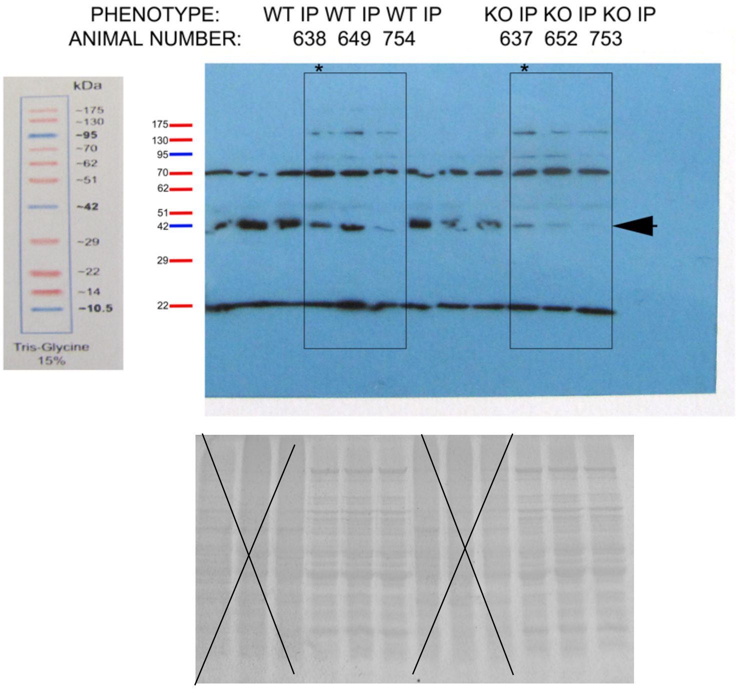

Supplement: Supplementary file 1 [file cells-15-00012-s001.zip › Original Images_/Supplemental Original Western Blots .pdf]
