# Supplementary material for: The Knockout of PEX11a Results in Mild Peroxisomal Dysfunction and Lowered Cardiac Recovery Following Langendorff-Mediated Ischemia–Reperfusion in Mice
Source: Cells. 2025 Dec 20;15(1):12. doi: 10.3390/cells15010012 (PMC12786121; doi:10.3390/cells15010012)
Supplement: Supplementary file 1 [file cells-15-00012-s001.zip › Supplemetal material/Supplemental Figures and Tables.pdf]

Supplemental figures and tables

The knockout of PEX11a results in mild peroxisomal dysfunction and impaired cardiac recovery following Langendorff-mediated ischemia-reperfusion in mice

Claudia Colasante[1*], Jiangping Chen[2], Vannuruswamy Garikapati[3], Bernhard Spengler[4], Klaus-Dieter Schlüter[5], Eveline Baumgart-Vogt[6*]

[1] Institute for Anatomy and Cell Biology, Justus Liebig University, Aulweg 123, 35392 Giessen, Germany; claudia.colasante@anatomie.med.uni-giessen.de

[2] Institute for Anatomy and Cell Biology, Justus Liebig University, Aulweg 123, 35392 Giessen, Germany; UKGM Giessen, Medical Clinic and Polyclinic II, Klinikstraße 33, 35392 Gießen, Germany; jiangping.chen@anatomie.med.uni-giessen.de

[3] Institute for Anatomy and Cell Biology, Justus Liebig University, Aulweg 123, 35392 Giessen, Germany; Max Planck Institute of Molecular Cell Biology and Genetics, Pfotenhauerstrasse 108, 01307 Dresden, Germany; garikapa@mpi-cbg.de

[4] Institute of Inorganic and Analytical Chemistry, Justus Liebig University, Heinrich-Buff-Ring 17, 35392 Giessen, Germany; bernhard.spengler@anorg.chemie.uni-giessen.de

[5] Institute for Physiology, Justus Liebig University, Aulweg 129, 35392 Giessen, Germany. klaus-dieter.schlueter@physiologie.med.uni-giessen.de

[6] Institute for Anatomy and Cell Biology, Justus Liebig University, Aulweg 123, 35392 Giessen, Germany; eveline.baumgart-vogt@anatomie.med.uni-giessen.de

* Correspondence: C.C.: claudia.colasante@anatomie.med.uni-giessen.de; E.B.V.: eveline.baumgart-vogt@anatomie.med.uni-giessen.de

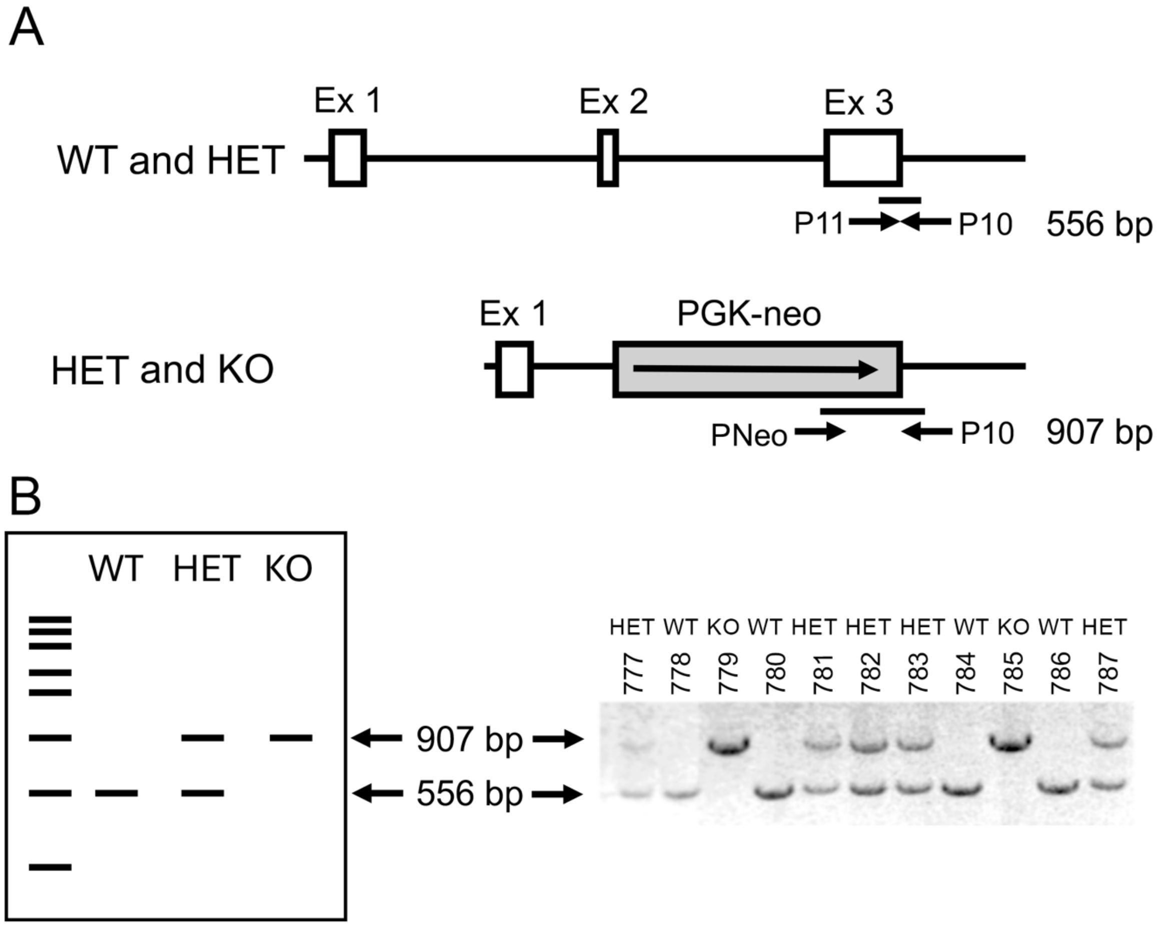

Supplemental Figure S1: **Genotyping of the PEX11a mice** A: Schematic depiction of the PEX11a gene locus showing the location of the exons 1 to 3 in the wild-type and the location of exon 1 and of the PGK-Neo cassette in the knockout mice. The hybridization site of the genotyping primers P10, P11 and PNeo is shown using arrows and the size of the expected PCR fragments is indicated in base pair (bp). B: Schematic representation of the expected multiplex PCR results and exemplary PCR results from 11 PEX11a mice (animal numbers 777 to 787). During multiplex PCR, for WT only the 556 bp band is amplified, for HET both the 556 and the 907 bp bands and for the KO only the 907 bp band. All PCRs display the band of the expected size. Results are derived from male and female mice.

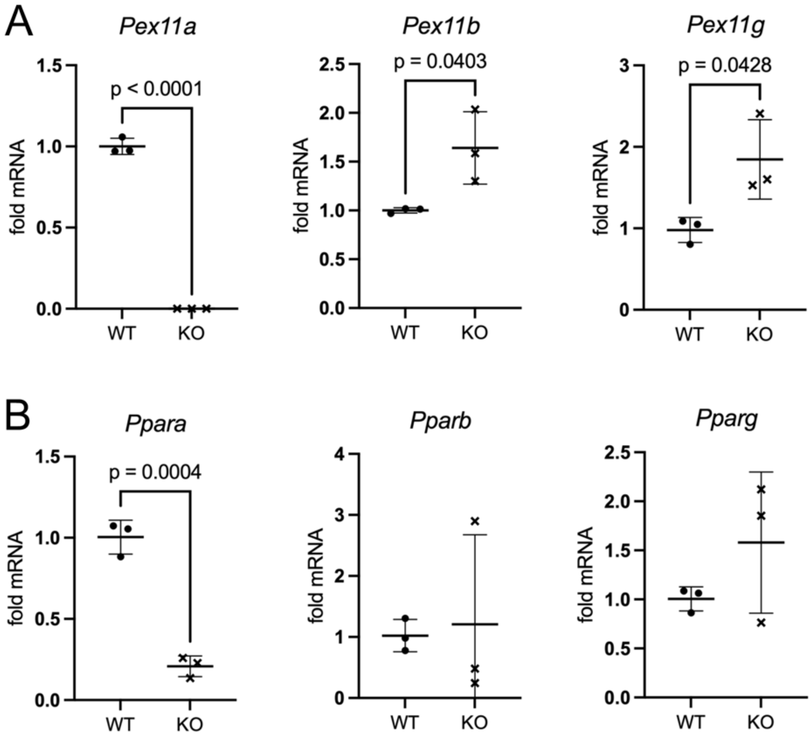

Supplemental Figure S2: **RT-qPCR analysis of the transcript abundance of the *Pex11* and *Ppar* gene families.** cDNA was synthesised from left ventricle mRNA from 3 35-week-old wild-type and knockout *Pex11a* mice and subjected to RT-qPCR. For calculations the wild-type CT value was set to one. The representative graphs are showing the results for the genes coding for A: the *Pex11* family members; B: nuclear receptors of the *Ppar* family. The symbols • for wild-type and **x** for knockout represent the calculated fold mRNA for one animal. Statistical analyses were conducted with GraphPad Prism 9 using the unpaired t-test. The graphs represent the mean and standard deviations. Significant (< 0.05) p-values are indicated above the individual comparison. Results are derived from male and female mice. Abbreviations. WT: wild-type *Pex11a* mice; KO: *Pex11a* knockout mice.

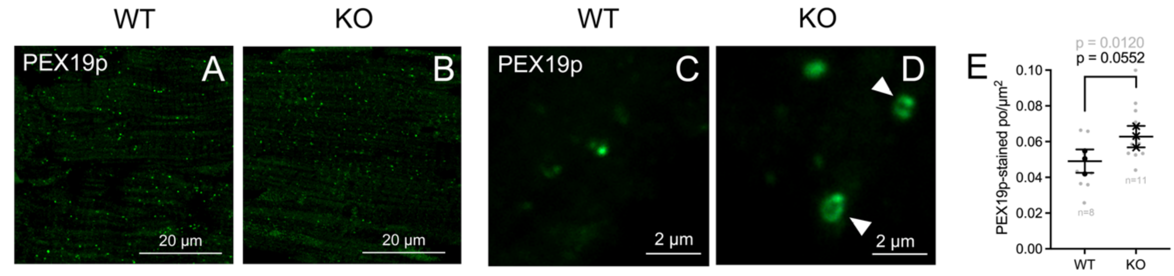

Supplemental Figure S3: Immunofluorescence analysis and morphometry of peroxisomes labelled with the peroxisomal marker PEX19p. A-D: Left ventricles were stained with an antibody against PEX19p. Images C and D show images of peroxisomes from wild-type and knockout mouse hearts at high magnification. White arrowheads indicate enlarged peroxisomes in the knockout. Peroxisome counting and tissue area definition (μm^2) shown in (E) were performed with ImageJ using a total of "n" (indicated in the graph) images from 3 mice for each phenotype. The symbols • for wild-type and **x** for knockout represent the average peroxisomes/μm^2 for 3 individual animals. The grey dots in the background represent the peroxisome number for each individual image (corresponding p-value in grey). Statistical analysis was conducted with GraphPad Prism 9 using the unpaired t-test. The graph represents the means and standard deviations. The p-value is indicated above the comparison. Scale bars are indicated in the images. Results are derived from male and female mice. Abbreviations. WT: wild-type *Pex11a* mice; KO: *Pex11a* knockout mice.

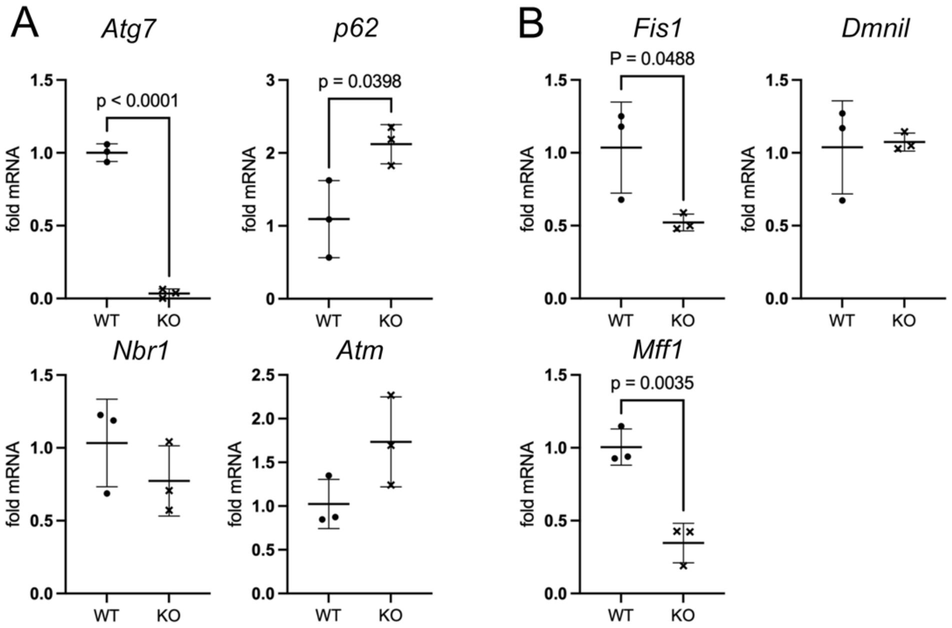

Supplemental Figure S4: Modified expression of genes involved in peroxisomal fission and pexophagy in the *Pex11a* KO heart. A and B: RT-qPCR analysis of the transcript abundance of genes coding for pexophagy (A) and peroxisomal fission (B). cDNA from left ventricle mRNA derived from 3 wild-type and knockout *Pex11a* mice aged 35 weeks was subjected to RT-qPCR using primers directed against genes coding for markers of pexophagy and fission as indicated above the graphs. The wild-type CT value was set to one. Statistical analyses of 3 independent experiments was conducted with GraphPad Prism 9 using the unpaired t-test. The graphs represent the mean and standard deviations. Significant (< 0.05) p-values are indicated above the individual comparison. Results are derived from male and female mice. Abbreviations.: WT: wild-type *Pex11a* mice; KO: *Pex11a* knockout mice.

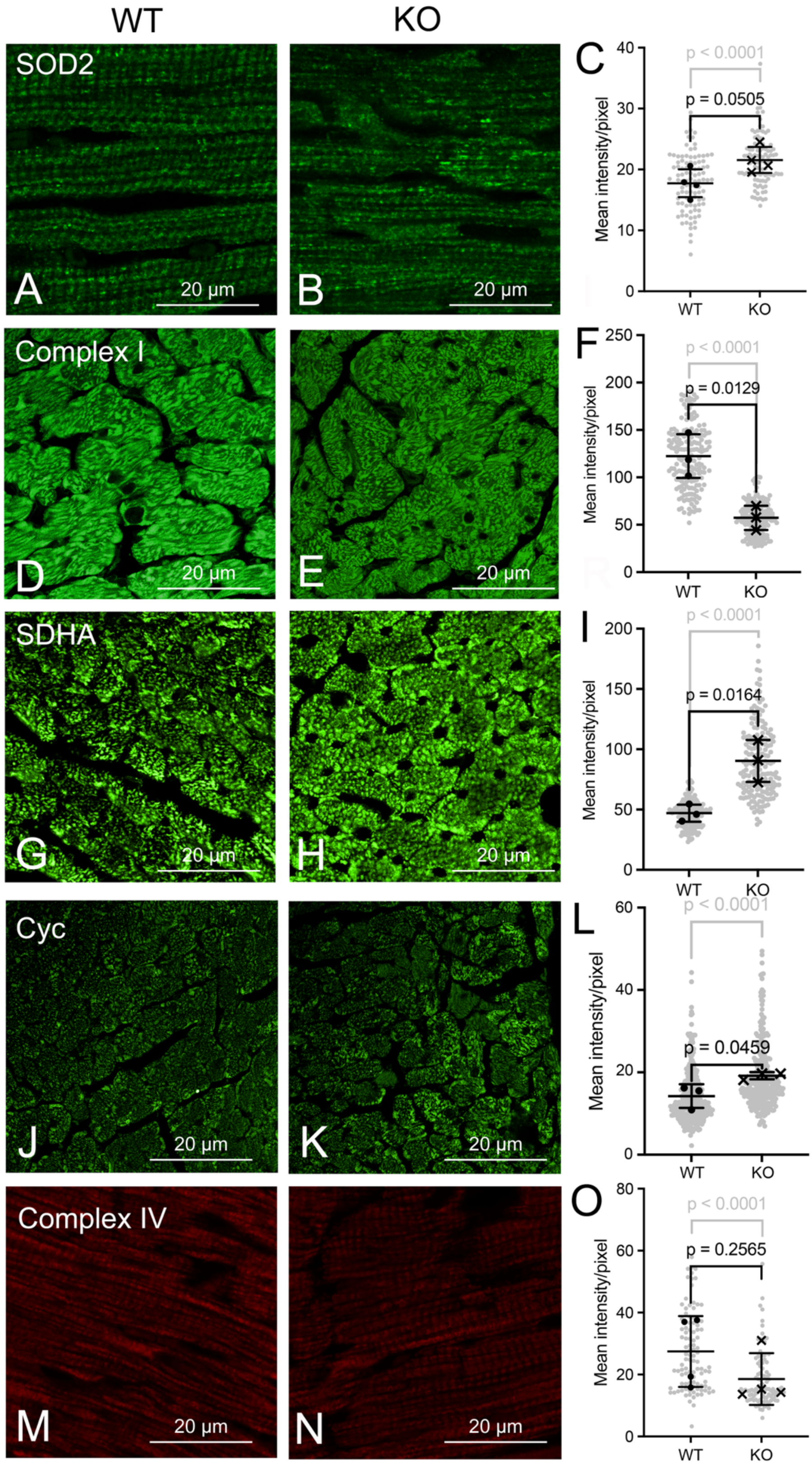

Supplemental Figure S5: Immunofluorescence analysis of the LVs from wild-type and knockout cardiomyocyte mitochondria. Immunofluorescence analysis of the LVs from wild-type and knockout hearts using SOD2 (A-B), Complex I (D-E), SDHA (G-H), Cyc (J-K) and

Complex IV (M-N) antibodies and corresponding mean intensity/pixel (C, F, I, L and O) calculated from individual cardiomyocytes using the image acquisition program Zen_2.3_sp1 and Laser scanning microscope lsm_710 (Zeiss). For SOD2 100 cardiomycytes were analysed (from 3 mice), for Complex I 180 cardiomyocytes (from 3 mice), for SDHA 160 cardiomycytes (from 3 mice), for Cyc 300 cardiomyocytes (from 3 mice) and for Complex IV 100 cardiomyocytes (from 4 mice). In C, F, I, L and O symbols "•" (wild-type) and "**x**" (knockout) represent average values for the individual animals. The background grey clouds represent intensity/pixel values per cardiomyocyte (corresponding p-value in grey). Statistical analyses were conducted with GraphPad Prism 9 using the unpaired t-test. The graphs represent the mean and standard deviations. Significant (< 0.05) p-values are indicated above the individual comparison. Scale bars are indicated in the images. Results are derived from male and female mice. Abbreviations.: WT: wild-type *Pex11a* mice; KO: *Pex11a* knockout mice.

Supplemental Figure S6: High-resolution AP-S MALDI MS imaging of wild-type and *Pex11a* knockout mouse heart. Comparative analysis of endogenous metabolites (tentative assignments based on accurate mass) between wild-type and *Pex11a* knockout mouse heart sections using MALDI MS imaging. Abbreviations.: WT: wild-type *Pex11a* mice; KO: *Pex11a* knockout mice.

CAR(8:0;O) **WT** **KO**

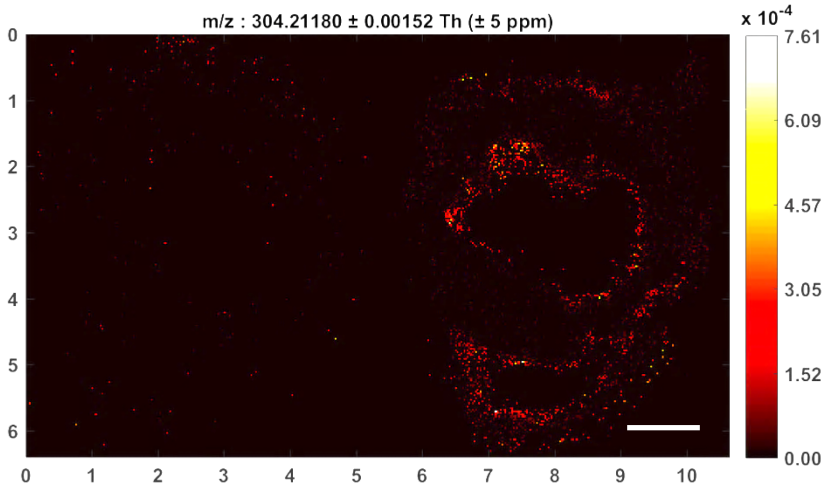

CAR(10:0;O) **WT** **KO**

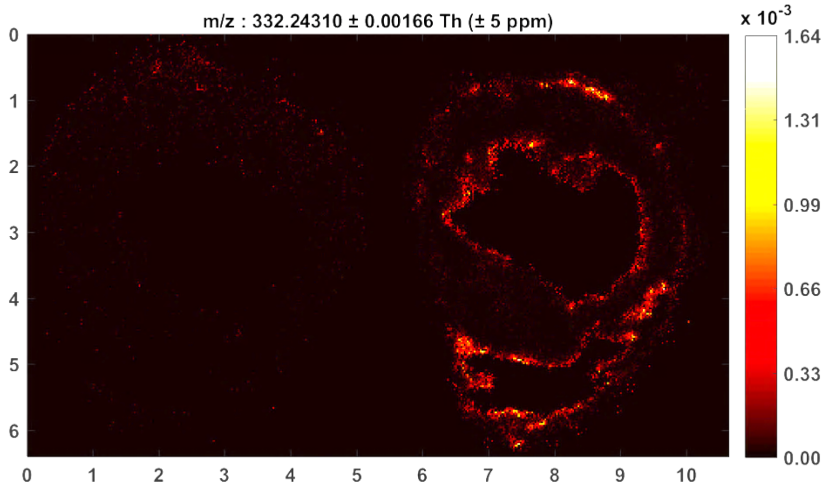

CAR(12:1;O) **WT** **KO**

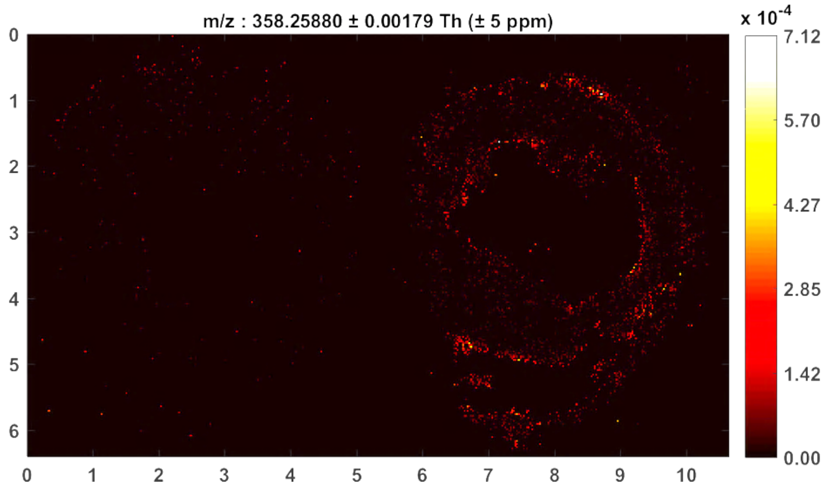

CAR(12:0;O) **WT** **KO**

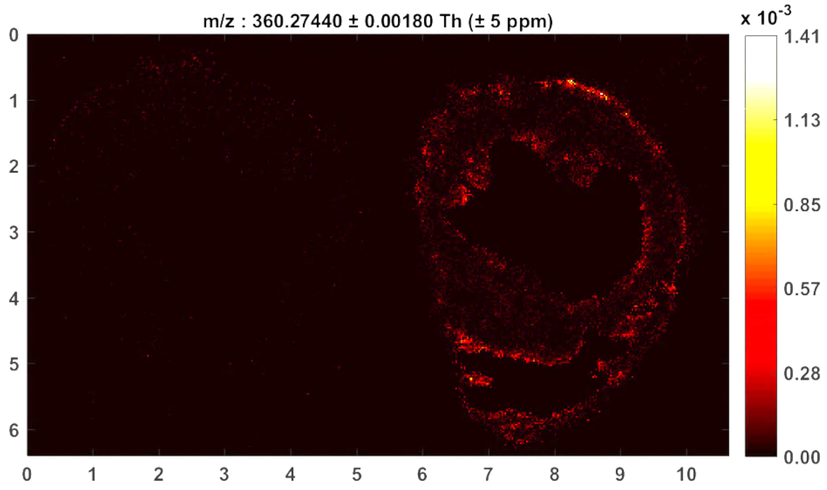

CAR(14:0) **WT** **KO**

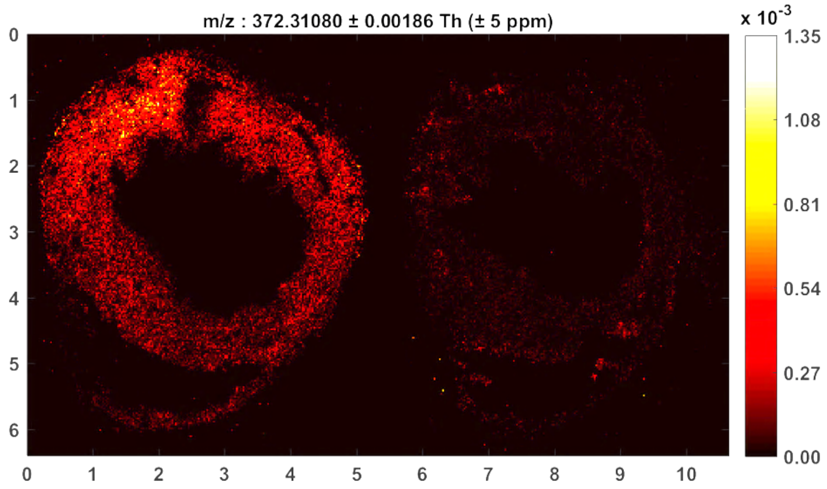

CAR(14:0;O) **WT** **KO**

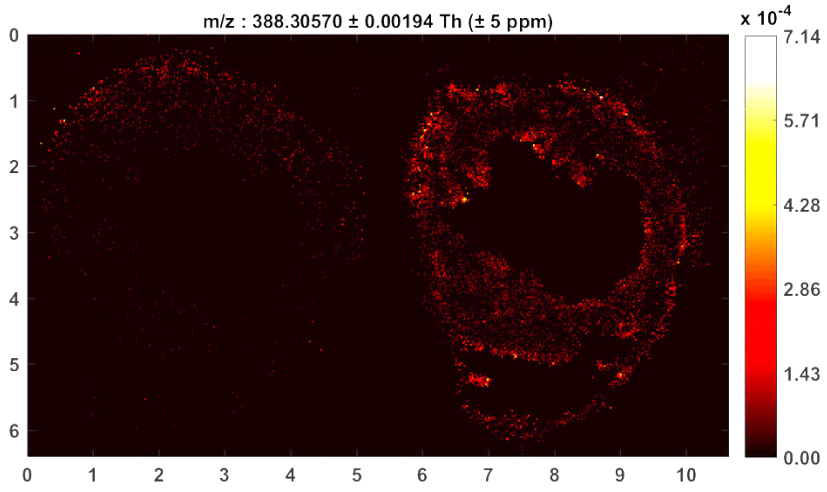

CAR(16:2) **WT** **KO**

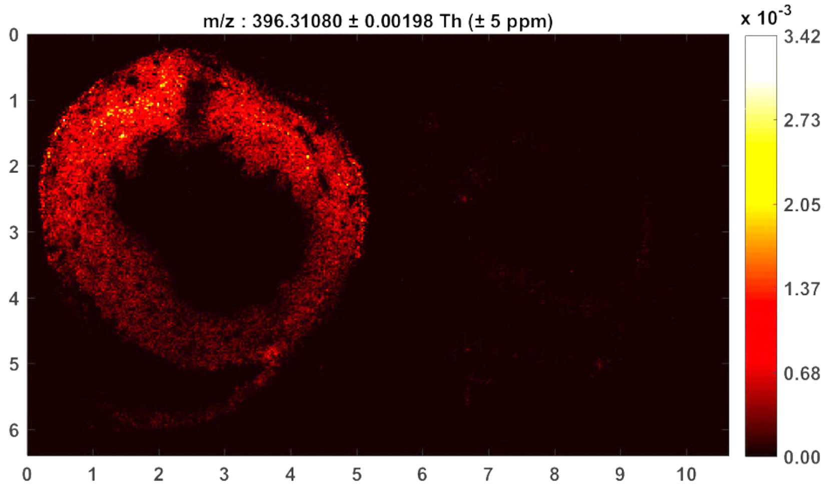

CAR(16:1) **WT** **KO**

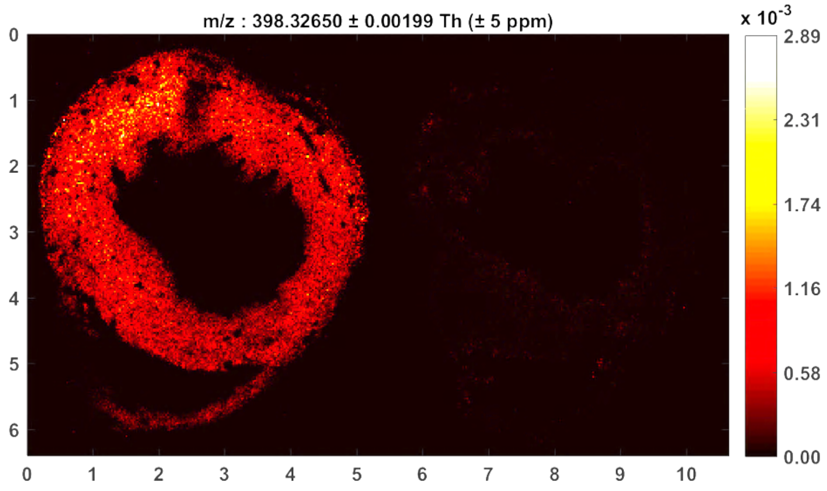

CAR(16:0) **WT** **KO**

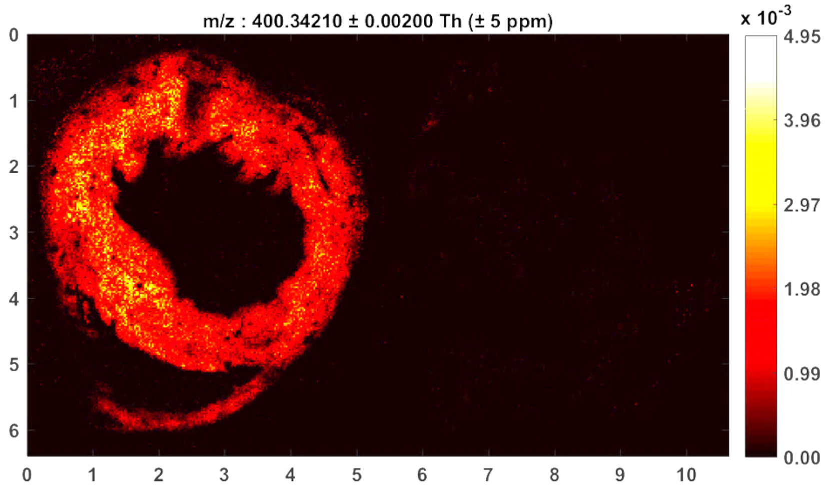

CAR(18:3) **WT** **KO**

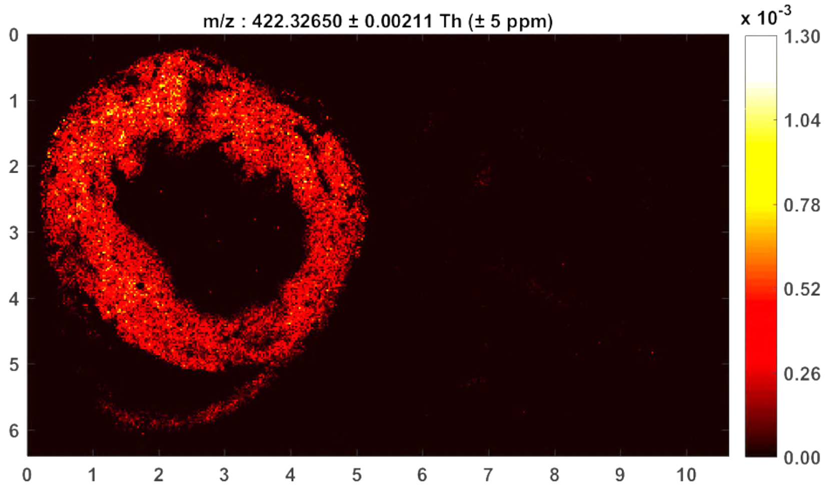

CAR(18:2) **WT** **KO**

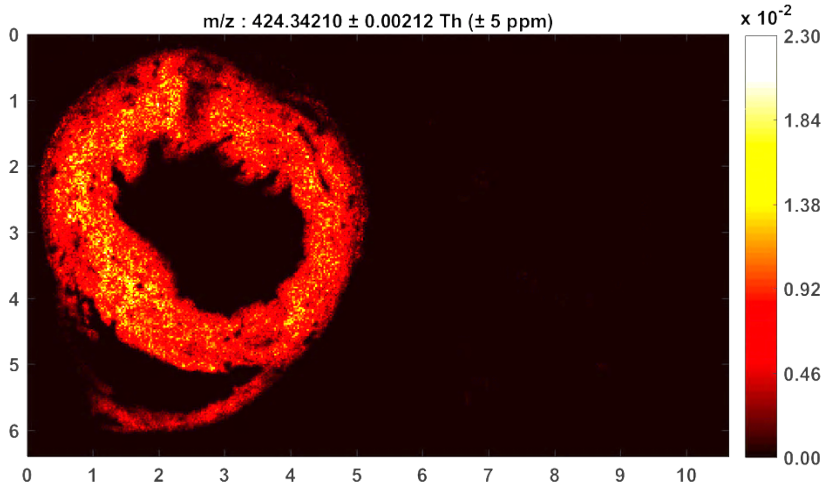

CAR(18:1) **WT** **KO**

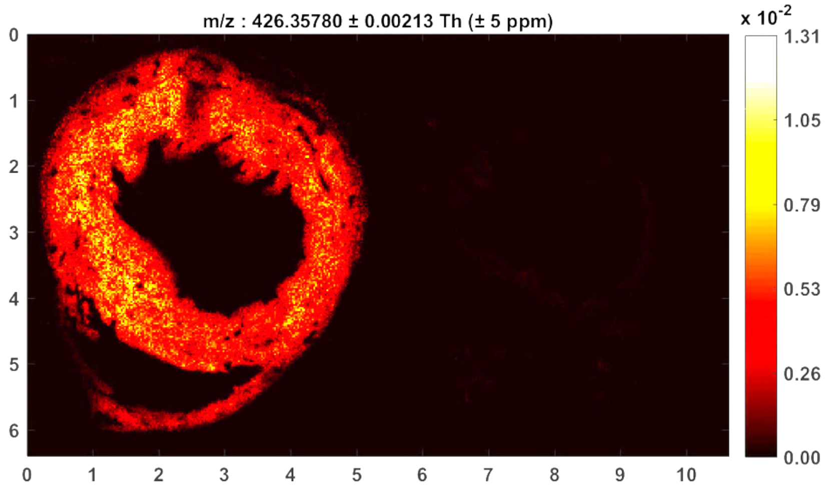

CAR(18:0) **WT** **KO**

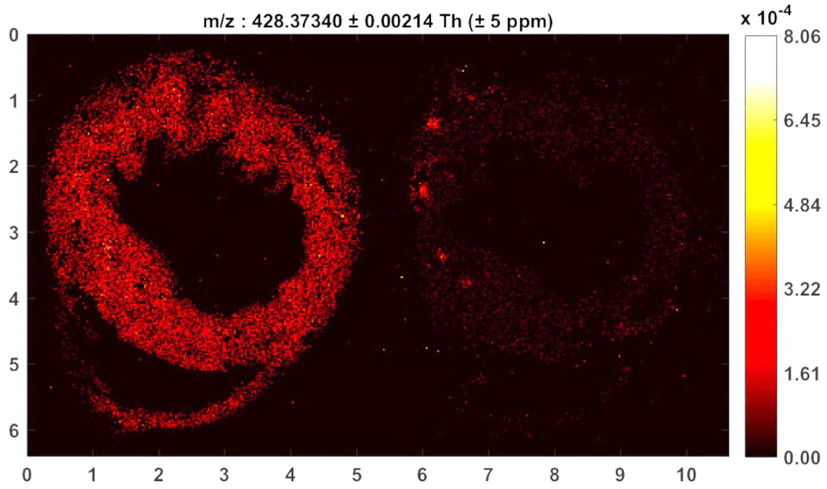

CAR(16:0) / LPE(P-16:0) **WT** **KO**

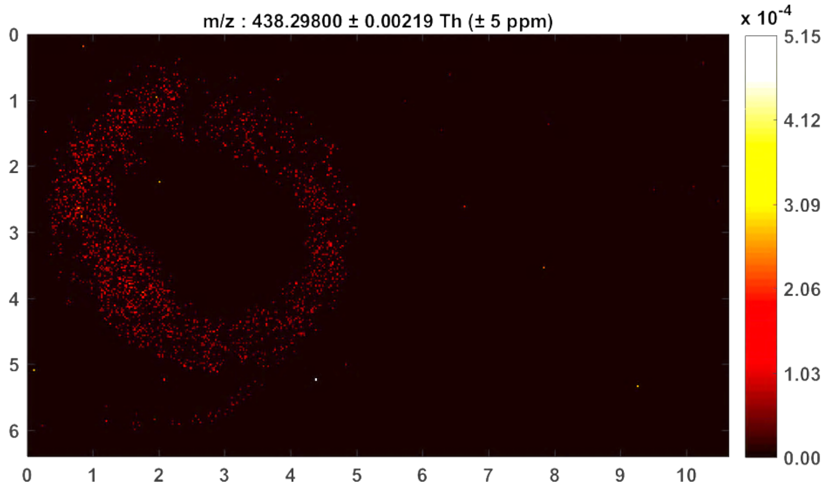

CAR(18:2) WT KO

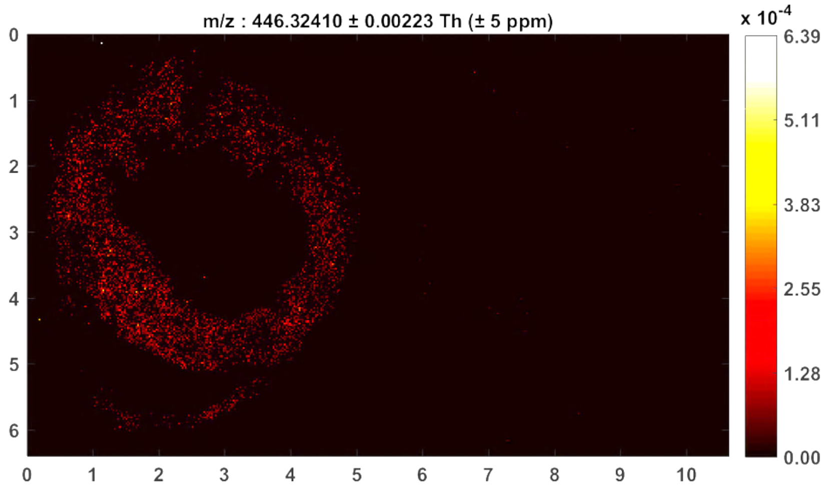

CAR(20:4) WT KO

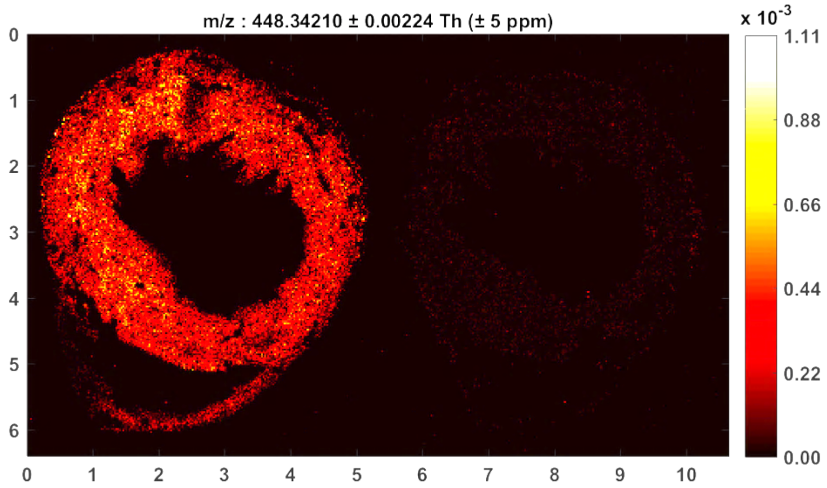

CAR(20:1) WT KO

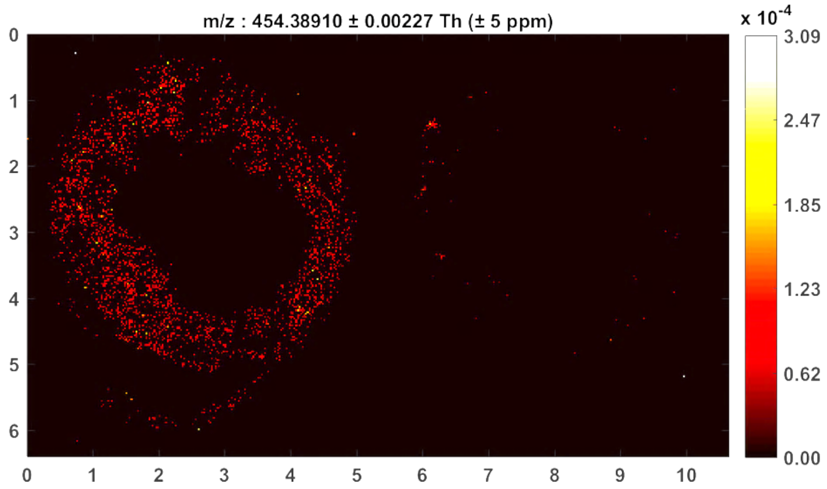

CAR(18:2) **WT** **KO**

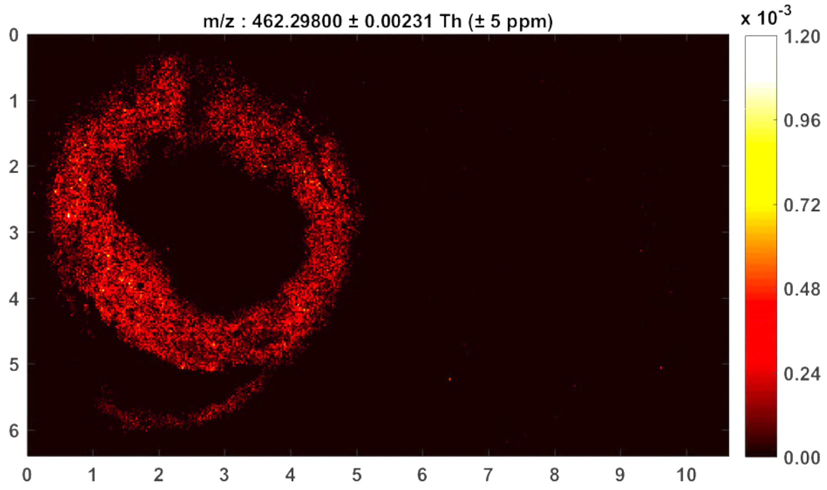

CAR(18:1) / LPE (P-18:1) **WT** **KO**

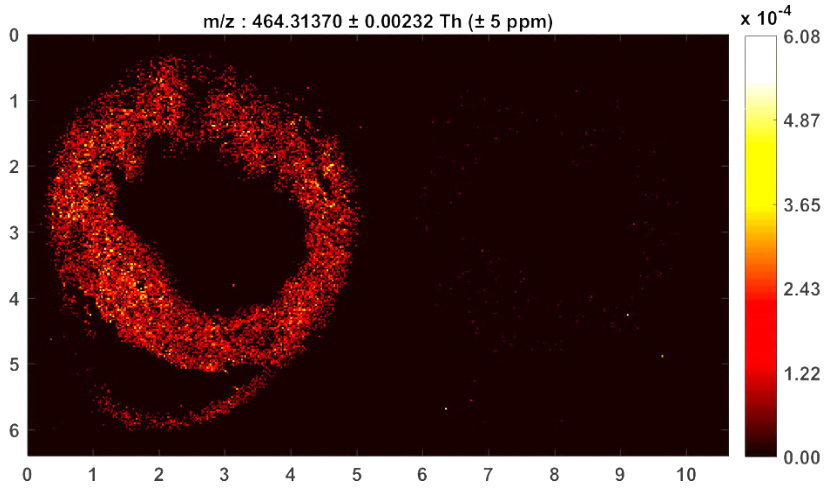

LPC(16:1) **WT** **KO**

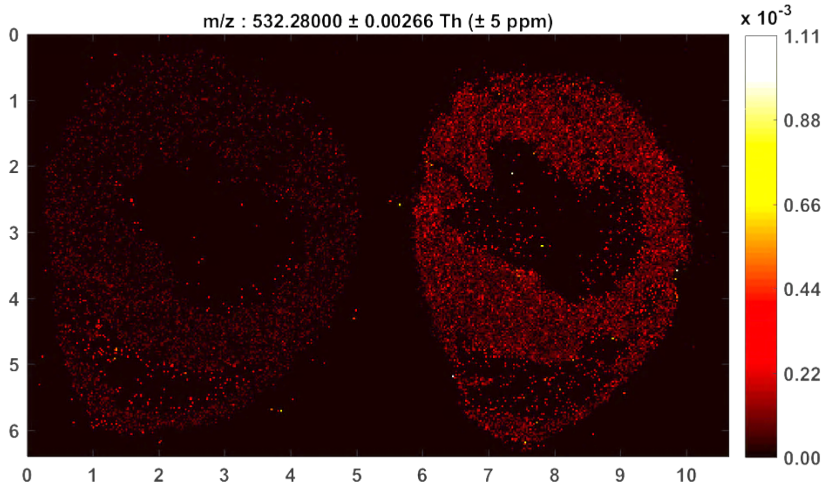

LPE(24:6) WT KO

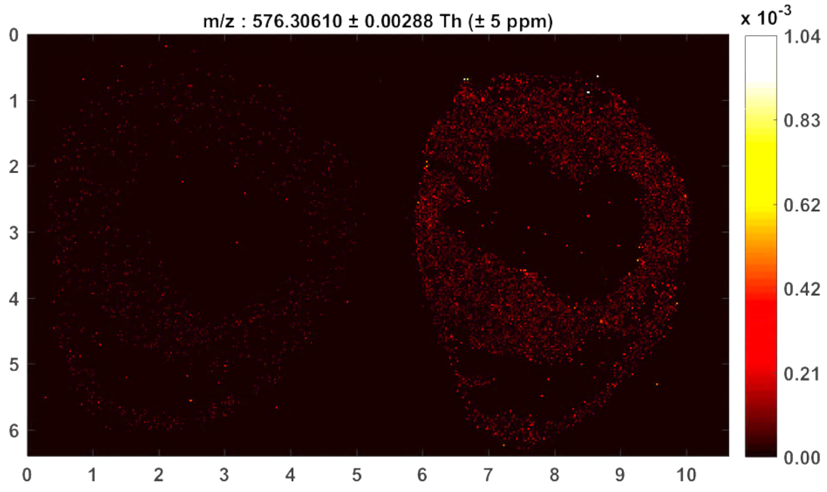

DG(36:4) WT KO

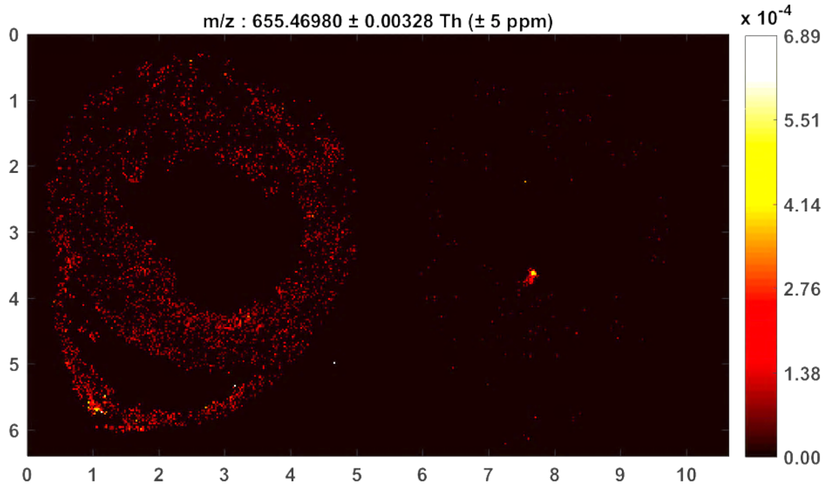

LPC(26:0) WT KO

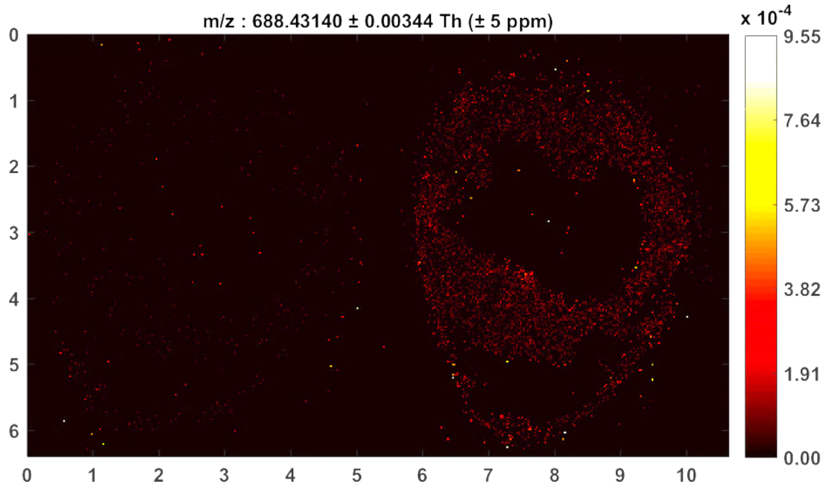

PS(28:0) **WT** **KO**

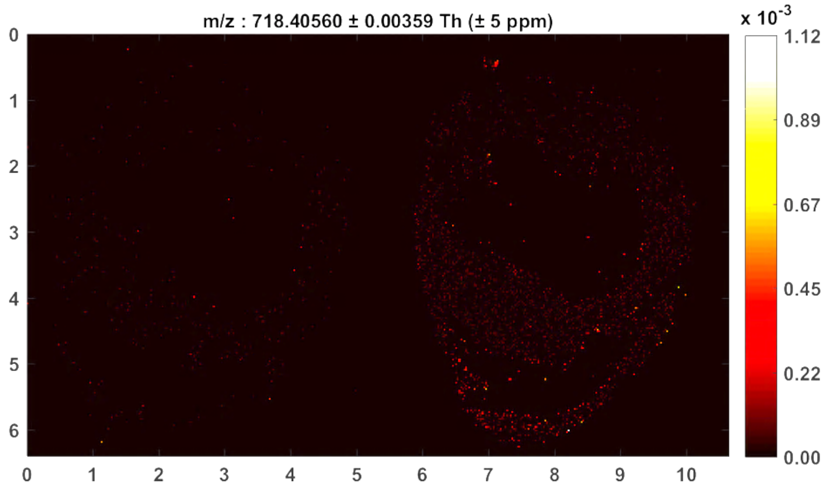

PC(P-34:1) / PC(O-34:2) **WT** **KO**

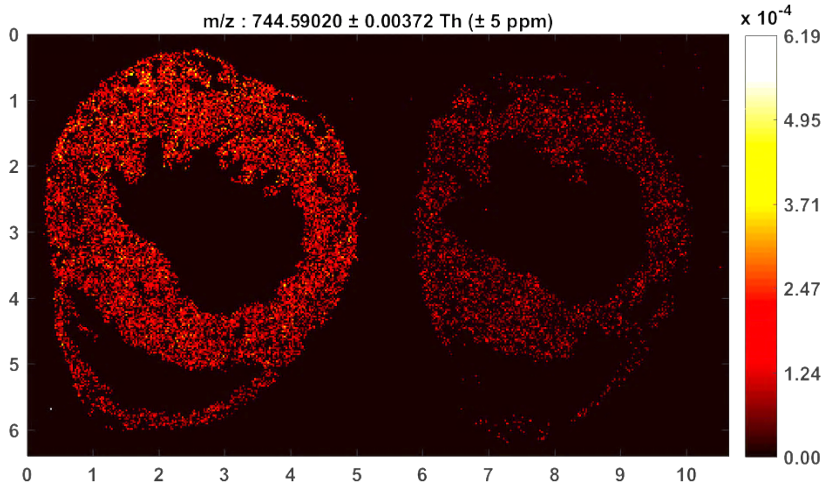

PC(P-34:0) / PC(O-34:1) **WT** **KO**

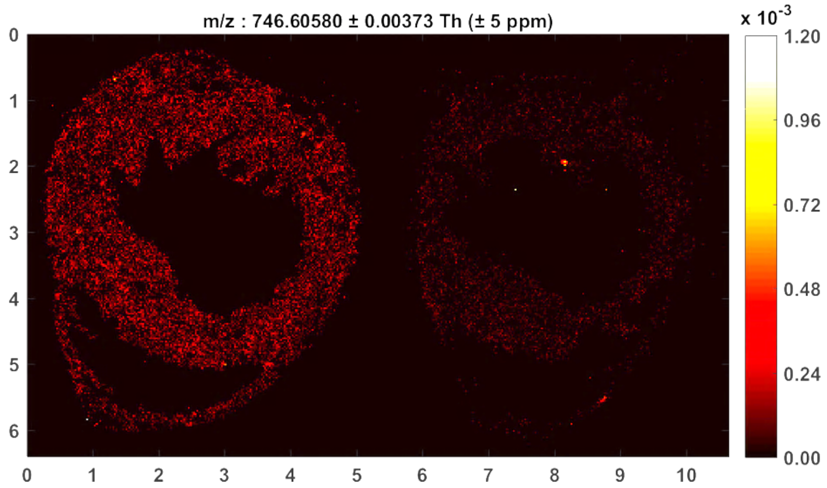

PE(34:2) **WT** **KO**

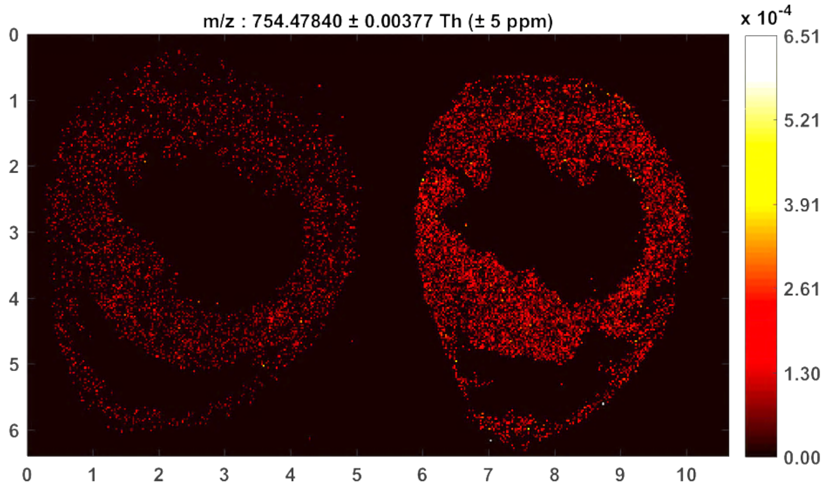

PC(32:2) **WT** **KO**

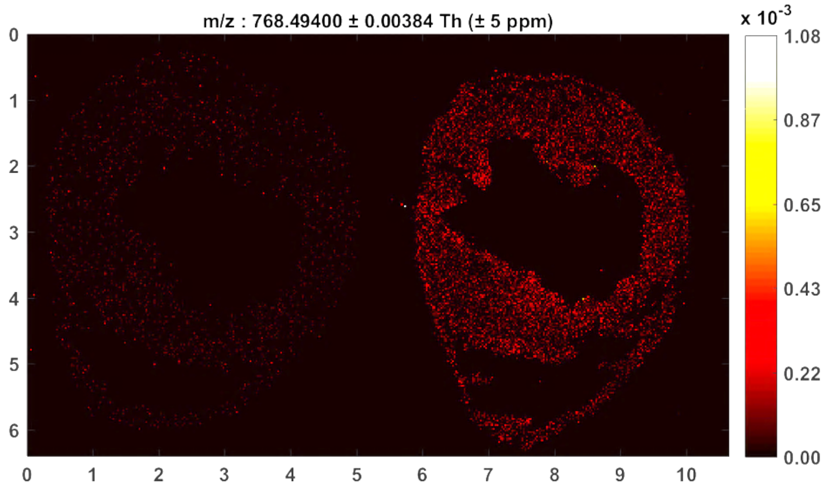

PE (36:3) **WT** **KO**

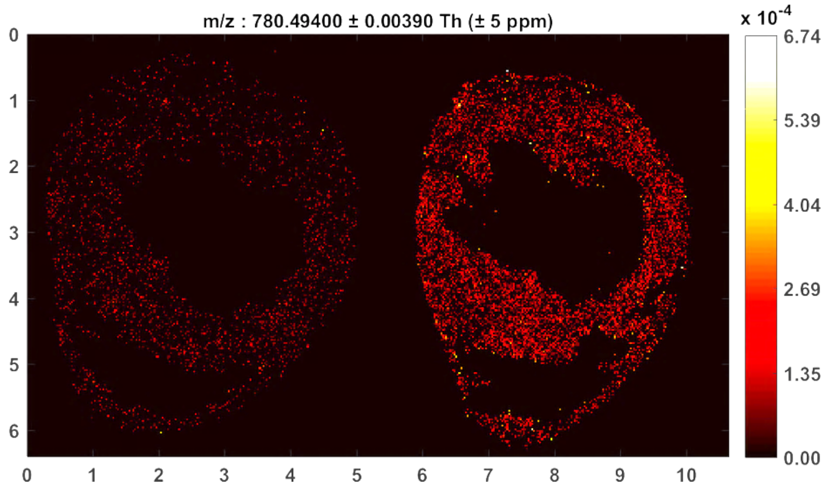

PE(42:11) **WT** **KO**

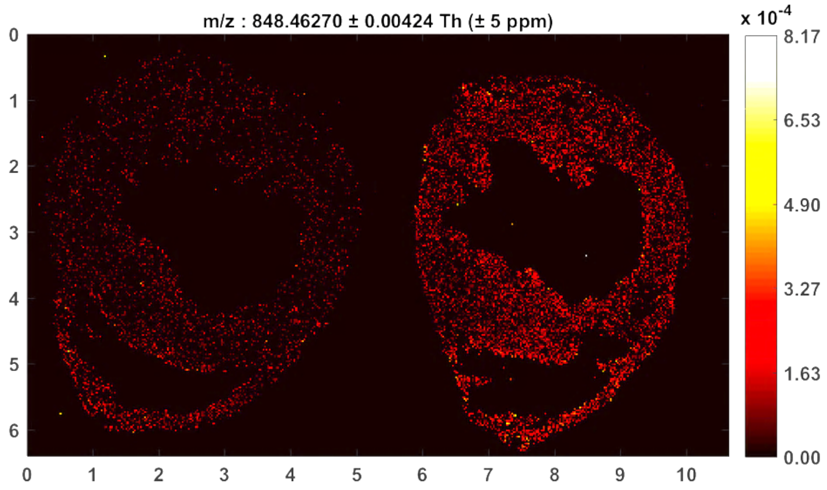

PGP(34:2) **WT** **KO**

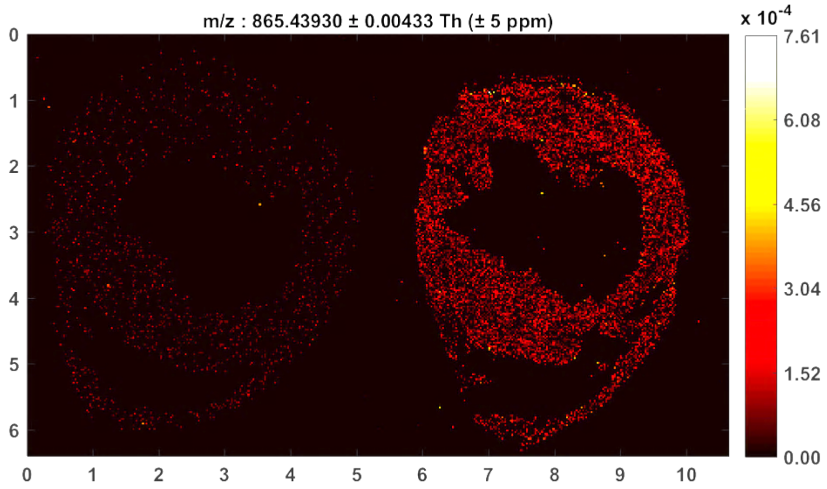

PC(42:10) **WT** **KO**

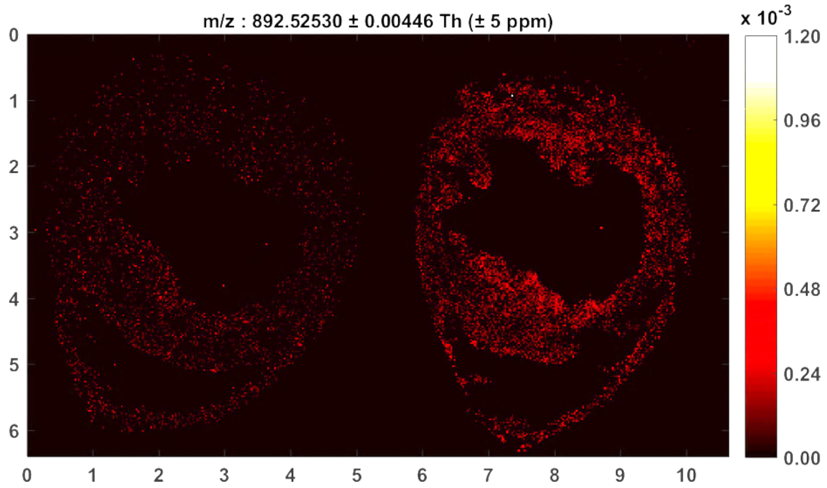

PS(42:7) **WT** **KO**

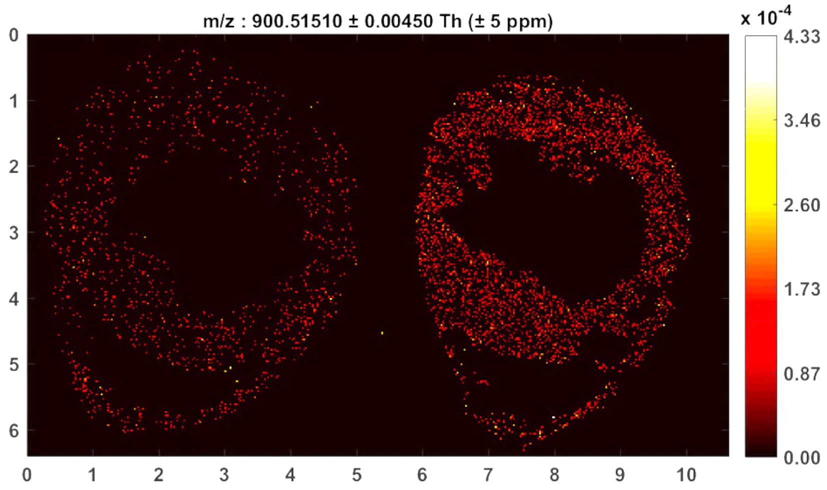

TG(54:5) **WT** **KO**

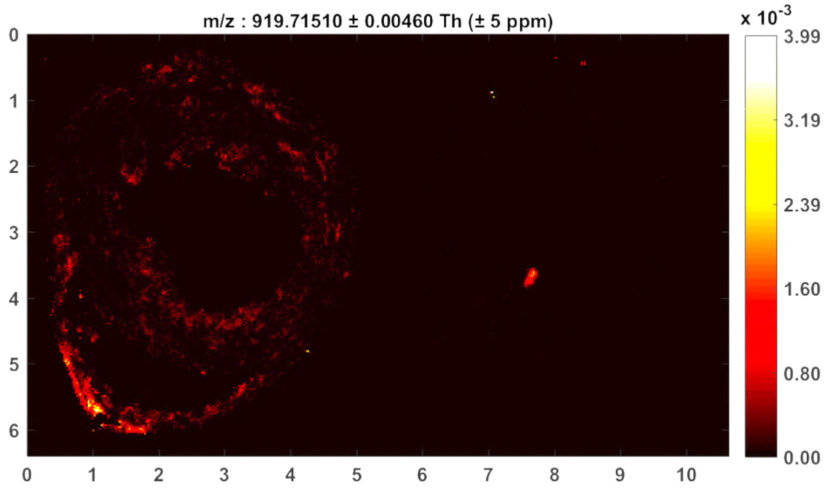

TG(56:7) **WT** **KO**

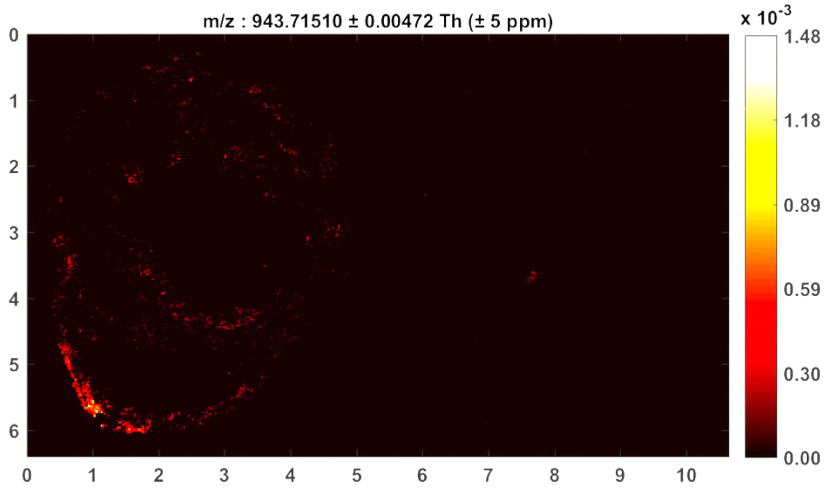

TG(56:5) **WT** **KO**

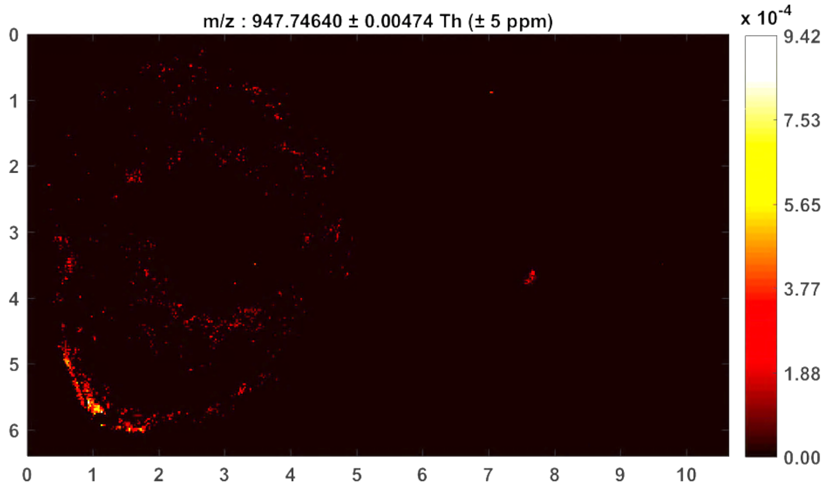
m/z : 947.74640 ± 0.00474 Th (± 5 ppm)
x 10^-4
9.42
7.53
5.65
3.77
1.88
0.00
0
1
2
3
4
5
6
0 1 2 3 4 5 6 7 8 9 10

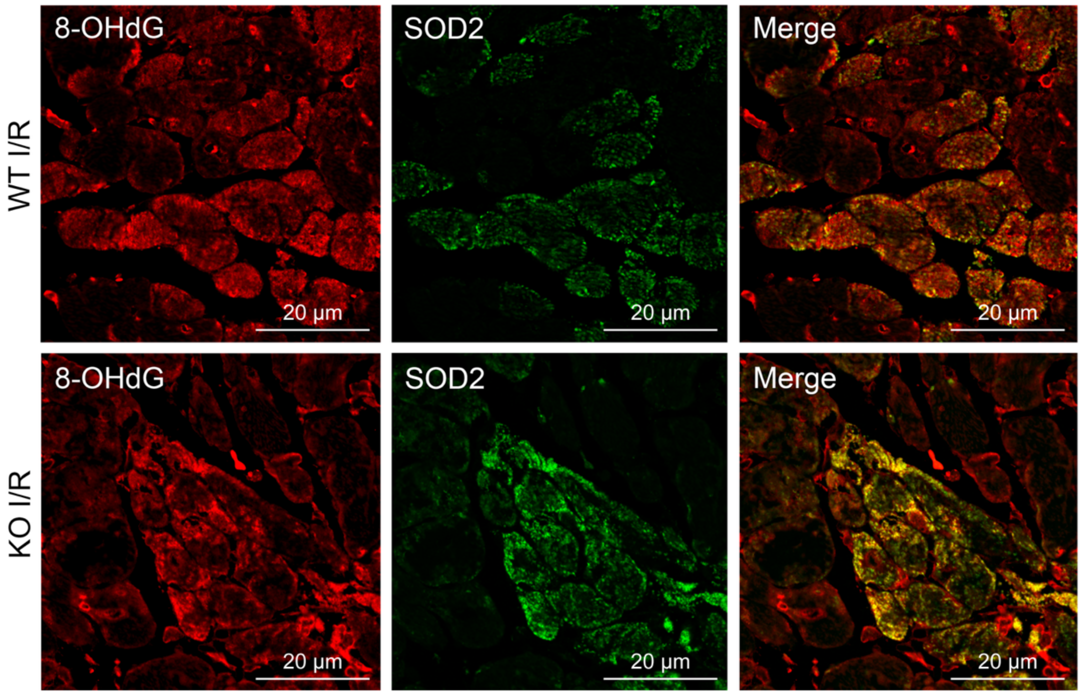

Supplemental Figure S7: Colocalization of 8OHdG with the mitochondrial marker SOD2. Immunofluorescence analysis of the left ventricles from I/R-injured wild-type (WT) and *Pex11a* knockout (KO) mice hearts using antibodies directed against the oxidative marker 8OHdG and SOD2 to show their colocalization in mitochondria. Scale bars are indicated in the images. Results are derived from male and female mice.

SUPPLEMENTAL TABLES

Primer name	Sequence
P10	5’-AATCAGGGACCTGTGCAACCTG-3
P11	5’-AGTACAGCGTGGCTAATGAAGAGAC-3’
PNeo	5’-ATATTGCTGAAGAGCTTGGCGGC-3’

Supplemental Table S1 List of primers for genotyping

Gene	Forward primer sequence	Reverse primer sequence
Pex11a	ACTGGCCGTAAATGGTTCAGA	CGGTTGAGGTTGGCTAATGTC
Pex11d	GCCCAGTATGCCTGTTCCC	CTCCAGTTGTCGAATCTGTTTCT
Pex11g	GACTCTGCTTGGTGGTGGA	GCTCCGCAGCTTCTGTCTAA
PPARa	AGACCCTCGGGGAACTTAGA	CAGAGCGCTAAGCTGTGATG
PPARb	GCAGCCTCAACATGGAATGTC	GAGCTTCATGCGGATTGTCC
PPARg	TTTTCAAGGGTGCCAGTTTC	CATGGACACCATACTTGAGCA
Arginase 1	CAGAAGAATGGAAGAGTCAG	CAGATATGCAGGGAGTCACC
Nppa	GAACCTGCTAGACCACCT	CCTAGTCCACTCTGGGCT
Nppb	AAGCTGCTGGAGCTGATAAGA	GTTACAGCCCAAACGACTGAC
Mhc6	CTGCTGGAGAGGTTATTCCTCG	GGAAGAGTGAGCGGCGCATCAAGG
Mhc7	TGCAAAGGCTCCAGGTCTGAGGGC	GCCAACACCAACCTGTCCAAGTTC
b-Actin	CTAGGCACCAGGGTGTGATG	GGGGTACTTCAGGGTCAGGA
Atg7	GGTTGCAGGCAAATCAGGC	AGGCGGTACTCGTTCAACTT
P62	TCTACAGAGGCTGATCCCCG	CTTGGCCACAGCACTATCAC
Fis	AGGCTCTAAAGTATGTGCGAGG	GGCCTTATCAATCAGGCGTTC
Dnmil	TTTCAGAGCTGGAACCCTGC	GAGATGGATTGGCTCAGGGC
Nbr	GGGATTTAAAGCACCTCCTGA	AATTCCCTCTGTGGGGACCT
Atm	ATATGCCAGTCTTTTCAGGGTG	GCGCTCTCTGTCTGTGACTG
Mff	TCAAAGCGAAGAGAGCCGAG	CCACGGGCAGAGGAAAGATT

Supplemental Table S2 List of primer pairs for qPCR analysis

Antigen	Method	Species	Dilution	Source and order number
8ohdg	IFA	goat	1:200	Abcam, ab93295
ABCD3	WB	rabbit	1:500	Invitrogen, PA1-650
Acox1	WB/IFA	rabbit	1:1000/1:500	Abcam, ab184032
Catalase	WB/IFA/EM	rabbit	1:4000/1:2000/1:1000	Grant *et al* [27] (D Crane)
Complex IV sub I	IFA	mouse	1:500	Invitrogen, 459600
Connexin 43	IFA	rabbit	1:400	Sigma, C6219
CYC1	IFA	rabbit	1:200	Proteintech 10242-1-AP
MFP1	WB	mouse	1:500	Abnova, H00001962-B01
NDUFS1	IFA	rabbit	1:100	Cell signaling 70264
PEX14p	WB/IFA	rabbit	1:1000/1:10,000	Grant *et al* [27] (D Crane)
PEX19p	WB/IFA	rabbit	1:5000/1:10,000	Colasante *et al* [23]
PEX3p	IFA	rat	1:300	Colasante *et al* [23]
PLIN2	IFA	rabbit	1:500	Proteintech, 15294-1AP
SDHA	IFA	rabbit	1:400	Proteintech, 14865-1-AP
SOD2	IFA	rabbit	1:500	Abcam, ab13533
Thiolase	WB	mouse	1:500	Santa cruz, 514051

Supplemental Table S3: Primary antibodies

Species	Dilution	Source and order number
donkey-anti-mouse	1:6000	Jackson, 715-005-150
donkey-anti-rabbit	1:10,000	Jackson, 711-005-152

Supplemental Table S4: Secondary antibodies for western blotting

Species	Fluorochrome	Dilution	Source
donkey-anti-rabbit	Alexafluor 488	1:1000	Molecular Probes, A21206
goat-anti-rat	Cy3	1:600	Jackson/Dianova, 712-165-153
donkey-anti-goat	Alexafluor 594	1:300	Molecular Probes, A11058
donkey-anti-mouse	Alexafluor 555	1:400	Molecular Probes, A31570

Supplemental Table S5: Secondary antibodies for immunofluorescence analysis

S.No	*m/z*	Metabolites (tentative assignments)	Molecular formula	Ion adduct	RA	RMSE (WT)	RMSE (KO)
1	304.2118	CAR(8:0;O)	$C_{15}H_{29}NO_5$	M+H	HA	1.36	0.88
2	332.2431	CAR(10:0;O)	$C_{17}H_{33}NO_5$	M+H	HA	0.88	0.74
3	358.2588	CAR(12:1;O)	$C_{19}H_{35}NO_5$	M+H	HA	1.25	0.94
4	360.2744	CAR(12:0;O)	$C_{19}H_{37}NO_5$	M+H	HA	1.03	0.74
5	372.3108	CAR(14:0)	$C_{21}H_{41}NO_4$	M+H	LA	0.59	0.78
6	388.3057	CAR(14:0;O)	$C_{21}H_{41}NO_5$	M+H	HA	0.96	0.79
7	396.3108	CAR(16:2)	$C_{23}H_{41}NO_4$	M+H	LA	0.46	0.82
8	398.3265	CAR(16:1)	$C_{23}H_{43}NO_4$	M+H	LA	0.45	0.80
9	400.3421	CAR(16:0)	$C_{23}H_{45}NO_4$	M+H	LA	0.34	0.81
10	422.3265	CAR(18:3)	$C_{25}H_{43}NO_4$	M+H	LA	0.68	1.28
11	424.3421	CAR(18:2)	$C_{25}H_{45}NO_4$	M+H	LA	0.32	0.69
12	426.3578	CAR(18:1)	$C_{25}H_{47}NO_4$	M+H	LA	0.51	0.76
13	428.3734	CAR(18:0)	$C_{25}H_{49}NO_4$	M+H	LA	0.75	0.85
14	438.2980	CAR(16:0) / LPE(P-16:0)	$C_{23}H_{45}NO_4$ / $C_{21}H_{44}NO_6P$	M+K / M+H	LA	0.86	2.11
15	446.3241	CAR(18:2)	$C_{25}H_{45}NO_4$	M+Na	LA	1.19	3.77
16	448.3421	CAR(20:4)	$C_{27}H_{45}NO_4$	M+H	LA	0.80	0.92
17	454.3891	CAR(20:1)	$C_{27}H_{51}NO_4$	M+H	LA	0.90	1.32
18	462.2980	CAR(18:2)	$C_{25}H_{45}NO_4$	M+K	LA	0.63	1.51
19	464.3137	CAR(18:1) / LPE (P-18:1)	$C_{25}H_{47}NO_4$ / $C_{23}H_{46}NO_6P$	M+K / M+H	LA	0.67	1.29
20	532.2800	LPC(16:1)	$C_{24}H_{48}NO_7P$	M+K	HA	0.93	0.81

21	576.3061	LPE(24:6)	$C_{29}H_{48}NO_7P$	M+Na	HA	1.18	1.03
22	655.4698	DG(36:4)	$C_{39}H_{68}O_5$	M+K	LA	1.01	0.61
23	688.4314	LPC(26:0)	$C_{34}H_{68}NO_8P$	M+K	HA	1.32	1.44
24	718.4056	PS(28:0)	$C_{34}H_{66}NO_{10}P$	M+K	HA	1.59	1.33
25	744.5902	PC(P-34:1) / PC(O-34:2)	$C_{42}H_{82}NO_7P$	M+H	LA	0.86	0.92
26	746.6058	PC(P-34:0) / PC(O-34:1)	$C_{42}H_{84}NO_7P$	M+H	LA	0.92	0.95
27	754.4784	PE(34:2)	$C_{39}H_{74}NO_8P$	M+K	HA	1.15	1.05
28	768.4940	PC(32:2)	$C_{40}H_{76}NO_8P$	M+K	HA	1.86	1.77
29	780.4940	PE(36:3)	$C_{41}H_{76}NO_8P$	M+K	HA	1.29	1.07
30	848.4627	PE(42:11)	$C_{47}H_{72}NO_8P$	M+K	HA	3.09	3.16
31	865.4393	PGP(34:2)	$C_{40}H_{76}O_{13}P_2$	M+K	HA	2.55	2.48
32	892.5253	PC(42:10)	$C_{50}H_{80}NO_8P$	M+K	HA	2.40	1.78
33	900.5151	PS(42:7)	$C_{48}H_{80}NO_{10}P$	M+K	HA	1.50	1.39
34	919.7151	TG(54:5)	$C_{57}H_{100}O_6$	M+K	LA	1.96	3.75
35	943.7151	TG(56:7)	$C_{59}H_{100}O_6$	M+K	LA	1.72	2.84
36	947.7464	TG(56:5)	$C_{59}H_{104}O_6$	M+K	LA	1.93	2.33

Supplemental Table S6: List of differentially abundant endogenous metabolites (tentative assignments) between wild-type (WT) and *Pex11a* knockout (KO) mouse heart tissue sections using high-resolution mass spectrometry imaging.

Abbreviations.: WT: wild-type *Pex11a* mice; KO *Pex11a* knockout mice; *m/z*: mass-to-charge-number ratio; Tentative assignments: Identification based on high mass accuracy (≤3 parts per million, ppm) at MS1 level; [M + H]+: Protonated ion adduct; [M + Na]+: Sodiated ion adduct; [M + K]+: Potassiated ion adduct; HA: Relatively higher signal intensities in *Pex11a* KO compared to WT control mice; LA: Relatively lower signal intensities in *Pex11a* KO compared to WT control mice; CAR: Acylcarnitine or Carnitine ester; CAR(O): Hydroxy acylcarnitine or Hydroxy carnitine

ester; RA: relative abundance; RMSE: Root mean square error; LPC: Lysophosphatidylcholine; LPE: Lysophosphatidylethanolamine; PC: Phosphatidylcholine; PE: Phosphatidylethanolamine; PS: Phosphatidylserine; PGP: Phosphatidylglycerolphosphate; DG: Diglyceride; TG: Triglyceride; P- / O-: Ether lipid
