# Supplementary material for: The Knockout of PEX11a Results in Mild Peroxisomal Dysfunction and Lowered Cardiac Recovery Following Langendorff-Mediated Ischemia–Reperfusion in Mice
Source: Cells. 2025 Dec 20;15(1):12. doi: 10.3390/cells15010012 (PMC12786121; doi:10.3390/cells15010012)
Supplement: Supplementary file 1 [file cells-15-00012-s001.zip › Supplemetal material/Supplemental Material and Methods.pdf]

Supplemental Material and Methods

The knockout of PEX11a results in mild peroxisomal dysfunction and impaired cardiac recovery following Langendorff-mediated ischemia-reperfusion in mice

Claudia Colasante[1*], Jiangping Chen[2], Vannuruswamy Garikapati[3], Bernhard Spengler[4], Klaus-Dieter Schlüter[5], Eveline Baumgart-Vogt[6*]

[1] Institute for Anatomy and Cell Biology, Justus Liebig University, Aulweg 123, 35392 Giessen, Germany; claudia.colasante@anatomie.med.uni-giessen.de

[2] Institute for Anatomy and Cell Biology, Justus Liebig University, Aulweg 123, 35392 Giessen, Germany; UKGM Giessen, Medical Clinic and Polyclinic II, Klinikstraße 33, 35392 Gießen, Germany; jiangping.chen@anatomie.med.uni-giessen.de

[3] Institute for Anatomy and Cell Biology, Justus Liebig University, Aulweg 123, 35392 Giessen, Germany; Max Planck Institute of Molecular Cell Biology and Genetics, Pfotenhauerstrasse 108, 01307 Dresden, Germany; garikapa@mpi-cbg.de

[4] Institute of Inorganic and Analytical Chemistry, Justus Liebig University, Heinrich-Buff-Ring 17, 35392 Giessen, Germany; bernhard.spengler@anorg.chemie.uni-giessen.de

[5] Institute for Physiology, Justus Liebig University, Aulweg 129, 35392 Giessen, Germany. klaus-dieter.schlueter@physiologie.med.uni-giessen.de

[6] Institute for Anatomy and Cell Biology, Justus Liebig University, Aulweg 123, 35392 Giessen, Germany; eveline.baumgart-vogt@anatomie.med.uni-giessen.de

***** Correspondence: C.C.: claudia.colasante@anatomie.med.uni-giessen.de; E.B.V.: eveline.baumgart-vogt@anatomie.med.uni-giessen.de

Detailed Material and Methods

2.1 Animals and ethical statement

Anesthesia and euthanasia of the mice and dissection of the heart were approved by the local authorities conforming to the Federal Act on the Protection of Animals (§4 section 3 of the Federal Act on the Protection of Animals - TSchG -) with the university internal classification number JLU-Nr.: 616_M (Project ID: 1016 Peroxisomen). For the present study the B6.129-Pex11a^{tm1Sjg} (*Pex11a* knockout) mice were kindly provided by Dr. Xiaoling Li [45]. Mice were bred to obtain the required wild-type (WT) or *Pex11a* knockout (KO) and genotyped as previously described [45] using DNA from ear-tissue and PCR with the primers P10, P11 and PNeo (Supplemental Table S1) (Supplemental Figure S1). The minimal animal number allowing the detection of significant changes for each experimental setup was chosen according to similar experimental setups previously published by our group [22-29] and in accordance with the 3R guidelines.

Animals were housed under standard conditions (12 h light/dark cycle) with free access to food and water. Animals were anaesthetized using 4 % isoflurane for 2 min in an anesthesia box connected to a vaporizer. Depth of anesthesia was assessed by monitoring the hind limb pedal retraction reflex and tail pinch reflex after rapid positioning of the mice on the dissection table. Animals were euthanized by a well-trained individual only after sufficient depth of anesthesia had been achieved, using the cervical dislocation method. To this purpose mice were placed facing down on the dissection table and held by the tail with one hand. The free hand was used to position and press a strong metal rod against the base of the skull. To separate the cervical vertebrae from the skull, the body of the mice was quickly pulled backwards and upwards by the tail. The separation of the vertebrae was confirmed by manual palpation.

2.2 RNA isolation and RT-qPCR

Sacrificed animals (3 animals per genotype, except for the qPCR of cardiac markers after I/R where 4 animals were used) were perfused for 30 s anterogradely with PBS through the left ventricle. Following perfusion, the heart was dissected and the left ventricle excised and shock-frozen in RNAzol® (Sigma-Aldrich). For RNA isolation, 50 mg tissue were shredded using an Ultra-Turrax and the total RNA was extracted according to the RNAzol® manufacturer´s protocol. The concentration and purity (230/260 > 1,7) of the RNA were analyzed using the NanoDrop ND-2000 (Peqlab). First-strand cDNA was synthesized from 1 µg RNA using random primers, dNTPs and 50 U MultiScribe™ reverse transcriptase (Applied Biosystems) in a final volume of 20 µl according to the manufacturer´s protocol.

For RT-qPCR, 1 µl 1:10-diluted cDNA, 0.5 µl forward and 0.5 µl reverse primers (Supplemental Table S2) and 3 µl water were mixed with 5 µl SsoAdvanced™ Universal SYBR®Green Supermix (Bio-Rad). All samples were run in duplicates in the IQ5 iCycler (Bio-Rad Laboratories) using the following protocol: 2 min at 95 °C (denaturation), 42 cycles of 15 s at 95 °C (denaturation), 30 s at 65 °C

(annealing) and 30 s at 72 °C (extension). Calculations of the relative gene expression were done by the $2^{-\Delta\Delta ct}$ method using b-actin as an internal standard [46].

2.3 Western blot analyses

Sacrificed animals (at least 3 mice per genotype. Exact number of mice used per individual antibody can be read from the graphical representation of the western blot densitometric analysis) were perfused for 30 s anterogradely with PBS through the left ventricle. The heart was dissected and the left ventricle excised and shock-frozen in 1 ml homogenization buffer (150 mM NaCl, 0.1% Triton X-100, 50 mM Tris-HCl, pH 8.0) containing protease inhibitor (Serva).

For protein lysates, 100 mg of tissue were resuspended in 1 ml fresh homogenization buffer containing protease inhibitor (Serva) and shredded using an Ultra-Turrax. Samples were then passed through a Dounce homogenizator and spun at 500 x g for 5 min to remove cell debris and nuclei. The protein concentration was determined using the Bradford assay (Bio-rad) according to the manufacturer´s protocol.

Proteins (10 µg) were separated on a 12 % SDS-PAGE and transferred to PVDF-membranes (Millipore) by tank blotting in Towbin buffer (25 mM Tris, 192 mM Glycine, 20 % v/v Methanol). Membranes were blocked for 1 h in 5 % fat-free milk in TBS-Tween (50 mM Tris, 150 mM NaCl, 0.1 % Tween 20), followed by incubation with the primary antibody (Supplemental Table 3) for 1 h at room temperature. The membranes were washed in TBS-Tween and incubated with the secondary antibody (Supplemental Table 4) for 1 h at room temperature. Detection was performed using the ECL detection kit (Bio-Rad) for secondary antibodies conjugated to horseradish peroxidase. Immunolabelled protein bands were detected by exposing the membranes to CL-Exposure Films (Thermo). Several exposure times were used to obtain images in which the bands were not oversaturated.

2.4 Paraffin embedding for histological studies

Sacrificed mice were perfused for 1 min anterogradely with PBS through the LV, followed by 3 min perfusion using 4 % paraformaldehyde (PFA), 2 % sucrose/PBS. The hearts were dissected, immersion-fixed overnight in 4 % PFA, 2 % sucrose/PBS and embedded in paraffin using a Leica TP1020 automated vacuum infiltration tissue processor as previously described [23]. Paraffin blocks were cut with a Leica RM2135 rotation microtome.

2.5 Immunofluorescence staining and image acquisition

For immunofluorescence analysis 2 µm PFA fixed, paraffin embedded tissue (FFPE) sections were cut with a rotation microtome and mounted on Superfrost Plus (+) slides (Langenbrick). Deparaffinized and rehydrated sections were processed for antigen retrieval with 0.01 % trypsin for 10 min at 37 °C, followed by microwaving for 3 x 5 min at 900 W in 10 mM citrate buffer, pH 6.0. Blocking of non-specific protein binding sites was performed by incubation with 4 % bovine serum

albumin (BSA) in TBS-Tween. Sections were incubated with primary antibodies (Supplemental Table 3) overnight at room temperature with 1 % BSA in PBS and then with the appropriate fluorochrome-conjugated secondary antibodies in 1 % BSA in PBS for 2 h (Supplemental Table 5). Finally, the sections were counterstained with the nuclear dye DAPI (1 µg/ml) (Sigma). Images were acquired using a fluorescence microscope (Laser scanning microscope lsm_710, Zeiss) equipped with a DC40 camera and processed using Photoshop CS5 [23]. For each genotype images were taken and analyzed morphometrically from 3 mice per genotype except for the PLIN2-, SOD2- and Complex IV-stainings where 4 mice per genotype were used. Image acquisition was blinded, with the genotype of the mice on the slides concealed and randomly assigned numbers substituted for it. The numbers were used to facilitate the reassociation of the acquired images to the genotype during the process of image analysis.

2.6 Histological stainings

2.6.1 Hematoxylin and eosin (HE) staining

5 µm PFA fixed, paraffin embedded tissue sections from at least 3 animals per genotype (Exact number of mice used per individual experimental setup can be read from the graphical representation of the measurements performed on the HE-stained sections) were first deparaffinized with xylene and then re-hydrated in alcohol in a downstream dilution series (99 %, 99 %, 96 %, 80 %, 70 %, 50 %) 3 min each. The objective slides were rinsed in dH_2O for 3 min and incubated in Mayer´s hematoxylin solution for 2 min. The slides were rinsed for 10 min in running tap water, placed in dH_2O for some seconds and counterstained with Eosin Y solution containing 0.2 % glacial acetic acid for 30 s. Samples were rinsed shortly in dH_2O followed by sample dehydration and clearance by immersion in 50 %, 70 %, and 80 % ethanol (1 min each), 96 % ethanol (3 min), and 99 % ethanol (2 times 3 min). Finally, the slides were immersed in xylene, allowed to dry and mounted in DEPEX. Images were taken using a Leica DM 750 light microscope. Image acquisition was blinded, with the genotype of the mice on the slides concealed and randomly assigned numbers substituted for it. The numbers were used to facilitate the reassociation of the acquired images to the genotype during the process of image analysis.

2.6.2 Azan staining

5 µm PFA fixed, paraffin embedded tissue sections from 3 mice per genotype were first deparaffinized with xylene and then re-hydrated in alcohol in a downstream dilution series (99 %, 99 %, 96 %, 80 %, 70 %, 50 %) 3 min each. The slides were incubated for 15 min in azocarmine G solution (0.1 % azocarmine G w/v, 1 % glacial acetic acid) preheated to 56 °C and then rinsed in dH_2O. The slides were then transferred to anilinoethanol (0.1 % v/v) and incubated until only the nuclei appeared stained. Samples were then washed for 1 min in 1 % v/v glacial acetic acid in ethanol and incubated for 2 h in 5 % v/v phosphotungstic acid. After rinsing with dH_2O the slides were stained for 2 h in 1:3 diluted anilinblue-orange-G (0.5 % w/v aniline blue, 2 % w/v orange-G, 8 % v/v glacial

acetic acid). Then the slides were rinsed shortly in dH_2O followed by sample dehydration and clearance by immersion in 50 %, 70 %, and 80 % ethanol (1 min each), 96 % ethanol (3 min), and 99% ethanol (2 times 3 min). Finally, the slides were immersed in xylene, allowed to dry and mounted in DEPEX. Images were taken using a Leica DM 750 light microscope. Image acquisition was blinded, with the genotype of the mice on the slides concealed and randomly assigned numbers substituted for it. The numbers were used to facilitate the reassociation of the acquired images to the genotype during the process of image analysis.

2.6.3 Picro-sirius red staining

5 µm tissue sections from 3 mice per genotype were first deparaffinized with xylene and then re-hydrated in alcohol in a downstream dilution series (99 %, 99 %, 96 %, 80 %, 70 %, 50 %) 3 min each and lastly in water for 4 min. According to the manufacturer´s protocol (Morphisto, Offenbach, Germany), the slides were first incubated for 15 min in Weigert's iron hematoxylin solution, rinsed in H_2O for 9 min and then transferred to the picro-sirius-red solution for 60 min. Slides were then washed twice for 1 min in 30 % v/v glacial acetic acid, rinsed shortly in dH_2O and dehydrated and cleared by immersion in 50 %, 70 %, and 80 % ethanol (1 min each), 96 % ethanol (3 min), and 99 % ethanol (2 times 3 min). Finally, the slides were immersed in xylene, allowed to dry and mounted in DEPEX. Images were taken using a Leica DM 750 light microscope. Image acquisition was blinded, with the genotype of the mice on the slides concealed and randomly assigned numbers substituted for it. The numbers were used to facilitate the reassociation of the acquired images to the genotype during the process of image analysis.

2.6.4 PAS staining

5 µm tissue sections from 3 mice per genotype were first deparaffinized with xylene and then re-hydrated in alcohol in a downstream dilution series (99 %, 99 %, 96 %, 80 %, 70 %, 50 %) 3 min each and lastly in water for 2 min. According to the manufacturer´s protocol (Morphisto, Offenbach, Germany), the slides were first incubated for 20 min in a 1% solution of periodic acid, rinsed in H_2O for 3 min followed by two brief rinsing steps in dH_2O. Thereafter the slides were incubated for 20 min in Schiff´s reagent and then washed in H_2O for 5 min. Nuclei were stained using acidic Hematoxylin according to Mayer for 5 min. Slides were then rinsed H_2O for 3 min and dehydrated and cleared by immersion in 50 %, 70 %, and 80 % ethanol (1 min each), 96 % ethanol (3 min), and 99 % ethanol (2 times 3 min). Finally, the slides were immersed in xylene, allowed to dry and mounted in DEPEX. Images were taken using a Leica DM 750 light microscope. Image acquisition was blinded, with the genotype of the mice on the slides concealed and randomly assigned numbers substituted for it. The numbers were used to facilitate the reassociation of the acquired images to the genotype during the process of image analysis.

2.7 Electron microscopy (EM)

Mice (3 animals per genotype) were sacrificed by cervical dislocation and perfused for 30 s anterogradely with PBS through the left ventricle followed by 3 min perfusion, using 4 % PFA, 0.05 % glutardialdehyde (GDA), 2 % sucrose in 0.1 M pipes. Thereafter, heart left ventricles were dissected and cut into 1-mm^3 blocks. Immersion-fixation was performed overnight at 4 °C in either i) 1 % GDA in 0.1 M cacodylate buffer for standard-EM, or ii) in 0.05%, GDA, 4 % PFA, 0.1 M Pipes for immune-EM.

For standard-EM the tissues were washed with 0.1 M cacodylate buffer, pH 7.4, and fixed with 1 % aqueous osmiumtetroxide at room temperature. Osmicated tissues were dehydrated in a graded series of ethanol and propylene oxide and embedded in the epoxy resin 812 (Agar, Essex, England). Embedded tissue blocks were trimmed with a diamond trimmer (Reichert TM 60, Austria). Semithin sections were cut, and selected regions were subjected to ultrathin sectioning (80 nm) with a Reichert Ultracut S ultramicrotome (Leica, Nussloch, Germany). Ultrathin sections were collected on copper grids and contrasted with a half saturated aqueous solution of uranylacetate for 5 min and lead-citrate (Reynolds Reagent) for 1 min, followed by examination in a LEO 906 transmission electron microscope (LEO Electron Microscopy, Oberkochen, Germany). Image acquisition was blinded, with the genotype of the mice not indicated on the grids and randomly assigned numbers substituted for it. The numbers were used to facilitate the reassociation of the acquired images to the genotype during the process of image analysis.

For the immuno-EM post-embedding method, the heart tissue was rinsed in Pipes buffer and placed directly into 70 % ethanol to increase antigenic yield. After two washes of 60 min each in 70 % ethanol the tissue was transferred into LR white resin and polymerization at 50 °C. The embedded tissue was trimmed with a diamond trimmer (Reichert TM 60, Austria). Semi-thin sections were cut, and selected regions were subjected to ultrathin sectioning (80 nm) with a Reichert Ultracut S ultramicrotome (Leica, Nussloch, Germany) and collected on 100-mesh nickel grids coated with 1 % formvar. The sections were then incubated on droplets of 0.1 M glycine in PBS to quench the remaining free aldehyde groups. Blocking of the non-specific binding sites was achieved with 1 % BSA in PBS, 0.05 % Tween (BSA-PBS/Tween). Primary antibody against catalase (Supplemental Table 3) was diluted in 0.1 % BSA-c (Aurion) in PBS/Tween and the labelling was done overnight at room temperature in a moist chamber. The next morning, the grids were washed in a series of BSA-PBS/tween and thereafter incubated with anti-rabbit Aurion ultra small immunogold conjugated F(ab`) fragments diluted in BSA-PBS/Tween for 2 h at room temperature, washed in BSA-PBS/Tween and then in PBS. The secondary antibody reaction was fixed after incubation with 1 % GDA in PBS. Ultra-small immunogold particles were intensified with Danscher silver enhancement after washing in deionized water. Rinsed grids were then contrasted with a half saturated aqueous solution of uranyl acetate for 5 min and lead-citrate (Reynolds Reagent) for 1 min, followed by examination in a LEO 906 transmission electron microscope (LEO Electron Microscopy, Oberkochen, Germany) [12,27]. Image acquisition was blinded, with the genotype of the mice not indicated on the grids and randomly assigned numbers substituted for it. The numbers were used to

facilitate the reassociation of the acquired images to the genotype during the process of image analysis.

2.8 Langendorf reperfusion

Langendorff reperfusion was performed as previously described by Boengler *et al* [47]. 8 wild-type and 8 *Pex11a* mice were anesthetized with 4 % isoflurane and euthanized by cervical dislocation. Thereafter, hearts were rapidly excised, and the aorta was cannulated for retrograde perfusion with an aortic cannula for mouse hearts (Ø 1 mm, Hugo Sachs Elektronik-Harvard Apparatus, March, Germany) connected to a Langendorff perfusion system. Hearts were perfused with 37 °C warm modified Krebs Henseleit buffer (118 mM NaCl, 4.7 mM KCl, 0.8 mM $MgSO_4$, 1.2 mM KH_2PO_4, 5 mM glucose, 2.5 mM $CaCl_2$, 25 mM $NaHCO_3$, 1.9 mM pyruvate continuously gassed with 95 % O_2, 5 % CO_2, pH 7.4) at a constant perfusion pressure of 70 mmHg (transduced by a replacement transducer head for APT300 pressure transducer, Hugo Sachs electronic-Harvard apparatus). A balloon was inserted into the left ventricle and was connected to a pressure transducer (Combitrans 1-fach Set Mod.II University Giessen, B. Braun, Melsungen, Germany) for assessment of ventricular performance. The balloon was inflated to yield a left ventricular end-diastolic pressure of 12–14 mmHg, which was kept constant thereafter. Hearts were paced during measurements at 600 bpm. Left ventricular developed pressure (LVDP, systolic pressure—diastolic pressure) was recorded. Perfused hearts were left to stabilize for 5 min. Ischemia was induced for 45 min by stopping flow and pacing. After 120 min of reperfusion, the hearts were removed from the perfusion apparatus. The left ventricles were dissected and immediately fixed in 4 % PFA for histological studies or shock frozen for RNA (with addition of RNAzol) or protein analysis.

2.9 Sample preparation for MALDI MS imaging

Heart tissues from wild-type and *Pex11a* knockout mice were dissected, embedded in gelatin (European Pharmacopoeia grade, VWR International, Radnor, PA, USA), snap-frozen in liquid nitrogen and stored at -80 °C until further use. Tissue sections were cut with an equal thickness of 15 µm at -20 °C with a cryostat (CM 3050 S cryostat, Leica Microsystems, Nussloch, Germany), thaw-mounted on numbered glass slides (ground edges, SuperFrost, R. Langenbrinck, Emmendingen, Germany) and stored at -80 °C until further analysis.

On the day of analysis, the slide box was placed on dry ice and the individual sections were quickly photographed using phase contrast in a light microscope. Wild-type and *Pex11a* knockout sections displaying similar thickness (similar phase contrast density) in the images were chosen pairwise and dehydrated in a desiccator for 15-20 min at room temperature to avoid condensation of water vapor. Then, phase-contrast images of the tissue sections were taken with a Keyence VHX-5000 digital microscope (Keyence Deutschland GmbH, Neu-Isenburg, Germany). For matrix application, a dedicated automatic pneumatic ultrafine sprayer (SMALDIPrep, TransMIT GmbH, Giessen, Germany) was used. 120 µl of a freshly prepared 2,5-dihydroxybenzoic acid (DHB) matrix solution

(30 mg/ml in acetone:water 1:1 v/v, 0.1 % TFA, both spectroscopy grade, Merck, Darmstadt, Germany, water LC-MS grade, VWR International, Radnor, PA, USA) was deposited homogeneously on the tissue surfaces with optimized spray conditions [48]. The homogeneity and size of the matrix crystals were checked with an optical microscope before MSI analyses.

2.10 MALDI MS imaging data acquisition and processing

After matrix application, mass spectrometry imaging (MSI) experiments were performed using a high-resolution atmospheric-pressure scanning microprobe matrix-assisted laser desorption/ionization ion source ("AP-SMALDI10", TransMIT GmbH, Giessen, Germany), coupled to a Fourier transform orbital trapping mass spectrometer (Q Exactive, Thermo Fisher Scientific GmbH, Bremen, Germany) as described earlier [48]. In brief, both wild-type and *Pex11a* knockout mouse heart tissue sections (biological and technical replicates) were measured on successive days with identical sample preparation and experimental conditions. The mass spectrometer was operated in positive-ion mode (target voltage set to +4.3 kV) with a scanning step size of 25 µm, a mass range of *m/z* 250–1500 and mass resolution of 140,000 at *m/z* 200. Calibration of mass spectra was performed by internal "lock mass" calibration with DHB matrix cluster peak (*m/z* 716.12462), resulting in a mass accuracy of better than 3 ppm root-mean-square error (RMSE).

The individual MSI raw datasets were converted to imzML (a common data format for MS imaging) and stitched together. The resulting files were analyzed, and MS ion images were generated without normalization and with total ion count (TIC) normalization using an open-source software "MSiReader" v1.01 (http://www.msireader.com/) with a bin width of Δ *m/z* = ±5 ppm. The endogenous metabolites were tentatively assigned based on accurate ion mass values (MS1 level annotation), by using databases such as LIPID MAPS (www.lipidmaps.org), human metabolome database (www.hmdb.ca), and METLIN (www.metlin.scripps.edu) within Δ *m/z* = ±5 ppm and/or 0.0005 Da. The protonated [M + H]+, sodiated [M + Na]+ and potassiated [M + K]+ ion adduct species were considered for the annotations. To compare the reliable relative signal intensities of metabolites/lipids among the tissue sections, only MS ion images that display similar trends of differential abundances both with TIC and without normalization were considered [48].
